# Supplementary material for: Variability of plasmid fitness effects contributes to plasmid persistence in bacterial communities
Source: Nat Commun. 2021 May 11;12:2653. doi: 10.1038/s41467-021-22849-y (PMC8113577; doi:10.1038/s41467-021-22849-y)
Supplement: Supplementary file 1 — Supplementary Information [file 41467_2021_22849_MOESM1_ESM.pdf]

Supplementary Information

Variability of plasmid fitness effects contributes to plasmid persistence in bacterial communities

Aida Alonso-del Valle[1], Ricardo León-Sampedro[1,2], Jerónimo Rodríguez-Beltrán[1,2], Javier DelaFuente[1], Marta Hernández-García[1,3], Patricia Ruiz-Garbajosa[1,3], Rafael Cantón[1,3], Rafael Peña-Miller[4,*], Álvaro San Millán[1,2,5*].

[1] *Servicio de Microbiología. Hospital Universitario Ramón y Cajal* and *Instituto Ramón y Cajal de Investigación Sanitaria*. Madrid, Spain.

[2] *Centro de Investigación Biológica en Red. Epidemiología y Salud Pública*, *Instituto de Salud Carlos III*. Madrid. Spain.

[3] *Red Española de Investigación en Patología Infecciosa*. *Instituto de Salud Carlos III*. Madrid. Spain.

[4] Center for Genomic Sciences, *Universidad Nacional Autónoma de México*, Cuernavaca, Mexico.

[5] Current address: *Centro Nacional de Biotecnología–CSIC*, Madrid, Spain.

* Correspondence: Álvaro San Millán, alvsanmillan@gmail.com ORCID: 0000-0001-8544-0387 and Rafael Peña-Miller, rafael.penamiller@gmail.com ORCID: 0000-0002-2767-0640

Supplementary Figure 1. Growth curves of wild-type isolates and pOXA-48-carrying transconjugants.

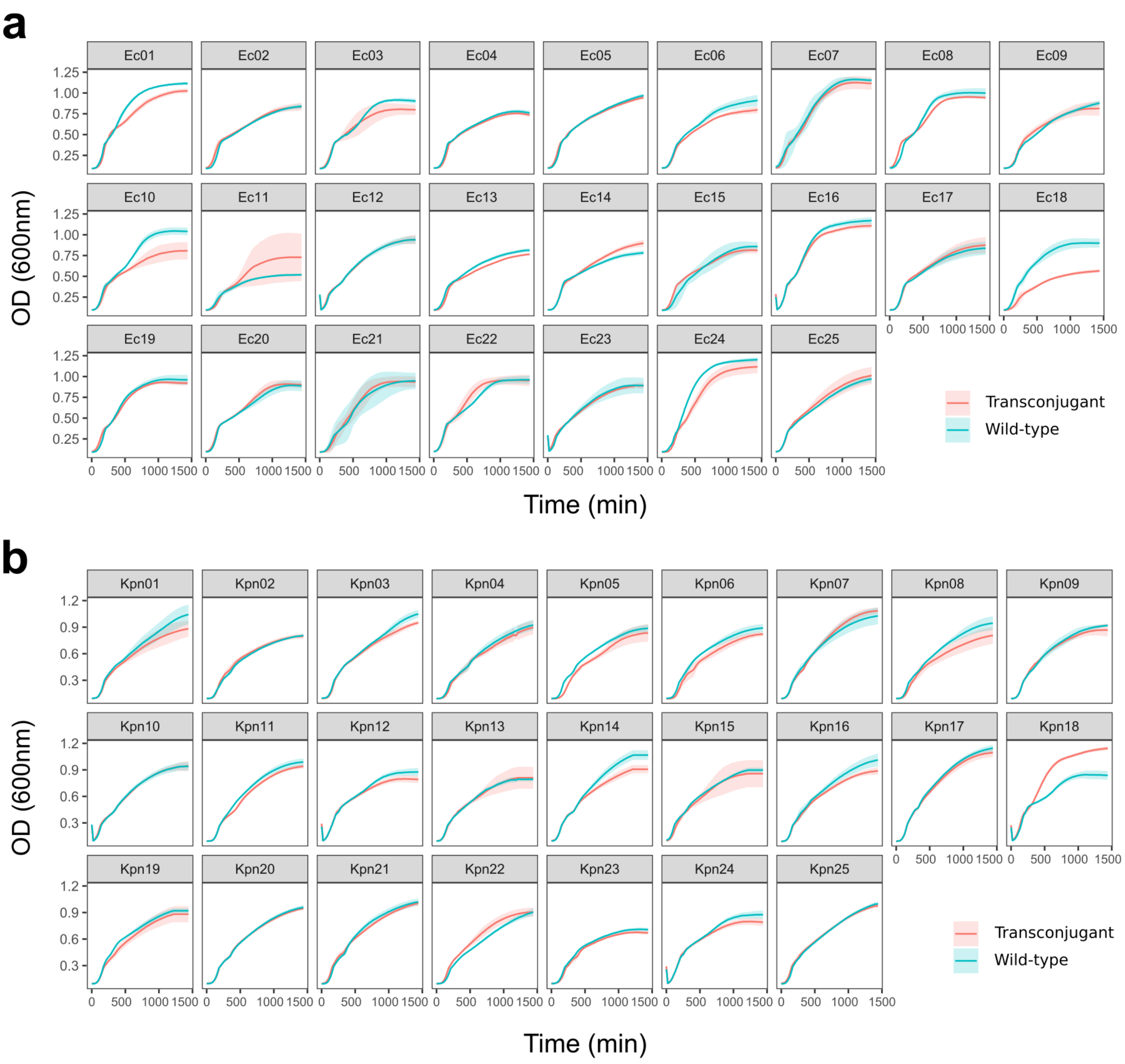

Growth curves of pOXA-48-free (wild-type, blue) and pOXA-48-carrying (transconjugant, red) for every (a) *E. coli* and (b) *Klebsiella* spp. analysed in this study. The lines represent the average of four and six biological replicates, for the wild type and transconjugants, respectively, and the shaded area indicates 95% confidence intervals.

Supplementary Figure 2. Independent growth curve parameters for each isolate.

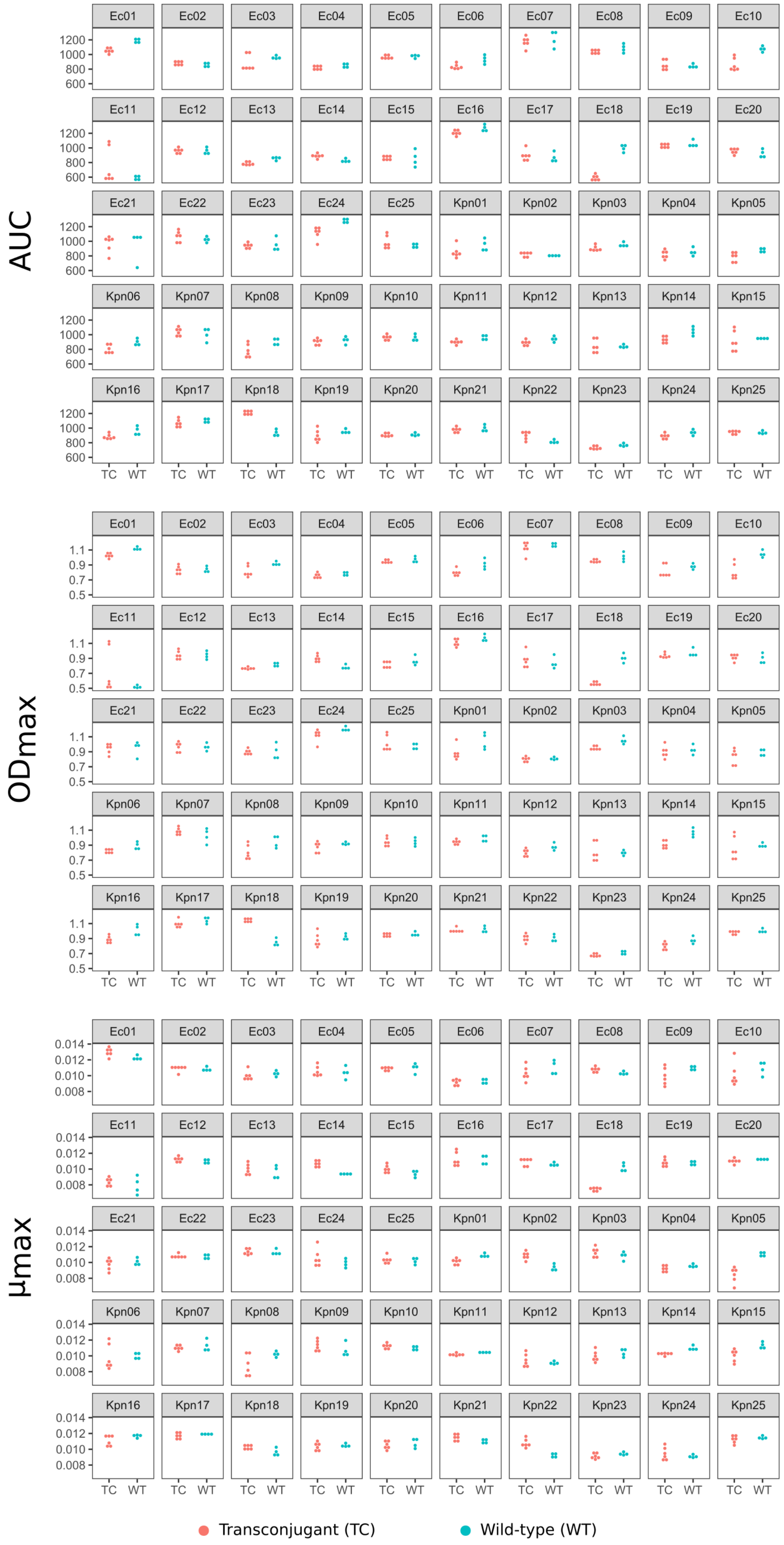

Values extracted from the independent biological replicates of the growth curves. (a) Area under the growth curve (AUC, measured in arbitrary units), (b) maximum optical density ($OD_{max,}$ measured as absorbance at 600 nm), and (c) maximum growth rate (μ_{max}, measured in OD_{600}/min). Points represent the results of each independent replicate for pOXA-48-free (wild-type, blue) and pOXA-48-carrying (transconjugant, red) isolates analyzed in this study. Four and six biological replicates of the growth curves were performed for the wild-type isolates and the transconjugants, respectively.

Supplementary Figure 3. Construction of plasmid pBGC.

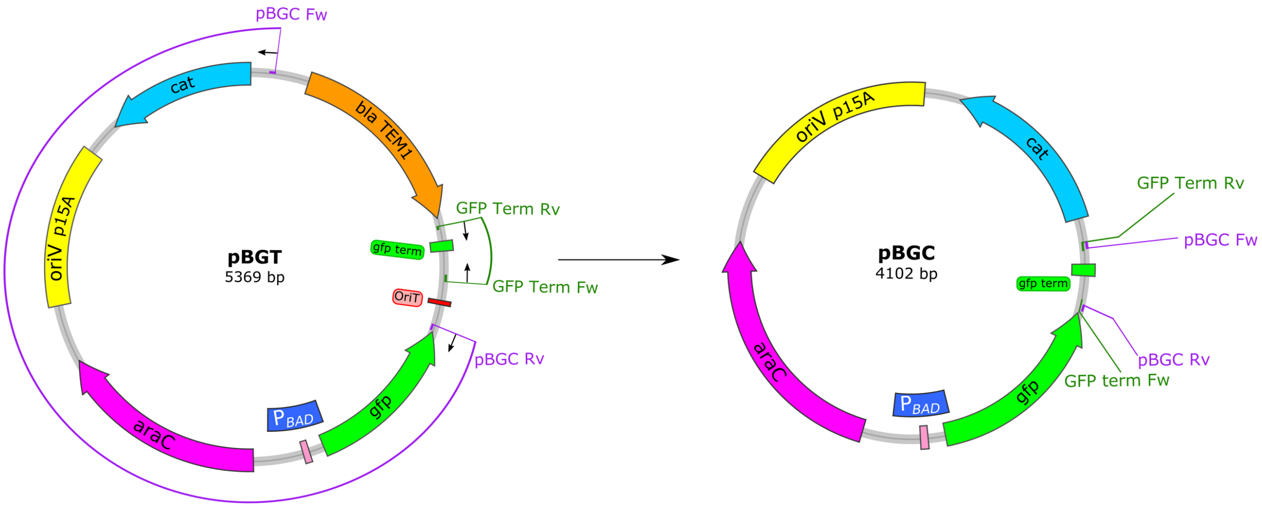

Schematic representation of the construction of plasmid pBGC (accession number MT702881) form plasmid pBGT[68]. Two segments of pBGT were amplified using primers with added cohesive ends (pBGC Fw/Rv and GFP Term Fw/Rv, Supplementary Table 4). pBGC plasmid resulted from the Gibson assembly of the amplified fragments. The reading frames for genes are shown as arrows, with the direction of transcription indicated by the arrowhead. The origin of replication (oriV), origin of transfer (oriT), and P_{BAD} promoter are also indicated.

Supplementary Figure 4. Correlation between relative growth curve parameters and relative fitness.

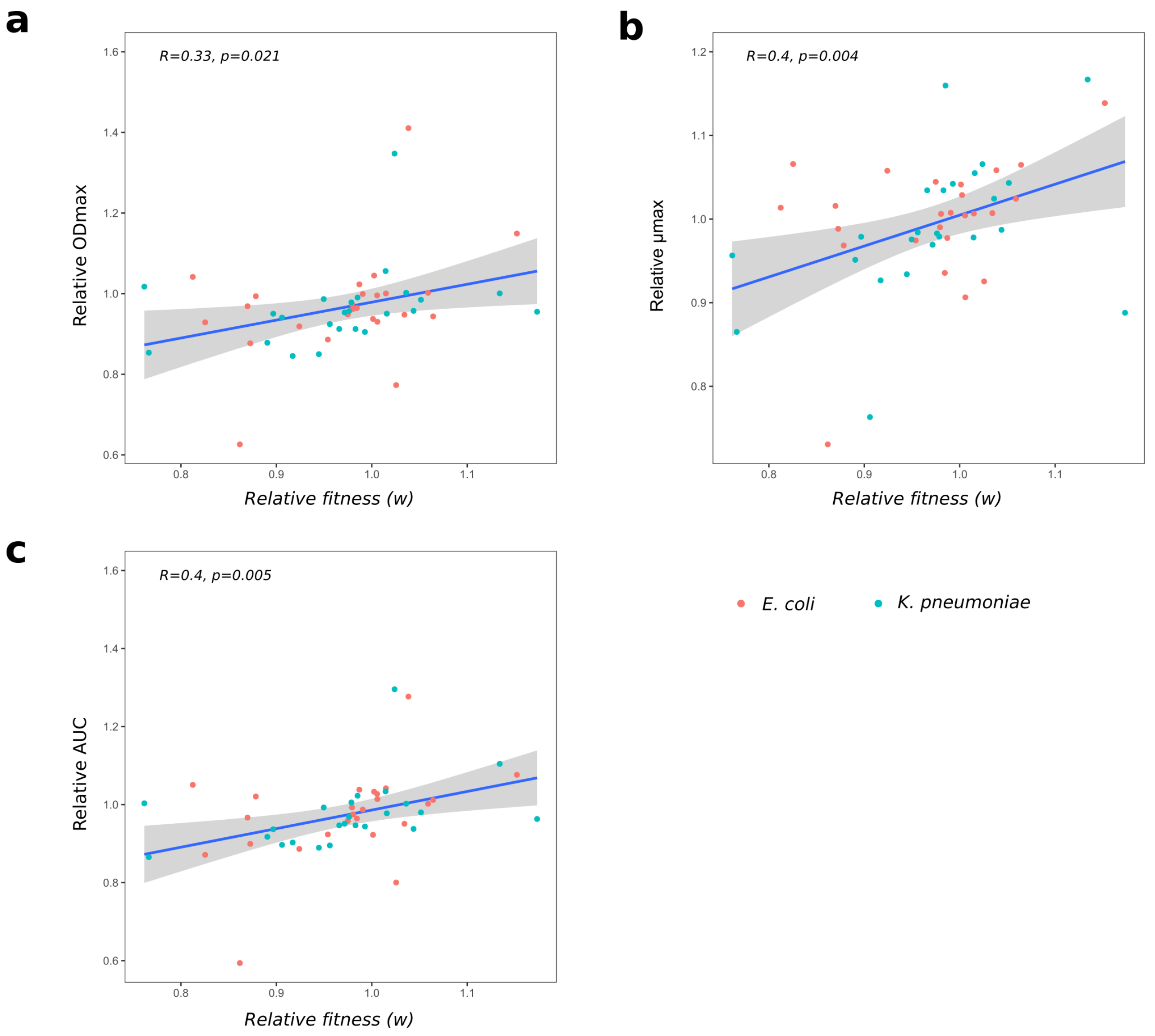

Correlation between relative growth curve parameters (a) maximum optical density (OD_{max}), (b) maximum growth rate (μ_{max}), and (c) area under the growth curve (AUC), and relative fitness values obtained from competition assays for each strain (n=25 biologically independent *E. coli* and 25 biologically independent *Klebsiella* spp. strains). The blue line represents the linear regression model and the grey shading represents 95% confidence intervals. Points represent each relative value (red, *E.* coli and blue, *Klebsiella* spp.). Pearson's product-moment correlation (*R*) and *p*-value are indicated (one-sided). As expected, maximum optical density, maximum growth rate and area under the growth curve are positively correlated with relative fitness. Source data are provided as a Source Data file.

Supplementary Figure 5. Local Indicator of Phylogenetic Association (LIPA) analyses.

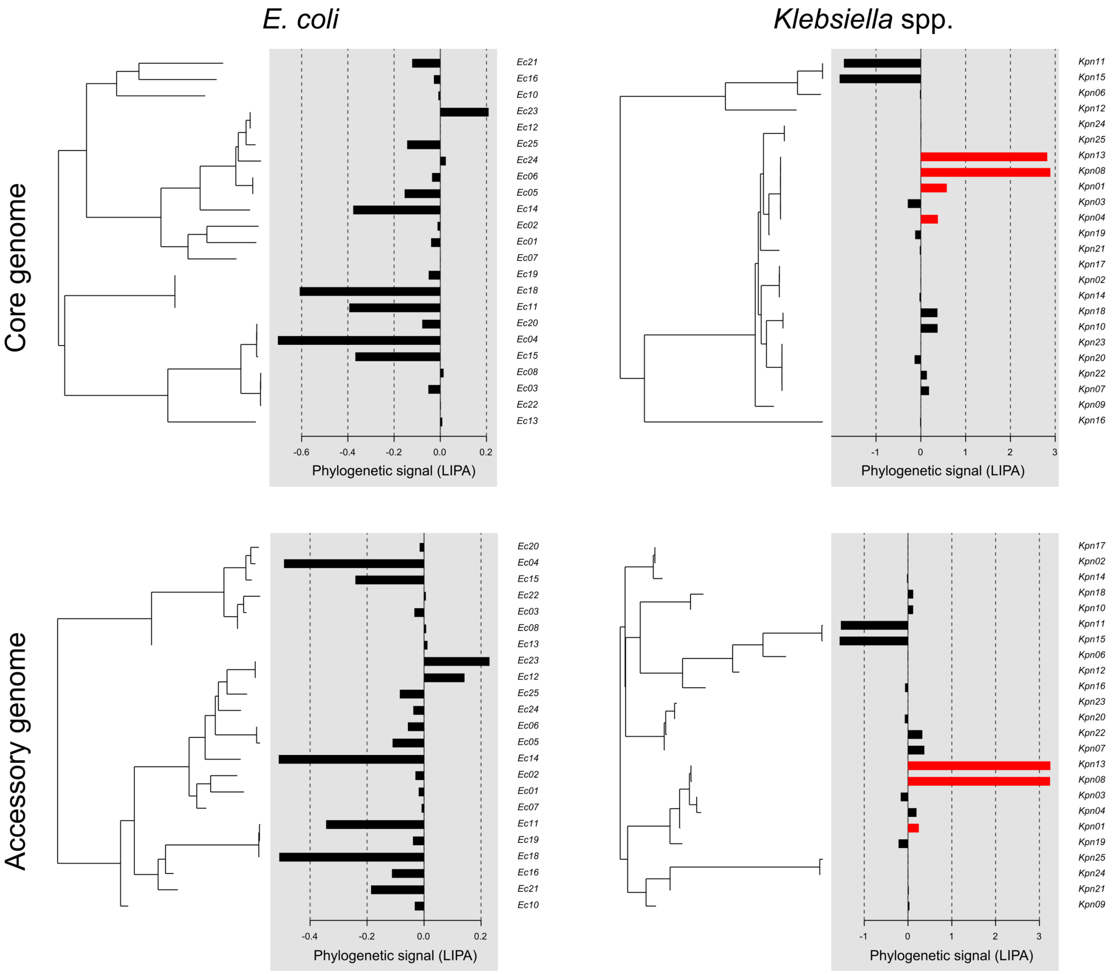

Phylogenetic trees for core (upper panels) and accessory (lower panels) genomes obtained for *E. coli* (left) and *Klebsiella* spp. (right). Bar plots show the LIPA score associated with each tip of the phylogeny, with higher values representing a stronger phylogenetic signal. Red colour indicates statistically significant LIPA scores (*i.e.* phylogenetic signal, Supplementary Table 3).

Supplementary Figure 6. Presence of IncFIA or IncH1B plasmids and absence of IncFIB plasmids are associated with high pOXA-48_K8 costs in *Klebsiella* spp.

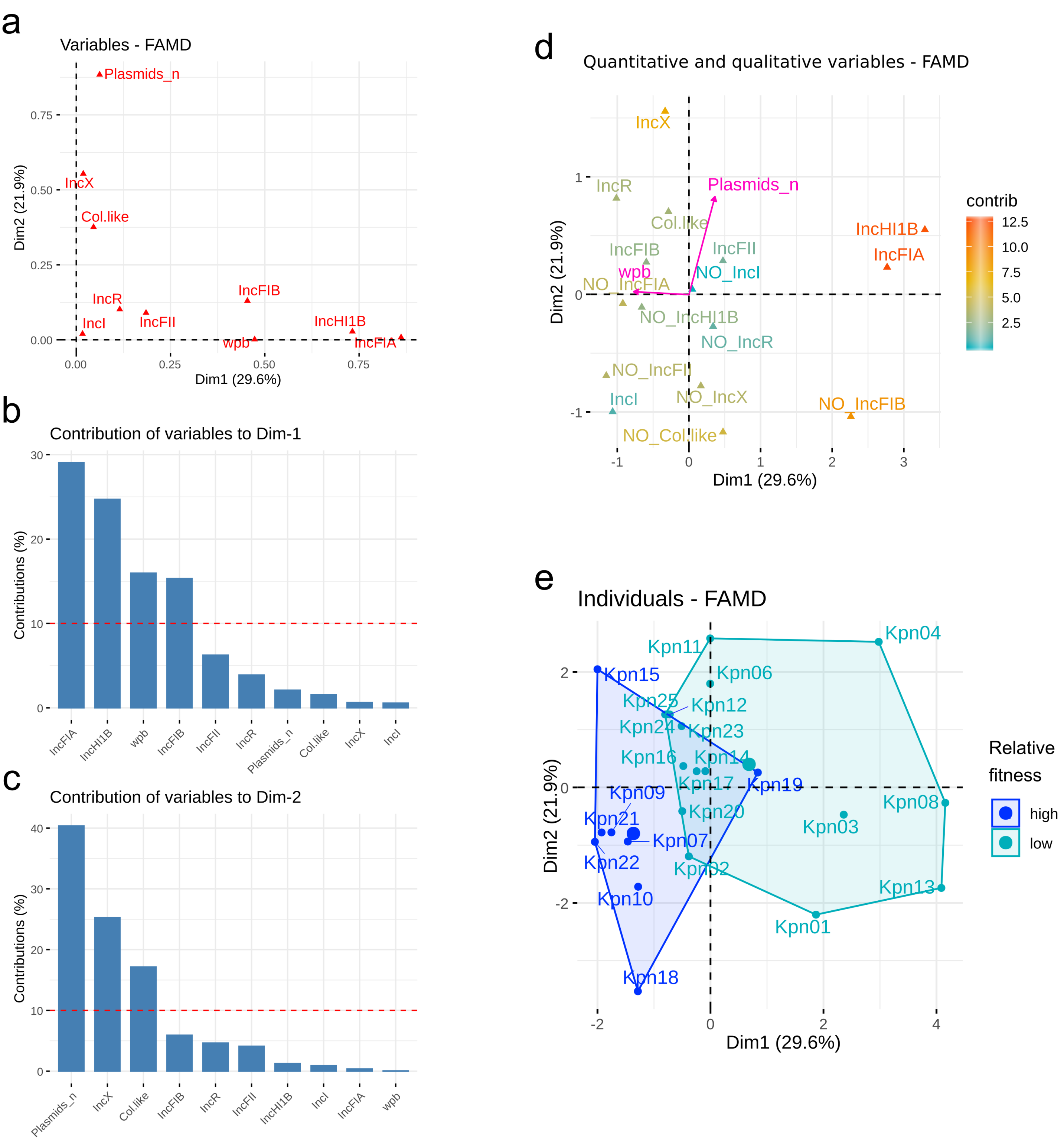

Factor Analysis of Mixed Data (FAMD) combining qualitative (presence/absence of plasmid families shown in Figure 4) and quantitative (number of plasmid -Plasmids_n- and relative fitness of plasmid-carrying isolates –wpb-) variables to explore the association between all variables for *Klebsiella* spp.

isolates. This analysis aims to reduce the dimensionality of the data to achieve a lower number of principal components represented by dimensions. These dimensions are linear combinations of the original variables and are not correlated with each other. Quantitative and qualitative variables are normalized during the analysis in order to balance the influence of each set of variables. (a) Correlation between both quantitative and qualitative variables and the principal dimensions. Panels (b) and (c) show the contribution of variables to the dimensions 1 (Dim-1, panel b) and 2 (Dim-2, panel c). The percentage between brackets indicates the proportion of the variability explained by each dimension. The red dashed line on in panels b and c indicates the expected contribution of each variable if the contributions were uniform. Variables with a contribution higher than the expected average have a significant association with that dimension. For Dim-1 the plasmid variants IncFIA, IncHI1B, the fitness effect (wpb) and IncFIB have a higher contribution, while for Dim-2 the plasmid number and variants IncX and Col.like have a higher contribution. (d) Inertia of both quantitative and qualitative variables through the two dimensions. The percentage between brackets indicates the proportion of the variability explained by each dimension. For the qualitative variables (plasmid variants), the absence of plasmid belonging to each plasmid family is indicated with a “NO_” before the name of the category. The contribution of each variable is show by a gradient of colour. For the quantitative variables the arrows show the relationship between variables and the correlation and influence between variables and the two dimensions. The relative fitness of plasmid-carrying isolates (wpb) shows a higher influence on Dim-1 while the plasmid number (Plasmids_n) shows a higher influence on Dim-2. Note that the presence of plasmids belonging to the IncFIA or IncH1B families, and the absence of plasmids belonging to the IncFIB family, are associated with high pOXA-48_K8 costs. (e) Factor map for the individuals between the two dimensions. Isolates with similar profiles are close to each other. The individuals are classified and coloured according to pOXA-48_K8 fitness effects: relative fitness of plasmid-carrying bacteria > 1 (High; no plasmid-associated costs) and < 1 (Low; plasmid-associated costs). The thicker points indicate the mean point (barycentre) of each group. Source data are provided as a Source Data file.

Supplementary Figure 7. *In silico* competition experiments.

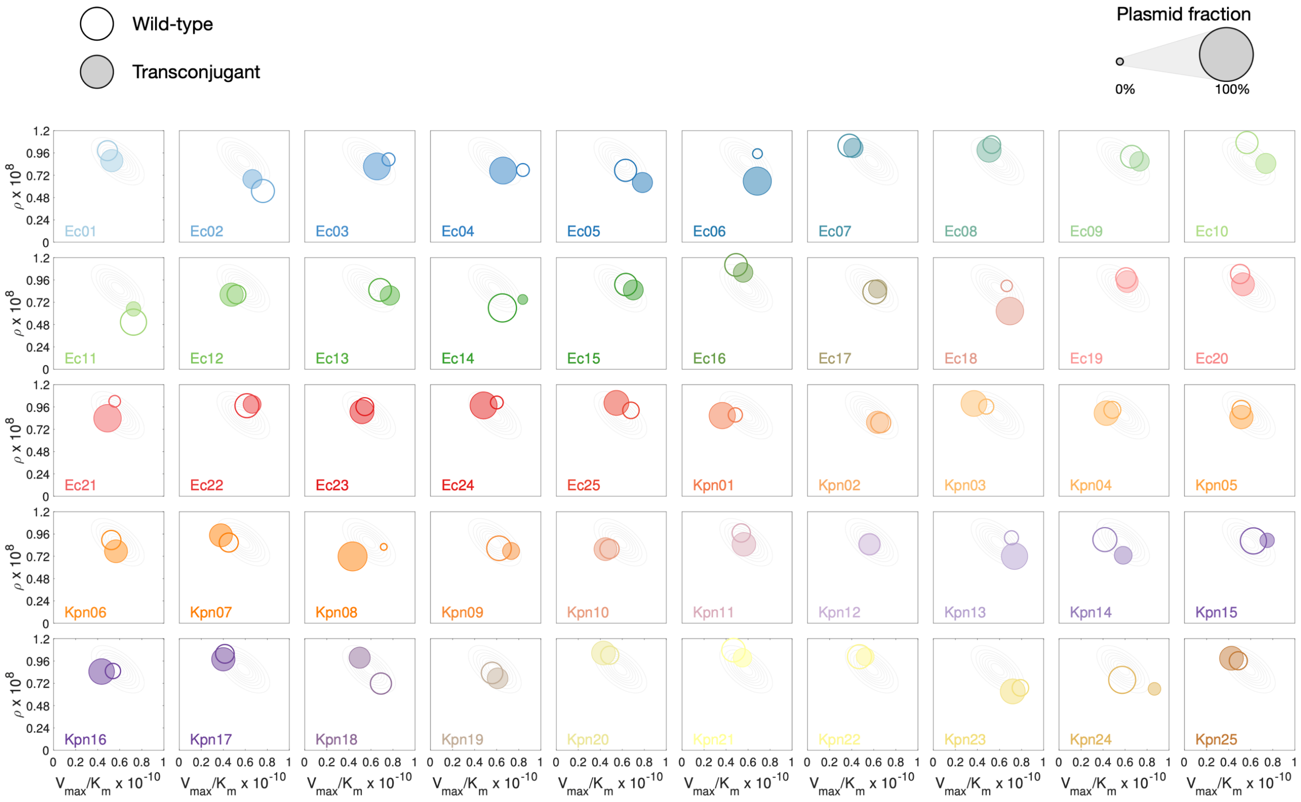

Each box represents a theoretical pair-wise competition experiment between plasmid-free (open circles) and plasmid-bearing cells (filled circles). The diameter of each circle is proportional to the relative fraction of the population, a value estimated by numerically solving the model for $T = 24$ with parameter values obtained from the posterior distribution of each strain. Horizontal axis represents the specific affinity (V_{max}/K_m) and the vertical axis the cell's resource conversion rate (ρ).

Supplementary Figure 8. Comparison between *in silico* and experimental competition experiments

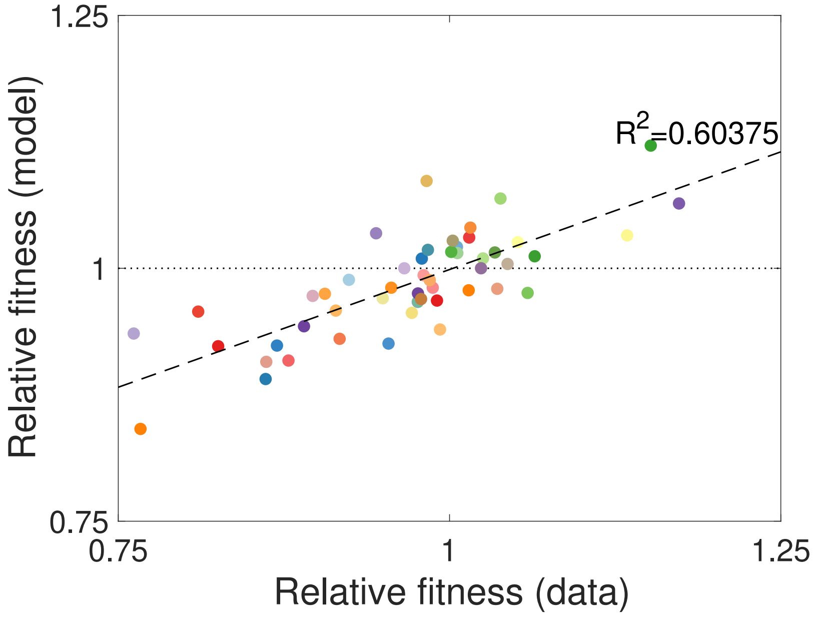

Comparison between relative fitness values obtained experimentally and numerical competition experiments performed using the population dynamics model. Dotted line represents linear regression (R^2= 0.603), suggesting that the model accurately predicts the outcome of competition experiments from individual growth dynamics. Source data are provided as a Source Data file.

Supplementary Figure 9. Plasmid stability as a function of the conjugation rate and plasmid cost.

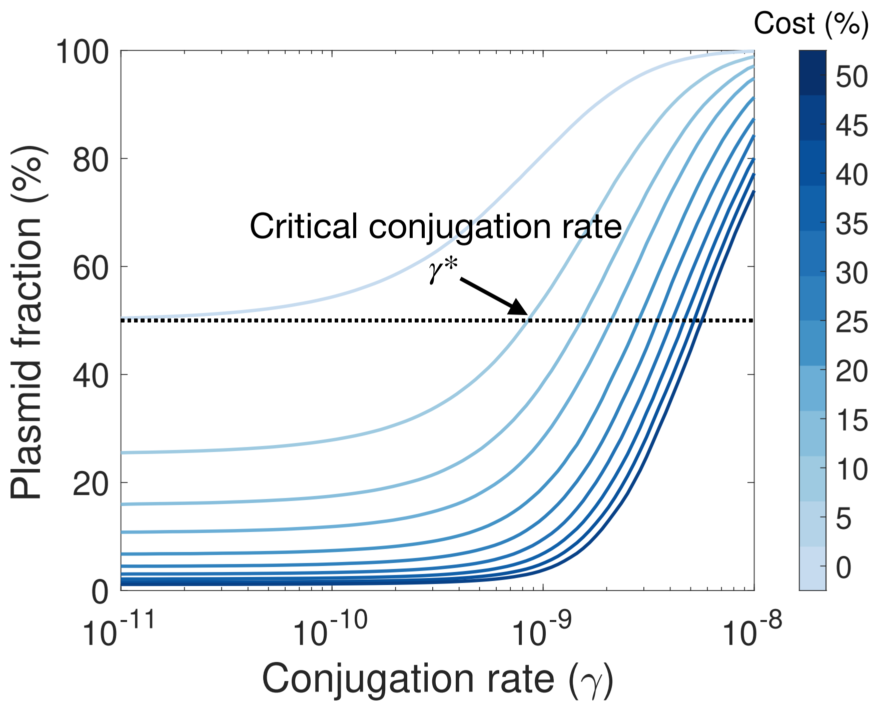

Numerical results obtained by simulating $1x10^4$ pair-wise competition experiments between plasmid-bearing and plasmid-free cells using the population dynamics model. Lines represent the mean plasmid fraction observed at T=24 for different conjugation rates and fitness costs (light blue denotes that both subpopulations have equal fitness, while costly plasmids are represented with darker tones of blue). As expected, the fraction of plasmid-bearing cells increases as the conjugation rate (γ) increments. The arrow shows the critical conjugation rate (γ^*) such that if $\gamma > \gamma^*$ the plasmid is positively selected (the plasmid fraction is larger than 50% at the end of the experiment). This numerical experiment shows that γ^* is positively correlated with plasmid cost and therefore maintenance of costly plasmids is observed only for large conjugation rates.

Supplementary Figure 10. Effect of conjugation and community complexity in plasmid population dynamics in the absence of variability of plasmid fitness effects.

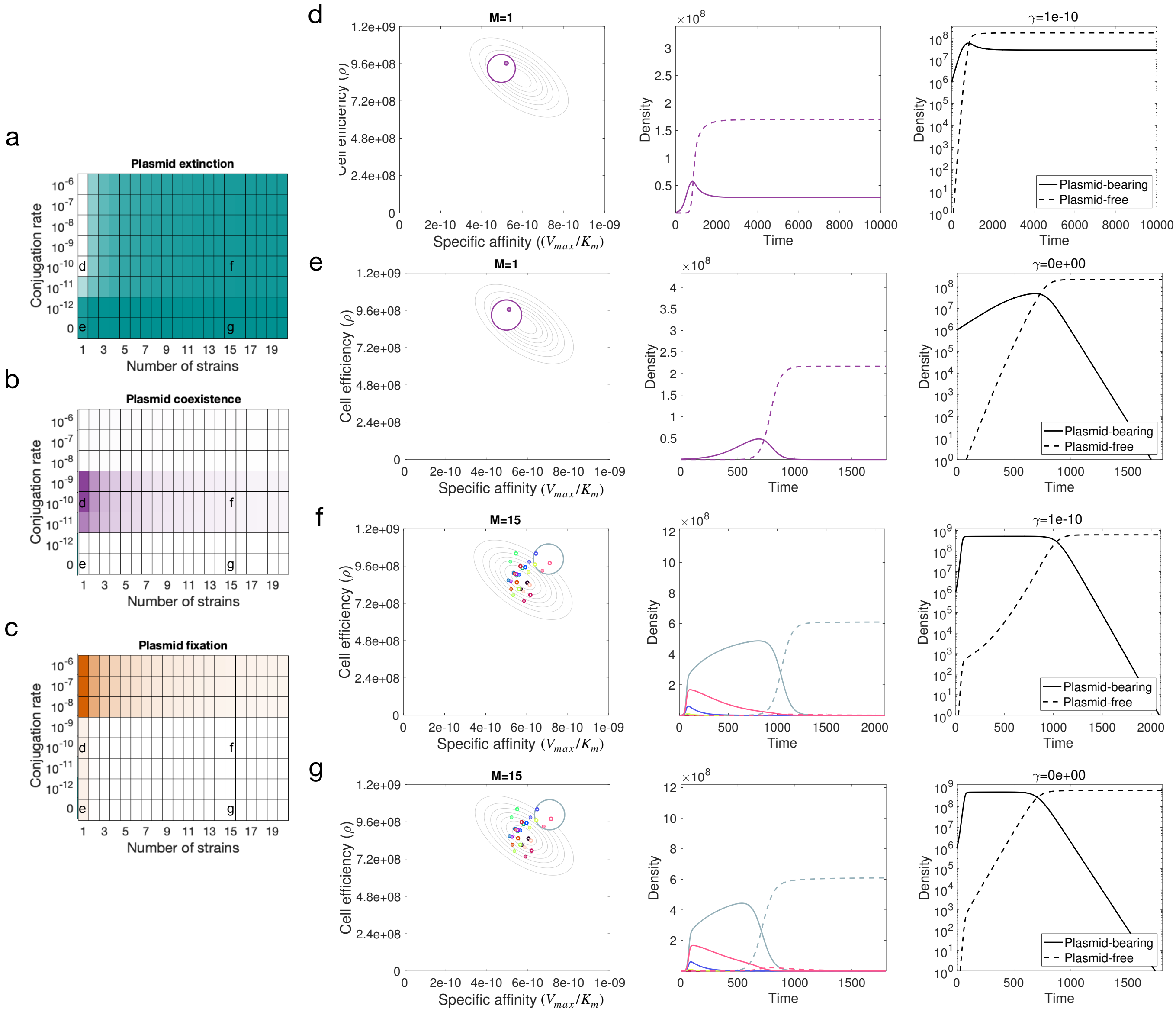

Numerical simulations of the population dynamics model performed over a range of conjugation rates and number of strains in the community. In this case, all strains exhibit a reduction of fitness when carrying the plasmid (mean plasmid fitness effects *w*= 0.985 and variance 0). The colour of each box in the grid corresponds to the percentage of $5{,}000$ random communities that exhibited: a) plasmid extinction (total plasmid frequency was below a threshold), b) plasmid-bearing and plasmid-free cells co-exist in the population, and c) every cell in the population carries the plasmid at the end of the experiment. (d-g) Example of relative abundances over time for a range of conjugation rates in a community composed of 1 (d,e) and 15 (f,g) strains, with segregation rate $\lambda = 1 \times 10^{-8}$ and conjugation rate $\gamma = 10^{-10}$ (d,f) or $\gamma = 0$ (e,g). The left-hand column illustrates the growth kinetic parameters for

each strain (empty circles denote plasmid-free cells and filled circles plasmid-bearing cells, with diameters proportional to their final relative abundances). Middle column shows the density of each subpopulation as a function of time (dotted lines denote plasmid-free strains and solid lines subpopulations carrying the plasmid). Right-hand column shows semilog plots with the total fraction of cells with and without plasmids (solid and dotted lines, respectively). As plasmid-bearing is associated with a fixed fitness cost, then the plasmid is only maintained in the population at high conjugation rates.

Supplementary Figure 11. Effect of conjugation and community complexity in plasmid population dynamics in the presence of variability of plasmid fitness effects.

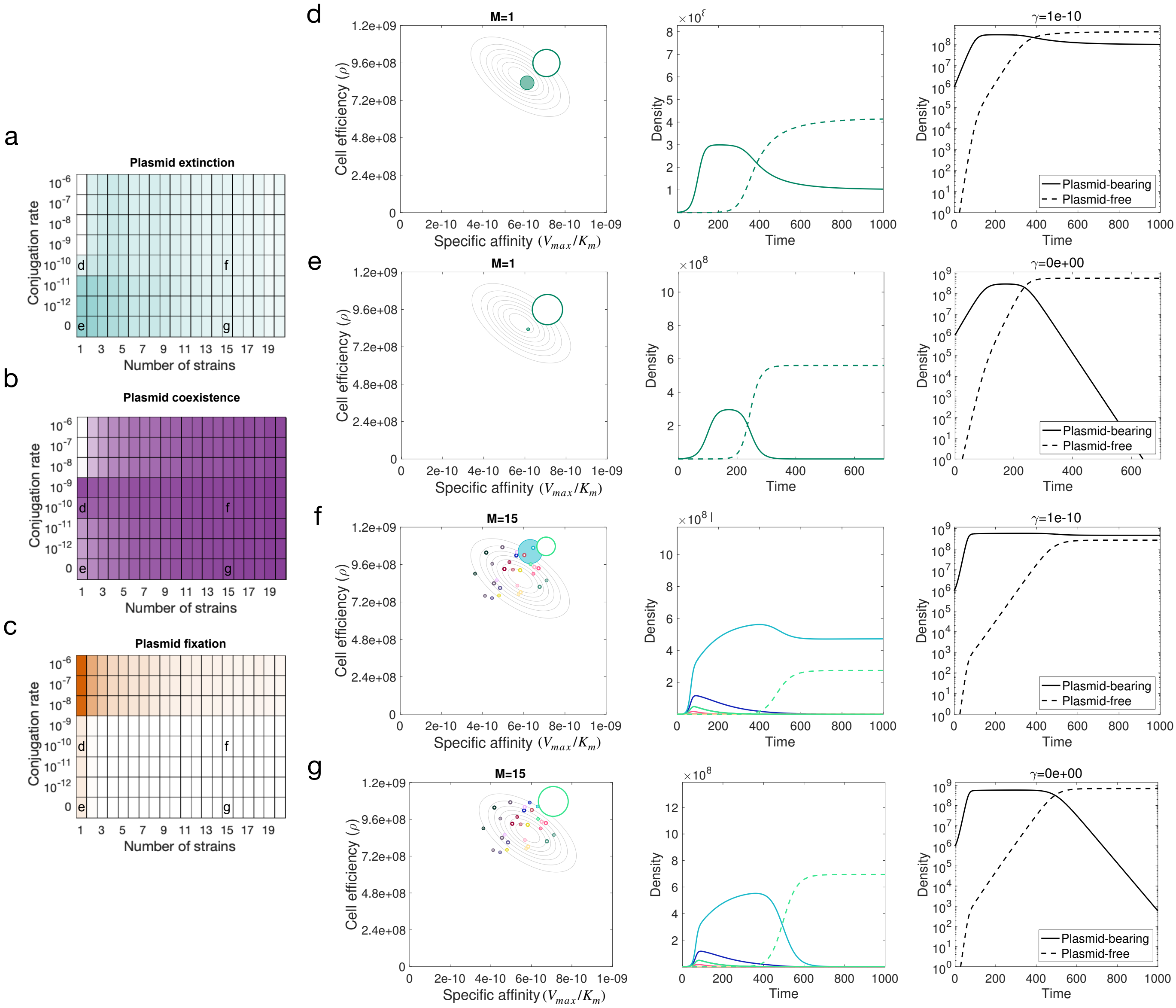

Numerical simulations of the population dynamics model performed over a range of conjugation rates and number of strains in the community. This case corresponds to a wide distribution of plasmid fitness effects (mean $w = 0.985$ and variance 0.007). The colour of each box in the grid corresponds to the percentage of 5,000 random communities that exhibited: a) plasmid extinction (total plasmid frequency was below a threshold), b) plasmid-bearing and plasmid-free cells co-exist in the population, and c) every cell in the population carries the plasmid at the end of the experiment. d-g) Example of relative abundances over time for a range of conjugation rates in a community composed of 1 (d,e) and 15 (f,g) strains, with segregation rate $\lambda = 1 \times 10^{-8}$ and conjugation rate $\gamma = 10^{-10}$ (d,f) or $\gamma = 0$ (e,g). The left-hand column illustrates the growth kinetic parameters for each strain (empty circles denote plasmid-free

cells and filled circles plasmid-bearing cells, with diameters proportional to their final relative abundances). Middle column shows the density of each subpopulation as a function of time (dotted lines denote plasmid-free strains and solid lines subpopulations carrying the plasmid). Right-hand column shows semilog plots with the total fraction of cells with and without plasmids (solid and dotted lines, respectively). Note how a large variability of fitness effects allows plasmids to persist, even at very low conjugation rates.

Supplementary Figure 12. Determination of different cells types in competition assays using flow cytometry.

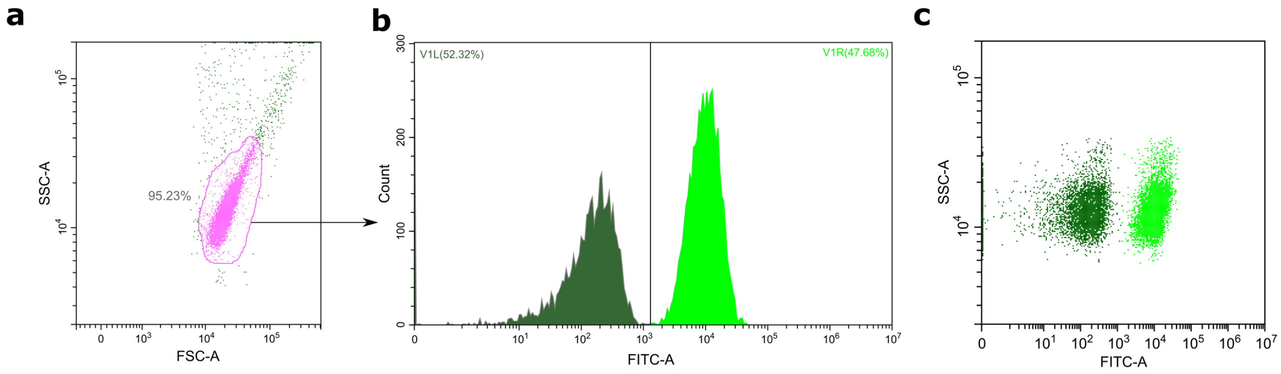

We used flow cytometry to differentiate between GFP-producing and -non-producing cells. (a) we used forward versus side scatter (FSC vs SSC) gating to identify bacterial cells in the sample. (b-c) GFP-producing (bright green) and -non-producing (dark green) cells were differentiated using the FITC-A (fluorescein isothiocyanate) channel, allowing us to measure the proportion of each competitor in the mix.

Supplementary Figure 13. Distribution of pBGC fitness effects.

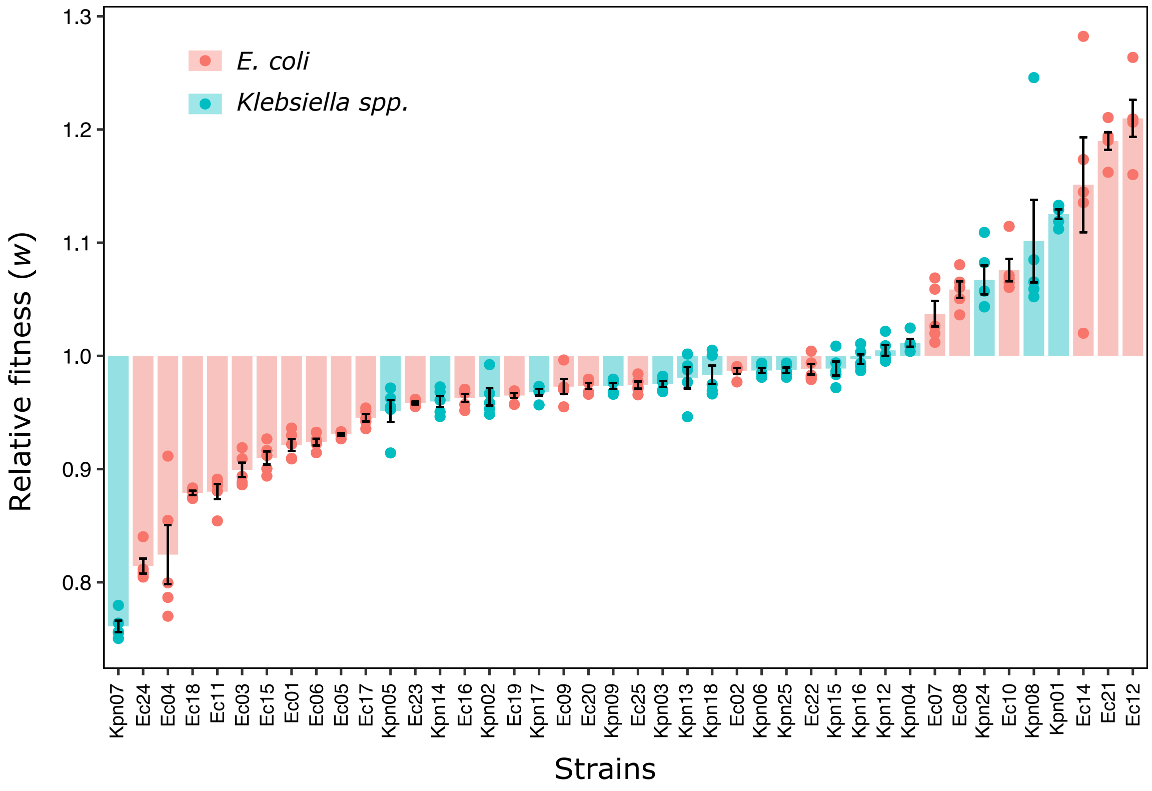

Relative fitness (*w*) of pBGC-carrying clones compared to plasmid-free clones, obtained from competition assays (n= 5 independent replicates; red, *E. coli* and blue, *Klebsiella* spp.). Values below 1 indicate a reduction in *w* and values above 1 indicate an increase in *w* due to pBGC acquisition. Bars represent the average of five independent experiments, with the individual data points overlaid as dot plots, and error bars represent the standard error of the mean. Note that the fitness effects of pBGC did not correlate with those form pOXA-48 (one-sided Pearson's product-moment correlation, R= 0.11, t= 0.66, df= 39, P= 0.51). Source data are provided as a Source Data file.

Supplementary Figure 14. Correlation between relative fitness values calculated in competitions vs. *E. coli* J53/pBGC or vs. isogenic bacteria with pBGC.

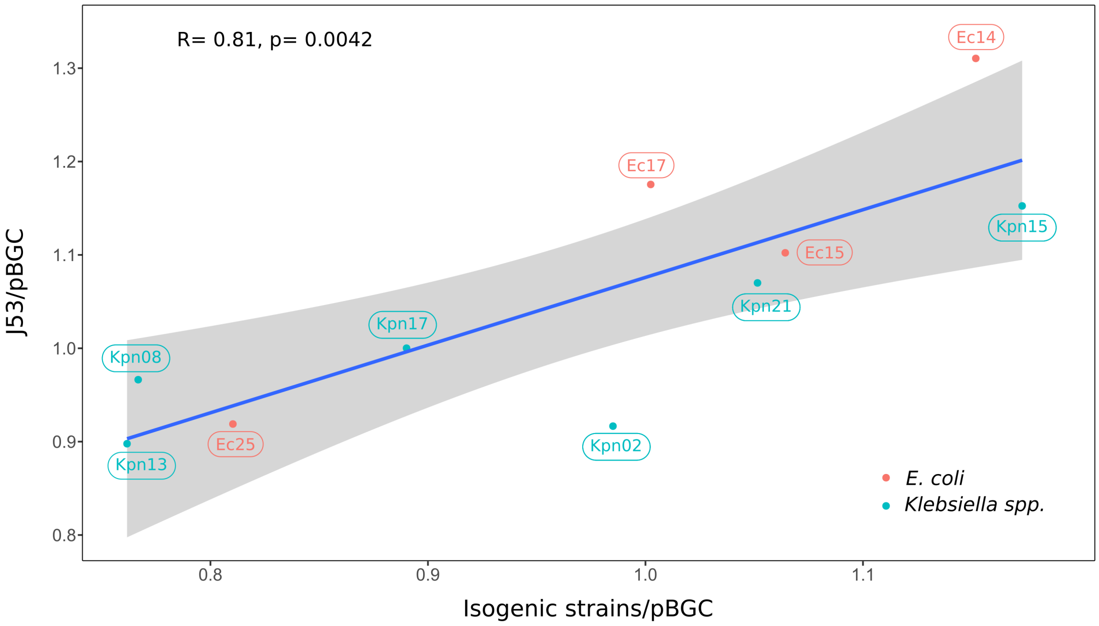

Correlation between relative fitness values obtained from competitions assays using pBGC-carrying isogenic isolates and pBGC-carrying *E.* coli J53 for ten different isolates (n=4 biologically independent *E. coli* and 6 biologically independent *Klebsiella* spp. strains). The blue line represents the linear regression and the grey shading represents 95% confidence intervals. Blue points correspond to *Klebsiella* spp. isolates and red points to *E. coli* isolates. Labels indicate isolates names. Pearson's product-moment correlation (*R*) and *p*-value are indicated (one-sided). Source data are provided as a Source Data file.

Supplementary Figure 15. Sampling the distribution of plasmid fitness effects.

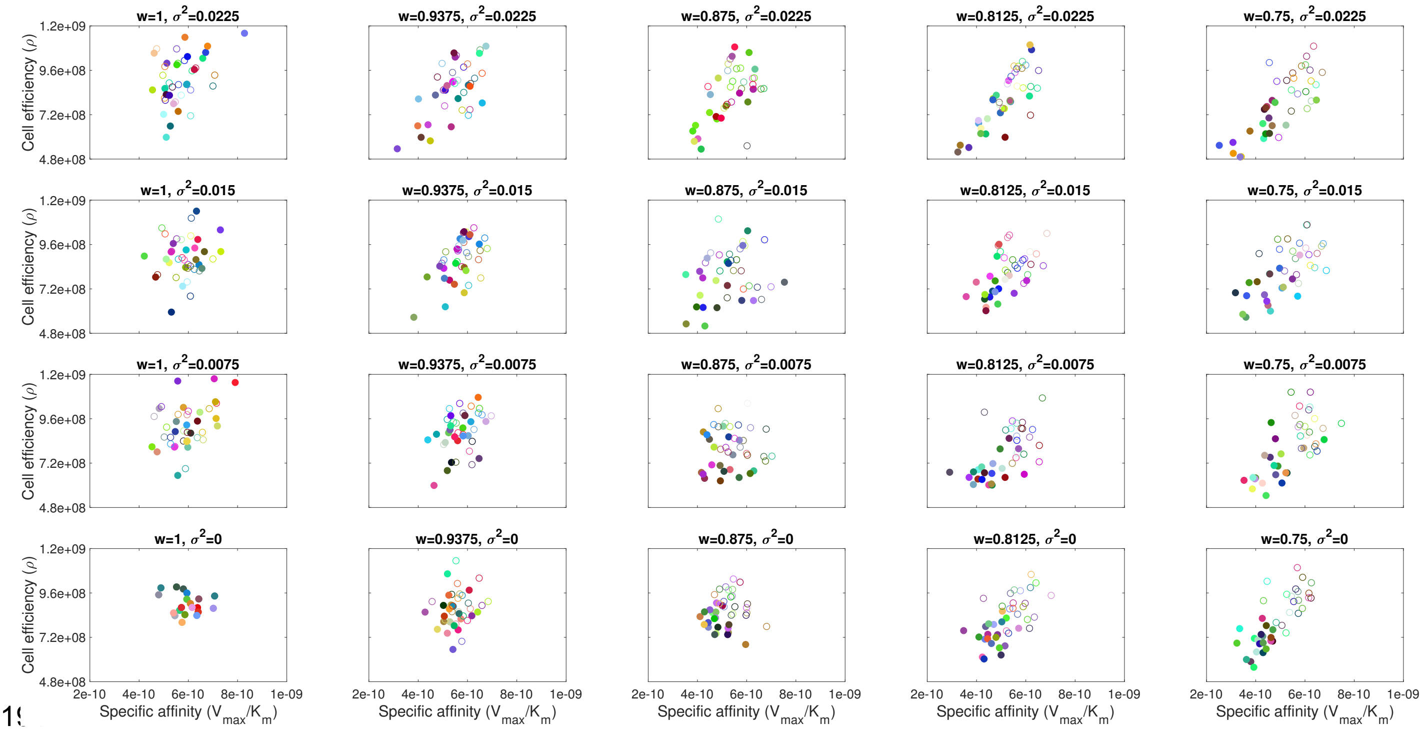

Growth kinetic parameter values for plasmid-bearing cells (filled circles) and their corresponding plasmid-free cells (same colour, empty circles), randomly sampled from a distribution of relative fitness of plasmid-carrying compared to plasmid-free with mean (*w*) and variance (σ^2). As expected, by decreasing *w*, the fitness difference between plasmid-free and plasmid-bearing is larger (here represented as filled circles clustering in the bottom-left part of the plot), while σ^2>0 increases the probability of existence of a plasmid-host association that produces a fitness benefit and, in consequence, increases the stability of the plasmid in the community.

Supplementary Table 1. Growth kinetic parameters of each strain obtained using the MCMC algorithm.

Strain	Wild-type		Transconjugant	
	V_{max}/K	ρ	V_{max}/K	ρ
Ec01	4.895×10^{-10}	9.844×10^{8}	5.283×10^{-10}	8.776×10^{8}
Ec02	7.603×10^{-10}	5.504×10^{8}	6.648×10^{-10}	6.786×10^{8}
Ec03	7.597×10^{-10}	8.900×10^{8}	6.538×10^{-10}	8.151×10^{8}
Ec04	8.373×10^{-10}	7.764×10^{8}	6.576×10^{-10}	7.702×10^{8}
Ec05	6.299×10^{-10}	7.727×10^{8}	7.831×10^{-10}	6.416×10^{8}
Ec06	6.852×10^{-10}	9.518×10^{8}	6.839×10^{-10}	6.567×10^{8}
Ec07	3.786×10^{-10}	1.040×10^{9}	4.154×10^{-10}	1.013×10^{9}
Ec08	5.304×10^{-10}	1.050×10^{9}	5.053×10^{-10}	9.900×10^{8}
Ec09	6.610×10^{-10}	9.195×10^{8}	7.311×10^{-10}	8.694×10^{8}
Ec10	5.660×10^{-10}	1.071×10^{9}	7.368×10^{-10}	8.472×10^{8}
Ec11	7.248×10^{-10}	5.064×10^{8}	7.248×10^{-10}	6.483×10^{8}
Ec12	5.200×10^{-10}	8.042×10^{8}	4.752×10^{-10}	8.016×10^{8}
Ec13	6.831×10^{-10}	8.528×10^{8}	7.711×10^{-10}	7.932×10^{8}
Ec14	6.515×10^{-10}	6.589×10^{8}	8.356×10^{-10}	7.500×10^{8}
Ec15	6.322×10^{-10}	9.106×10^{8}	6.998×10^{-10}	8.528×10^{8}
Ec16	4.908×10^{-10}	1.122×10^{9}	5.550×10^{-10}	1.040×10^{9}
Ec17	6.089×10^{-10}	8.298×10^{8}	6.366×10^{-10}	8.647×10^{8}
Ec18	6.655×10^{-10}	8.963×10^{8}	6.939×10^{-10}	6.250×10^{8}
Ec19	6.080×10^{-10}	9.812×10^{8}	6.200×10^{-10}	9.424×10^{8}
Ec20	5.033×10^{-10}	1.024×10^{9}	5.296×10^{-10}	9.133×10^{8}
Ec21	5.546×10^{-10}	1.021×10^{9}	4.897×10^{-10}	8.386×10^{8}
Ec22	6.148×10^{-10}	9.736×10^{8}	6.619×10^{-10}	9.886×10^{8}
Ec23	5.443×10^{-10}	9.635×10^{8}	5.186×10^{-10}	9.094×10^{8}
Ec24	6.010×10^{-10}	1.008×10^{9}	4.808×10^{-10}	9.768×10^{8}
Ec25	6.784×10^{-10}	9.237×10^{8}	5.465×10^{-10}	1.004×10^{9}
Kpn01	4.841×10^{-10}	8.746×10^{8}	3.652×10^{-10}	8.673×10^{8}
Kpn02	6.638×10^{-10}	7.905×10^{8}	6.352×10^{-10}	7.952×10^{8}
Kpn03	4.802×10^{-10}	9.639×10^{8}	3.686×10^{-10}	9.975×10^{8}
Kpn04	4.844×10^{-10}	9.288×10^{8}	4.303×10^{-10}	8.936×10^{8}
Kpn05	5.151×10^{-10}	9.303×10^{8}	5.155×10^{-10}	8.513×10^{8}
Kpn06	5.240×10^{-10}	8.952×10^{8}	5.664×10^{-10}	7.742×10^{8}
Kpn07	4.498×10^{-10}	8.682×10^{8}	3.785×10^{-10}	9.458×10^{8}
Kpn08	7.163×10^{-10}	8.211×10^{8}	4.334×10^{-10}	7.188×10^{8}
Kpn09	6.207×10^{-10}	8.064×10^{8}	7.302×10^{-10}	7.772×10^{8}
Kpn10	4.833×10^{-10}	7.990×10^{8}	4.467×10^{-10}	7.967×10^{8}
Kpn11	5.380×10^{-10}	9.710×10^{8}	5.620×10^{-10}	8.490×10^{8}
Kpn12	5.620×10^{-10}	8.490×10^{8}	5.620×10^{-10}	8.490×10^{8}

Kpn13	7.086×10^{-10}	9.195×10^{8}	7.364×10^{-10}	7.206×10^{8}
Kpn14	4.162×10^{-10}	8.994×10^{8}	5.828×10^{-10}	7.306×10^{8}
Kpn15	6.243×10^{-10}	8.854×10^{8}	7.489×10^{-10}	8.917×10^{8}
Kpn16	5.386×10^{-10}	8.523×10^{8}	4.359×10^{-10}	8.465×10^{8}
Kpn17	4.144×10^{-10}	1.037×10^{9}	4.002×10^{-10}	9.786×10^{8}
Kpn18	6.906×10^{-10}	7.173×10^{8}	4.977×10^{-10}	9.954×10^{8}
Kpn19	5.578×10^{-10}	8.319×10^{8}	6.083×10^{-10}	7.739×10^{8}
Kpn20	4.857×10^{-10}	1.022×10^{9}	4.287×10^{-10}	1.046×10^{9}
Kpn21	4.674×10^{-10}	1.077×10^{9}	5.487×10^{-10}	9.948×10^{8}
Kpn22	4.708×10^{-10}	1.006×10^{9}	5.224×10^{-10}	1.006×10^{9}
Kpn23	7.897×10^{-10}	6.709×10^{8}	7.201×10^{-10}	6.328×10^{8}
Kpn24	5.736×10^{-10}	7.568×10^{8}	8.670×10^{-10}	6.601×10^{8}
Kpn25	4.873×10^{-10}	9.630×10^{8}	4.255×10^{-10}	9.896×10^{8}

Supplementary Table 2. Primers used in this study.

Primers		
**Name**	**Sequence 5'->3'**	**Use**
Oxa-48 Fw	TTGGTGGCATCGATTATCGG	Amplification of *bla_{TEM1} gene*
Oxa-48 Rv	GAGCACTTCTTTTGTGATGGC	
IncL Fw	CGGAACCGACATGTGCCTACT	Amplification of *repC* gene
IncL Rv	GAACTCCGGCGAAAGACCTTC	
pBGC Fw	CGTTGATCGGCACGTAAG	Amplification of pBGC backbone for Gibson cloning (see Supplementary Figure 2)
pBGC Rv	GCTGTCTAGACTATTTGTATAGTTCATCCATGC	
GFP-Term Fw	atacaaatagtctagacagcGGGAATCCTGCTCTGCGAG[1]	
GFP-Term Rv	ctcttacgtgccgatcaacgGGGTTATTGTCTCATGAGCGG[1]	
pBGC_Seq1_Fw	AGTTAAAAGGTATTGATTTTAA	pBGC sequencing (3503-3524)[2]
pBGC_Seq1_Rv	GCCACATCTTGCGAATA	pBGC sequencing (464-480)[2]
pBGC_Seq2_Fw	ATAAGATCACTACCGGGC	pBGC sequencing (44-61)[2]
pBGC_Seq2_Rv	ACCCGACAGGACTATAAAGATA	pBGC sequencing (1285-1306)[2]
pBGC_Seq3_Fw	GAGGTAACTGGCTTGGAGG	pBGC sequencing (931-949)[2]
pBGC_Seq3_Rv	GTCGCGTCTGTCACATCT	pBGC sequencing (2124-2141)[2]
pBGC_Seq4_Fw	GTTTCCCGACTGGAAAGC	pBGC sequencing (1703-1720)[2]
pBGC_Seq4_Rv	CTTTGGTCCCGCTTTGTTAC	pBGC sequencing (2894-2913)[2]
pBGC_Seq5_Fw	GTCGGTCGATAAAAAAATCGAG	pBGC sequencing (2650-2671)[2]
pBGC_Seq5_Rv	ATGTGGTCTCTCTTTTCGTTGG	pBGC sequencing (3764-2671)[2]

1. Lower case nucleotides correspond with the added cohesive ends.

2. Numbers correspond with primers binding sites in pBGC (according to sequence available with GenBank Accession Number MT702881).

Variability of plasmid fitness effects contributes to plasmid persistence in bacterial communities

Aida Alonso-del Valle, Ricardo León-Sampedro, Jerónimo Rodríguez-Beltrán, Javier DelaFuente, Marta Hernández-García, Patricia Ruiz-Garbajosa, Rafael Cantón, Rafael Peña-Miller*, Álvaro San Millán*

Supplementary File 1

Diagnostic plots of the MCMC algorithm for all strains used in this study.

(a) Traces of chains for parameters V_{max}/K_m (below) and ρ (above) obtained by fitting a simple Monod model to growth curve data using a Metropolis-Hastings Markov chain Monte Carlo method (MCMC) after performing 1×10^7 iterations with a burn-in period of 20% and a thinning of 100 iterations. Convergence of chains was determined by visual inspection and by obtaining values close to one when computing the Gelman-Rubin diagnostic statistics. (b) 2-dimensional posterior distributions obtained after fitting each strain independently, with mean represented by a solid dot (wild-type: blue, transconjugant: red). (c-d) Growth kinetic parameters estimated for each strain using the MCMC algorithm with different prior distributions: uniform, lognormal, beta and gamma (one-sided ANOVA, $P>0.05$, H_0: there are significant differences in estimates when considering different priors). Error bars represent standard deviation over the mean (computed for the last $n=5 \times 10^4$ elements in the Markov chain). (e) Numerical solutions of the model using 100 parameters selected randomly from the posterior distribution estimated by the MCMC algorithm using a uniform prior distribution. Initial conditions of the differential equations were determined from the optical density observed at $t=0$. (f-g) Identifiability of parameters was determined by artificially generating k clones of the data and using the Metropolis-Hastings MCMC algorithm to estimate a likelihood function that corresponds to the original function raised to the k-th power. Note how, as the number of clones increases, the variance of the posterior distribution converges to the mean of the maximum likelihood of the corresponding parameter, indicating estimability of the parameters (one-sided ANOVA $P>0.05$, H_0: there are significant differences in estimates when considering different number of data clones).

Escherichia coli

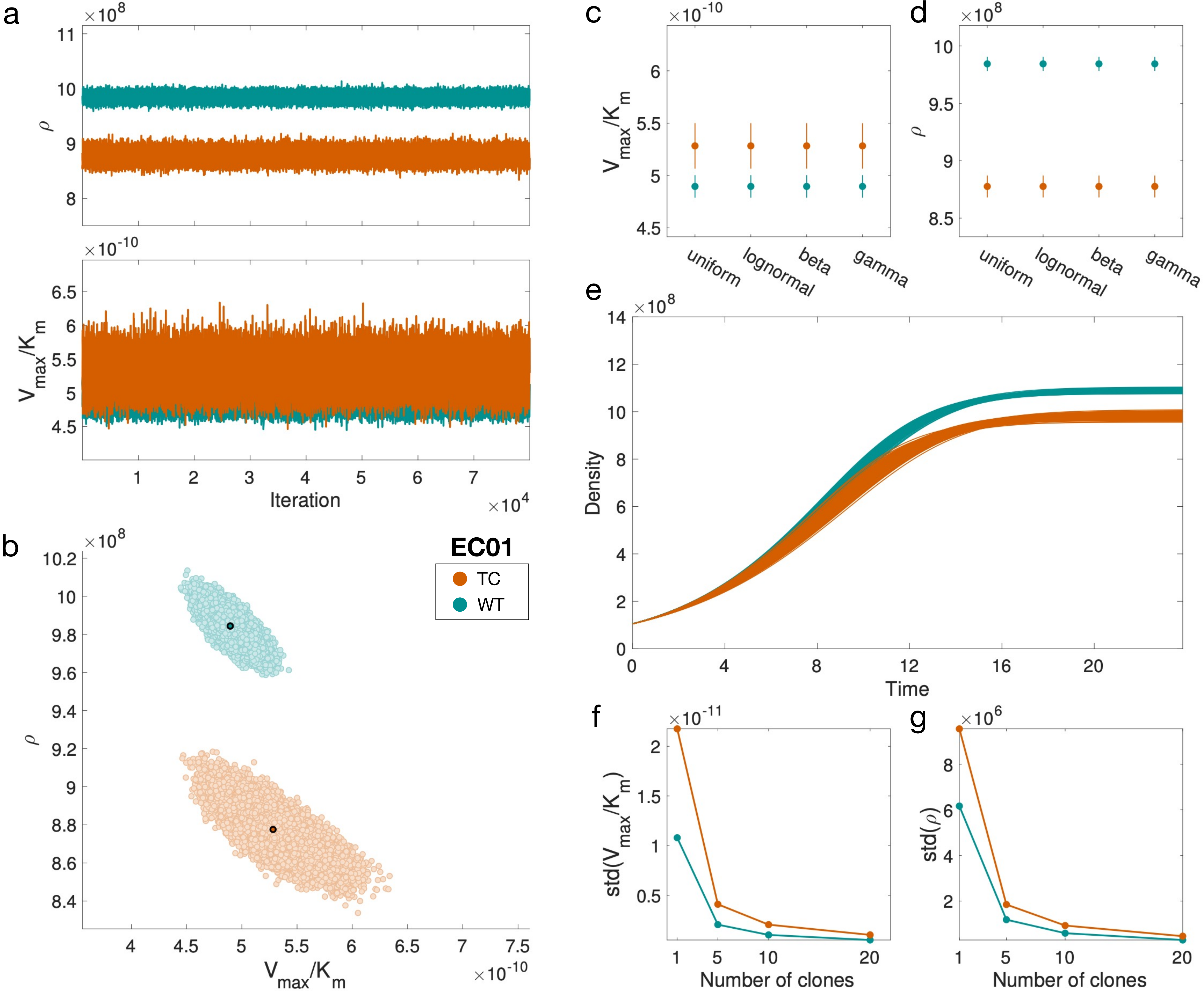

a
b
c
d
e
f
g
EC01
TC
WT
Iteration
Density
Time
Number of clones
uniform
lognormal
beta
gamma

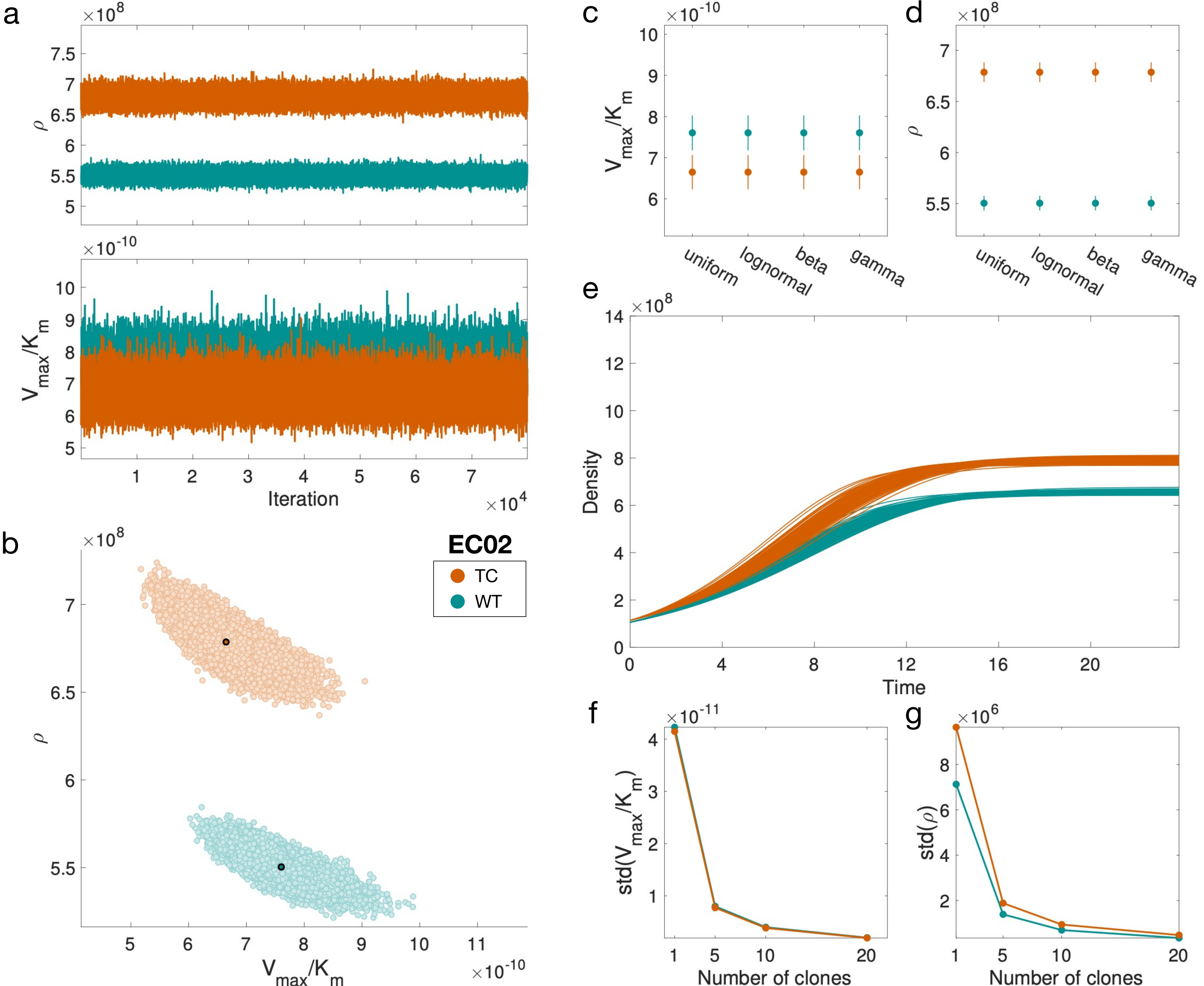
a
ρ
×10^8
Vmax/Km
×10^-10
Iteration
×10^4
b
EC02
TC
WT
ρ
×10^8
Vmax/Km
×10^-10
c
Vmax/Km
×10^-10
uniform
lognormal
beta
gamma
d
ρ
×10^8
uniform
lognormal
beta
gamma
e
Density
×10^8
Time
f
std(Vmax/Km)
×10^-11
Number of clones
g
std(ρ)
×10^6
Number of clones

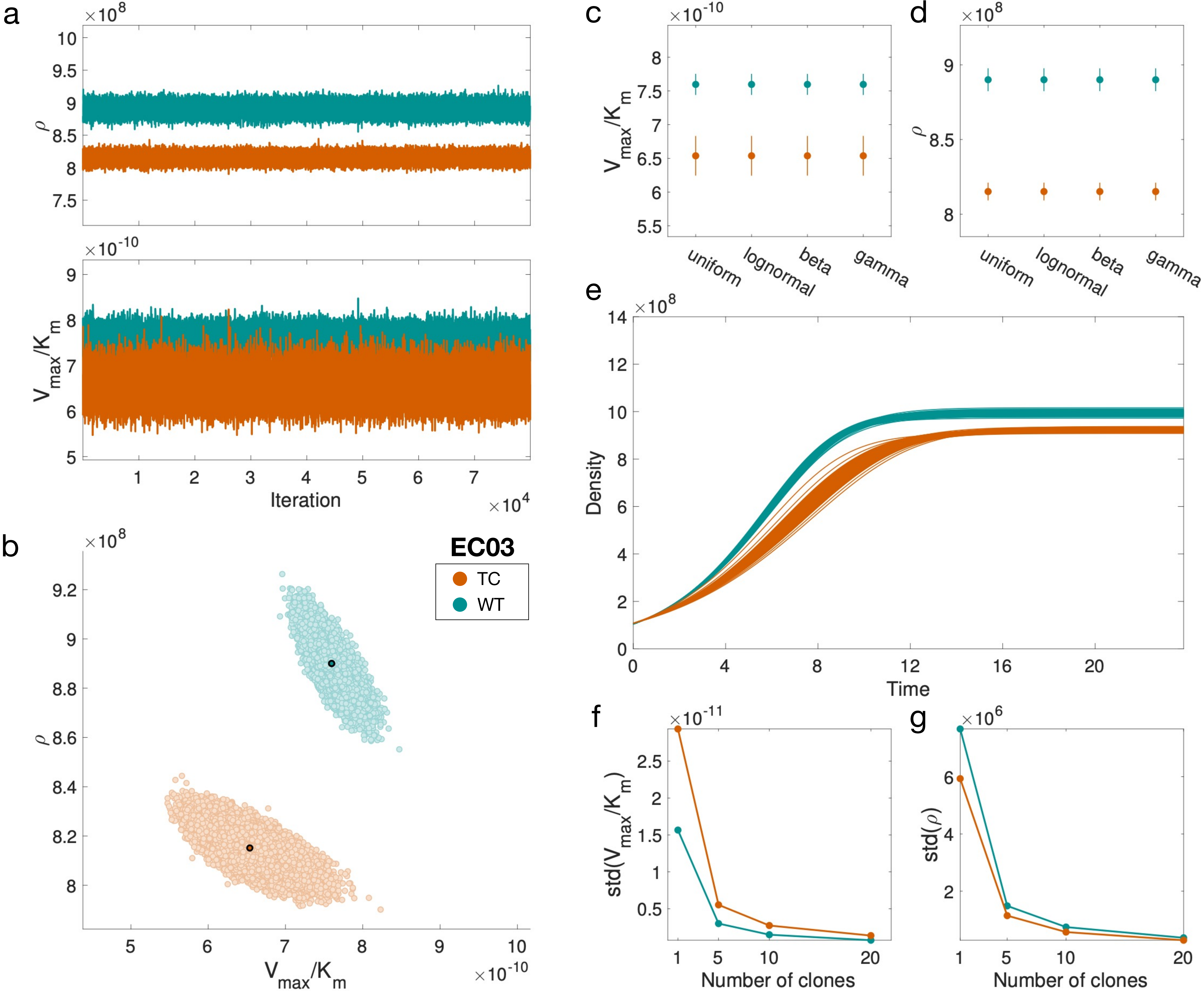

a
b
c
d
e
f
g
EC03
TC
WT
ρ
V_{max}/K_m
Iteration
Density
Time
$std(V_{max}/K_m)$
std(ρ)
Number of clones
uniform
lognormal
beta
gamma

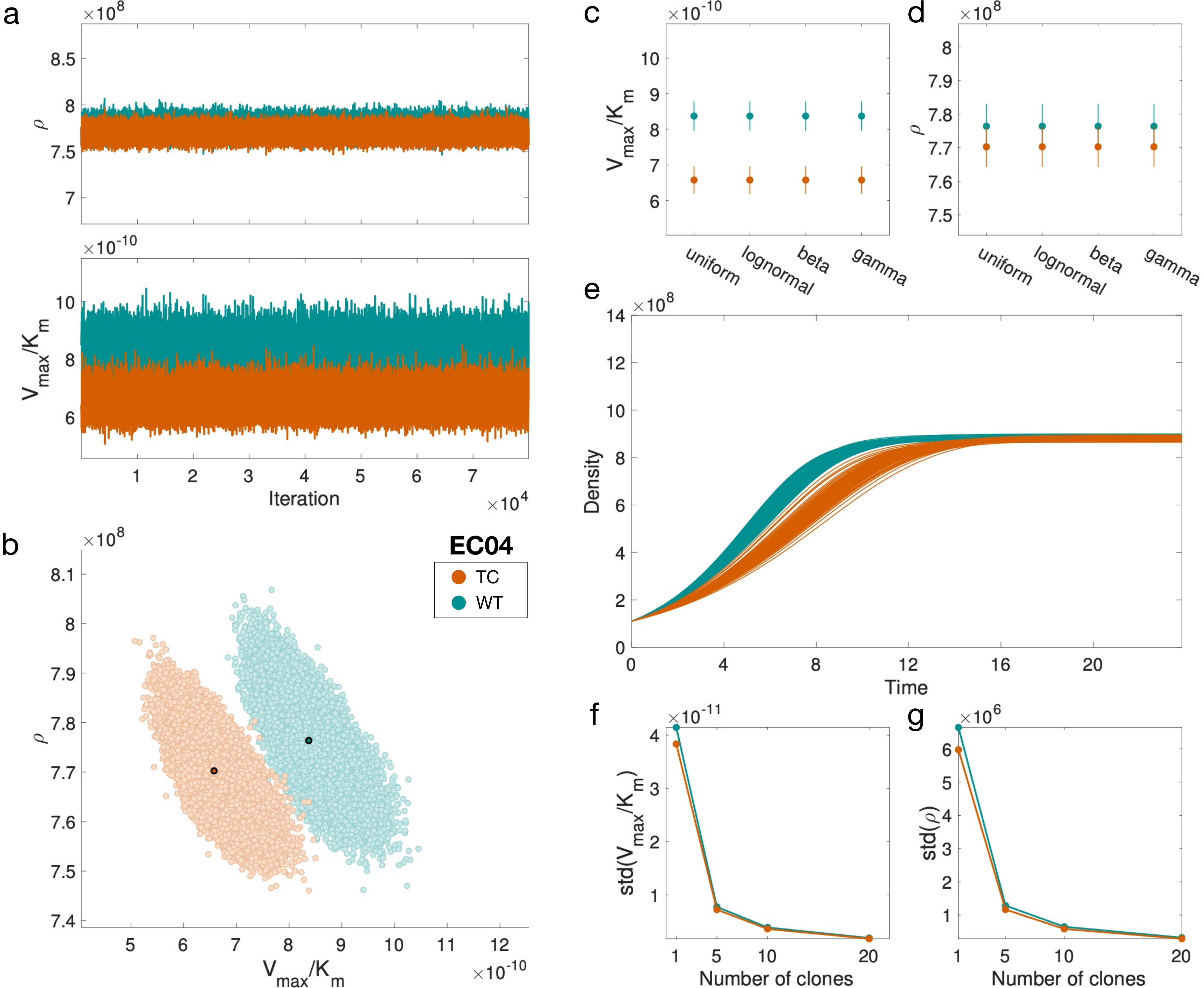

a
ρ
×10^8
Iteration
×10^4
V_max/K_m
×10^-10
b
EC04
TC
WT
ρ
×10^8
V_max/K_m
×10^-10
c
V_max/K_m
×10^-10
uniform
lognormal
beta
gamma
d
ρ
×10^8
uniform
lognormal
beta
gamma
e
Density
×10^8
Time
f
std(V_max/K_m)
×10^-11
Number of clones
g
std(ρ)
×10^6
Number of clones

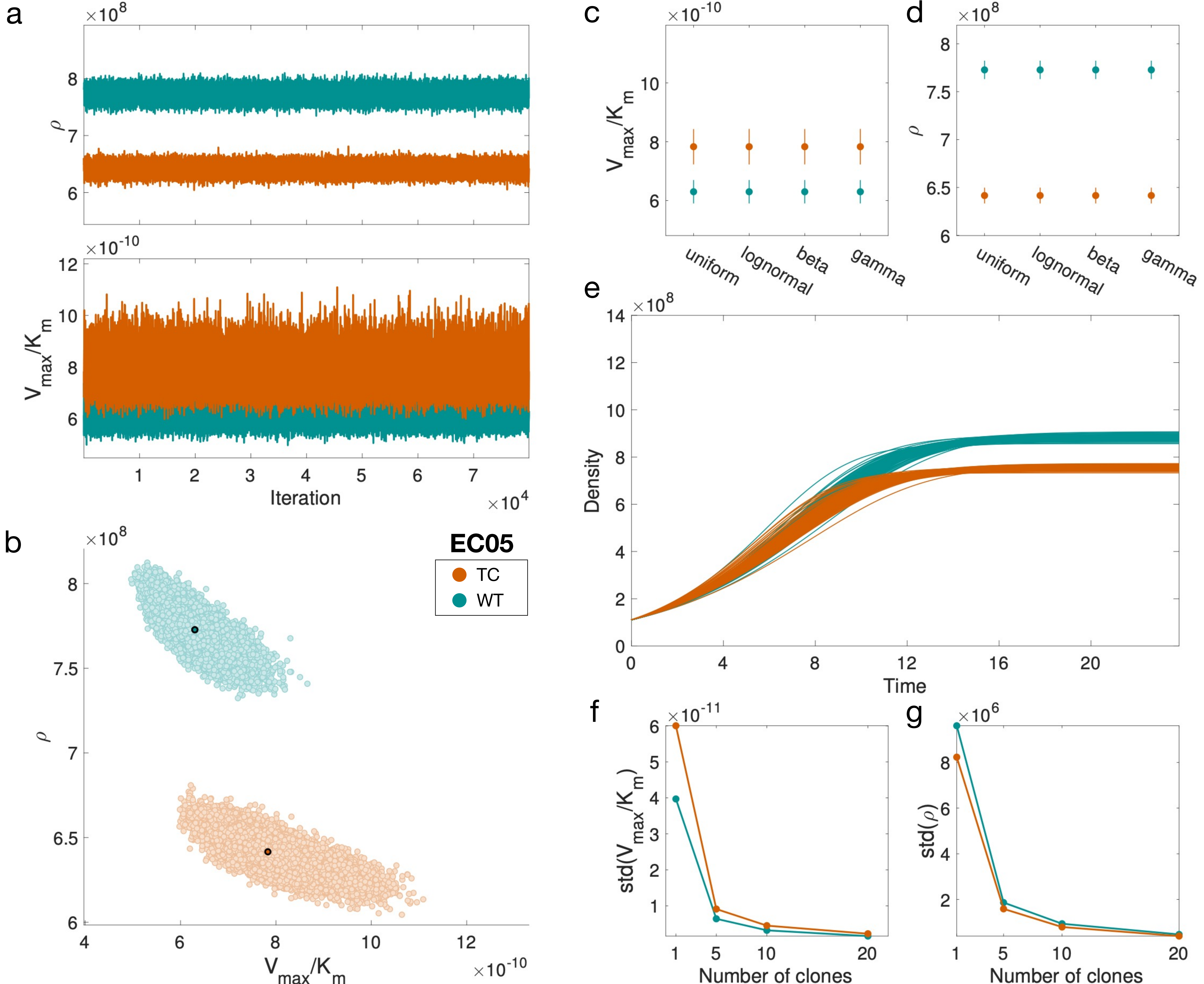
a
×10^8
ρ
8
7
6
×10^-10
V_max/K_m
12
10
8
6
1 2 3 4 5 6 7
Iteration
×10^4
b
×10^8
EC05
TC
WT
ρ
8
7.5
7
6.5
6
4 6 8 10 12
V_max/K_m
×10^-10
c
×10^-10
V_max/K_m
10
8
6
uniform
lognormal
beta
gamma
d
×10^8
ρ
8
7.5
7
6.5
6
uniform
lognormal
beta
gamma
e
×10^8
Density
14
12
10
8
6
4
2
0
0 4 8 12 16 20
Time
f
×10^-11
std(V_max/K_m)
6
5
4
3
2
1
1 5 10 20
Number of clones
g
×10^6
std(ρ)
8
6
4
2
1 5 10 20
Number of clones

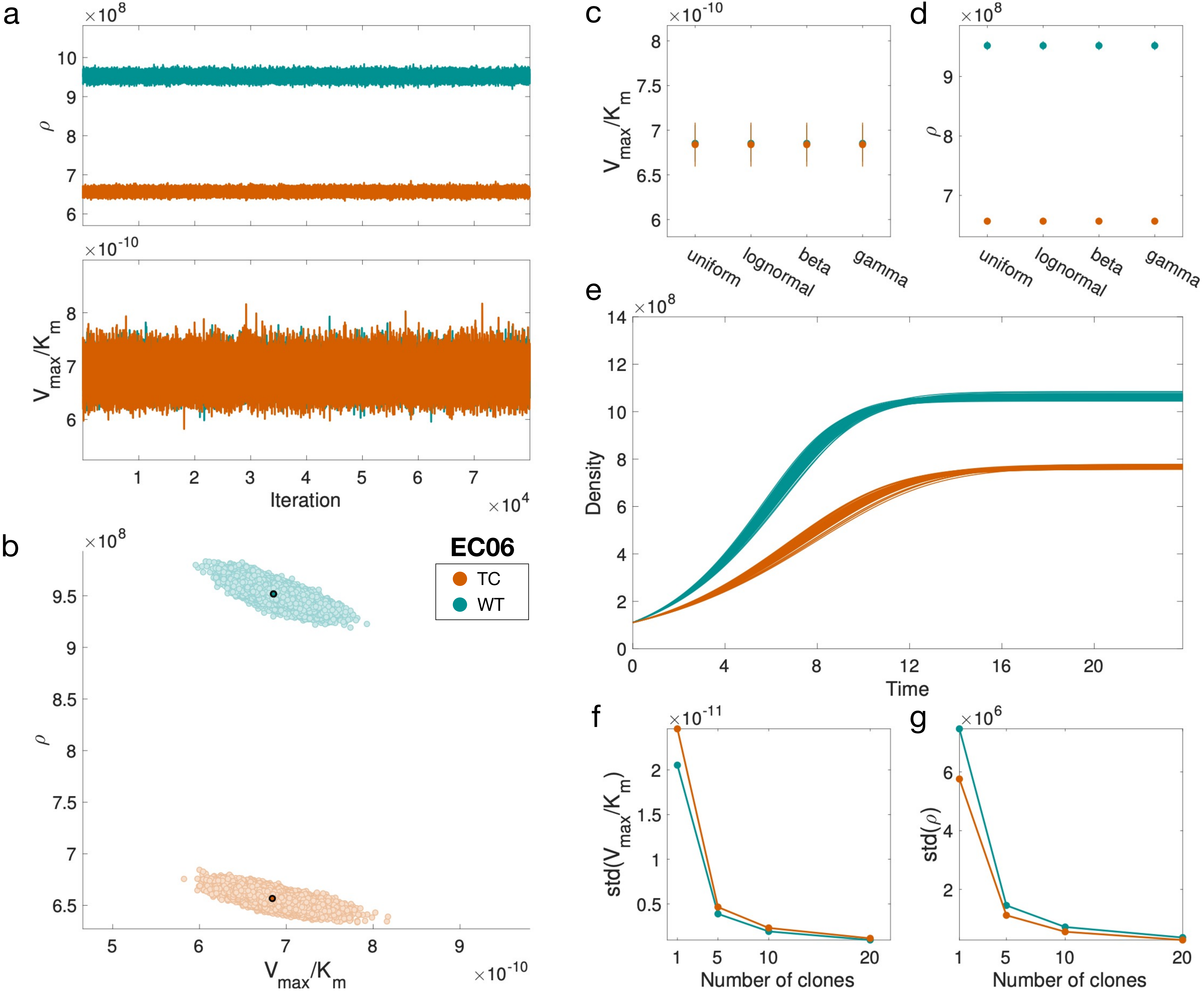
a
b
c
d
e
f
g
EC06
TC
WT
Iteration
Time
Density
Number of clones
uniform
lognormal
beta
gamma

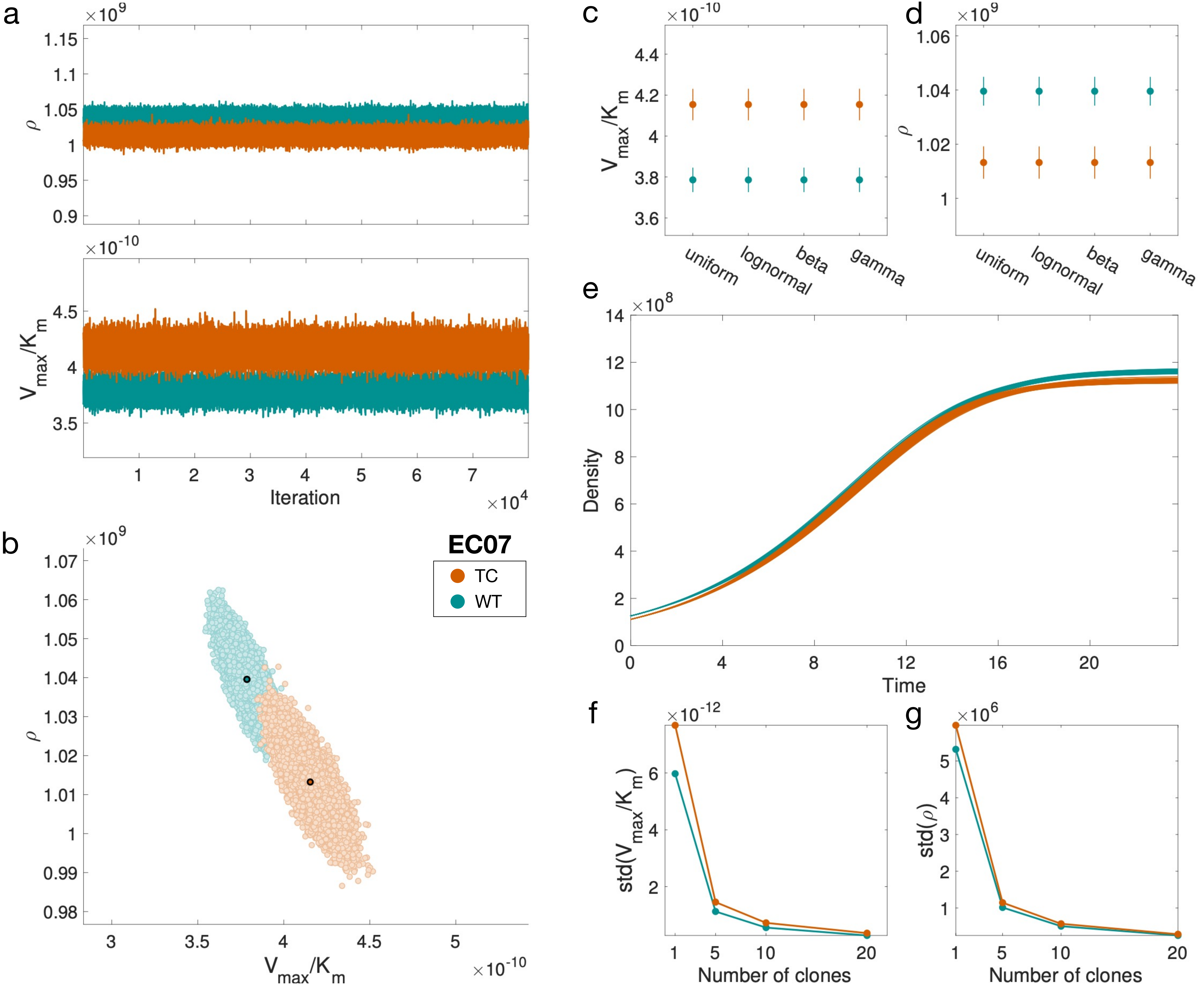
a
b
c
d
e
f
g
EC07
TC
WT
Iteration
$\times 10^4$
ρ
V_{max}/K_m
uniform
lognormal
beta
gamma
Density
Time
std(V_{max}/K_m)
std(ρ)
Number of clones

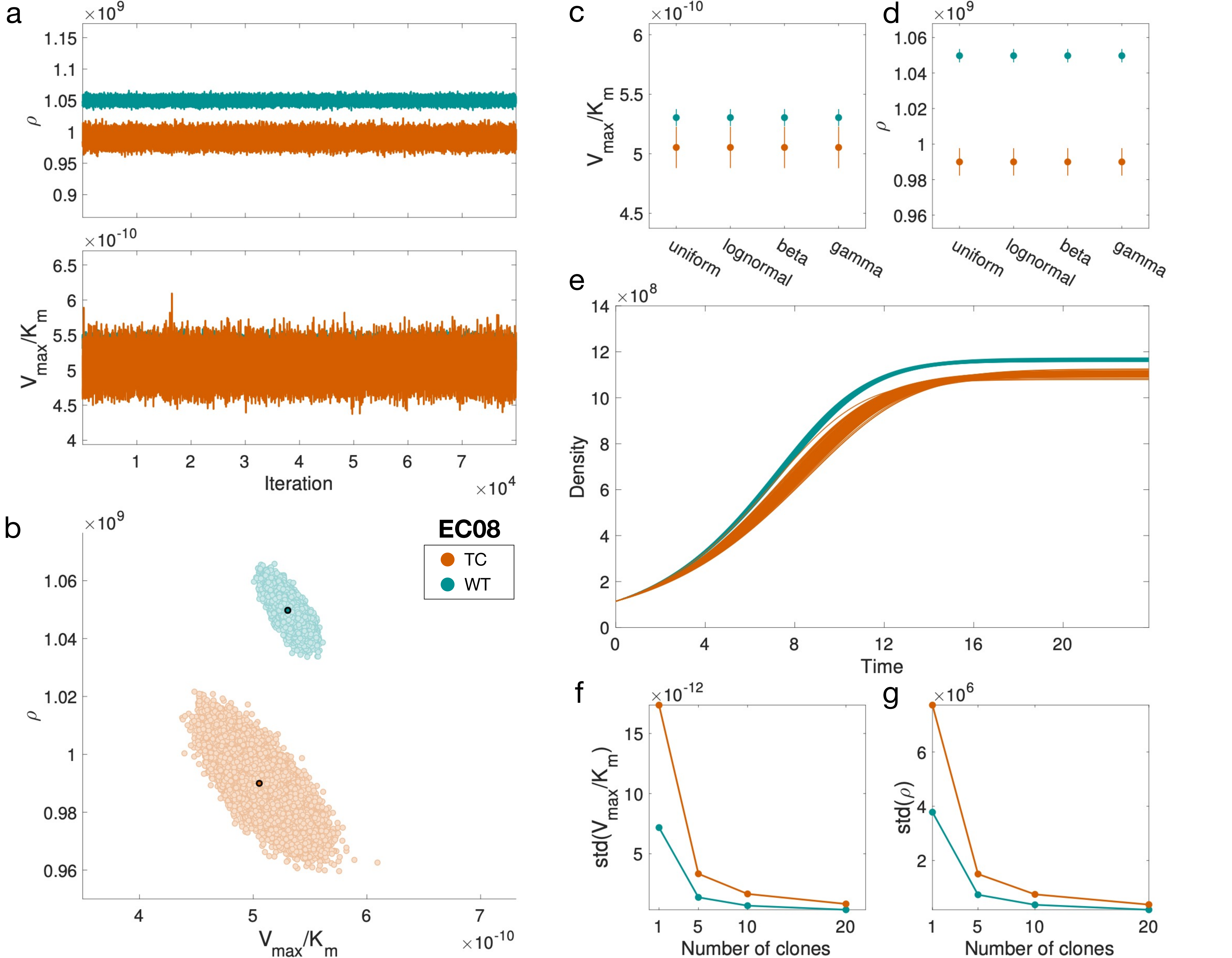

a
b
c
d
e
f
g
EC08
TC
WT
Iteration
Time
Density
Number of clones
V_{max}/K_m
ρ
std(V_{max}/K_m)
std(ρ)
uniform
lognormal
beta
gamma

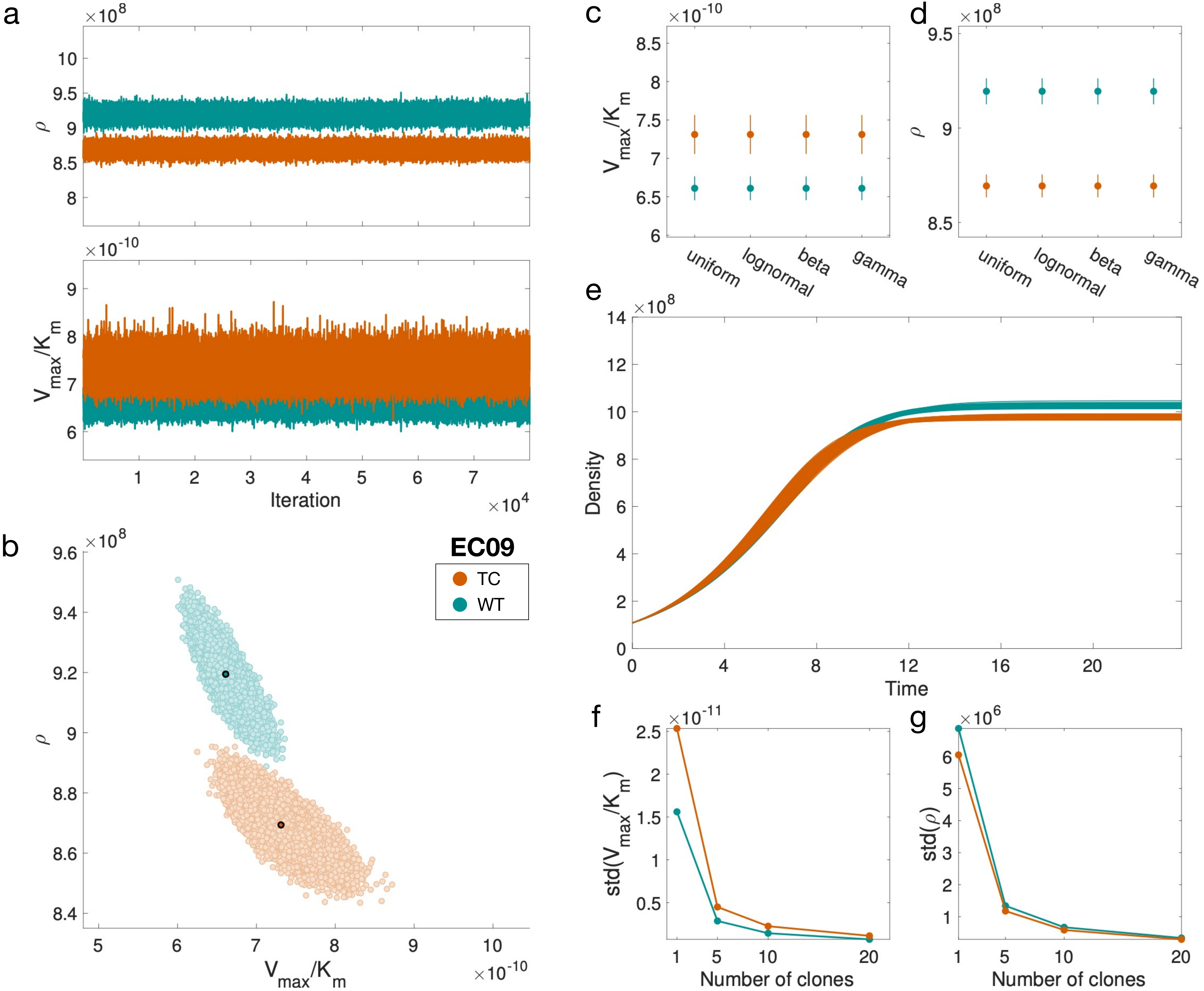

a
b
c
d
e
f
g
EC09
TC
WT
ρ
V_{max}/K_m
Iteration
Density
Time
Number of clones
std(V_{max}/K_m)
std(ρ)
uniform
lognormal
beta
gamma

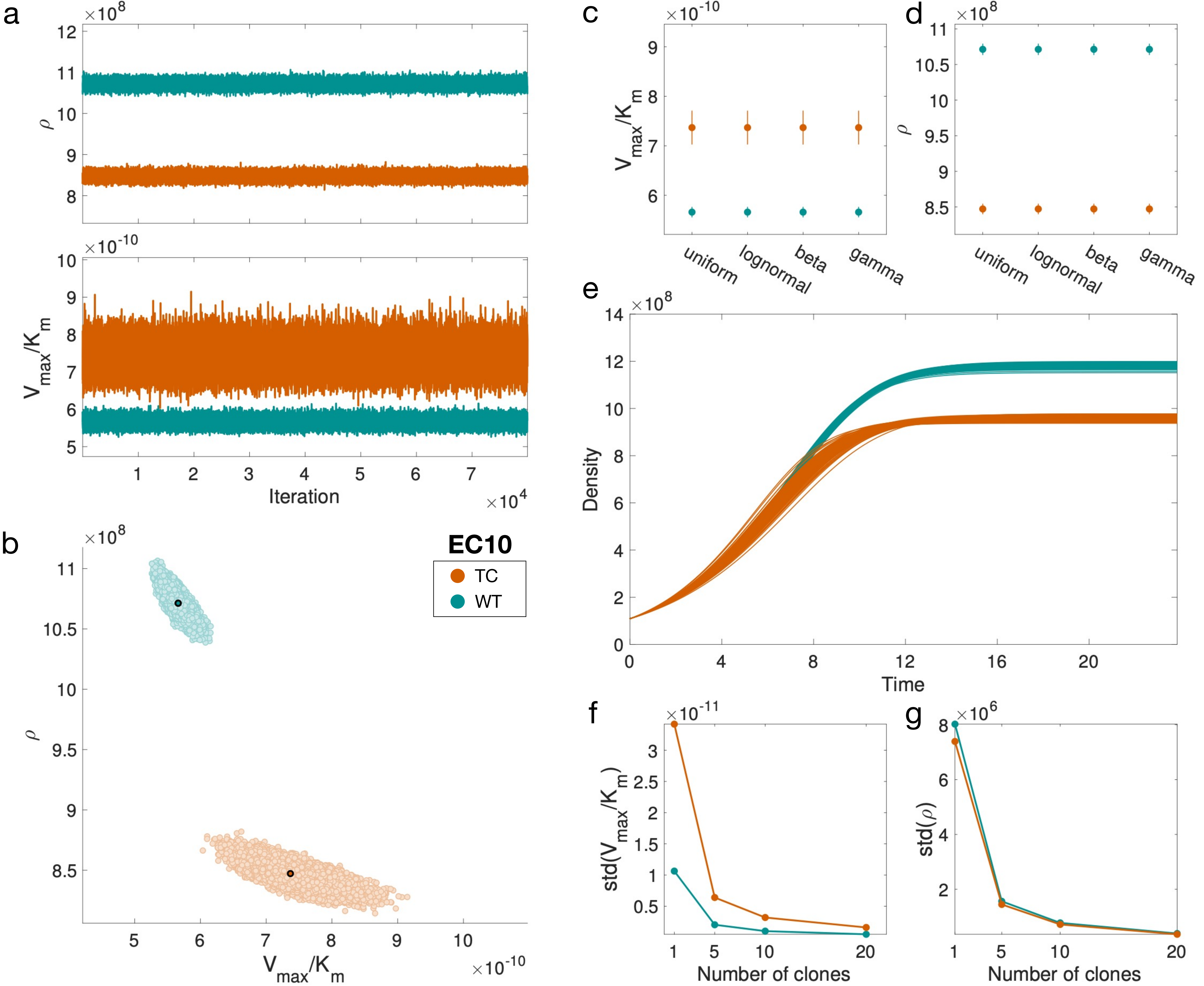

a
b
c
d
e
f
g
EC10
TC
WT
Iteration
Time
Density
Number of clones
uniform
lognormal
beta
gamma

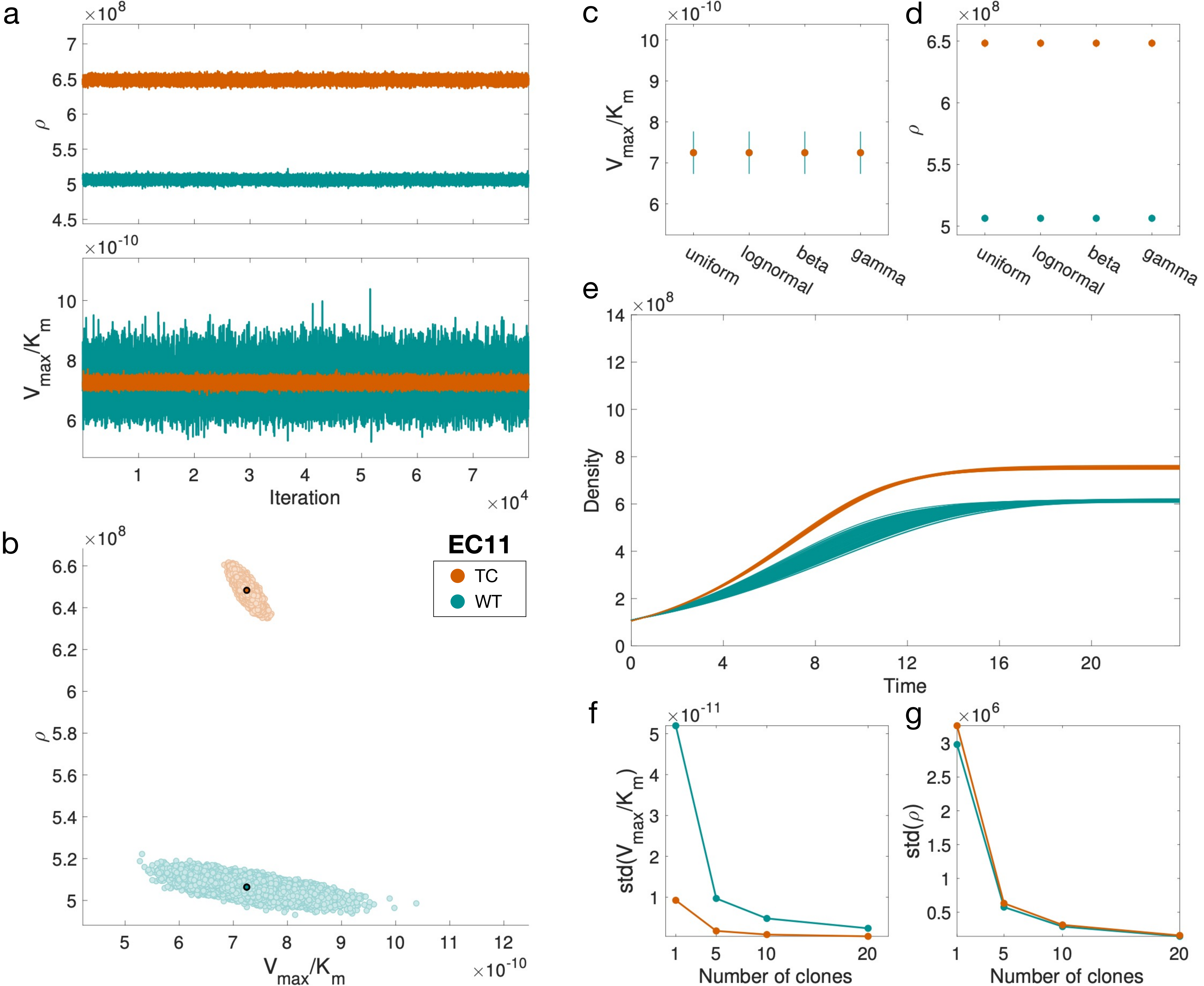

a
×10^8
ρ
7
6.5
6
5.5
5
4.5
×10^-10
V_max/K_m
10
8
6
1
2
3
4
5
6
7
Iteration
×10^4
b
×10^8
EC11
TC
WT
6.6
6.4
6.2
6
ρ 5.8
5.6
5.4
5.2
5
5
6
7
8
9
10
11
12
V_max/K_m
×10^-10
c
×10^-10
V_max/K_m
10
9
8
7
6
uniform
lognormal
beta
gamma
d
×10^8
ρ
6.5
6
5.5
5
uniform
lognormal
beta
gamma
e
×10^8
Density
14
12
10
8
6
4
2
0
0
4
8
12
16
20
Time
f
×10^-11
std(V_max/K_m)
5
4
3
2
1
1
5
10
20
Number of clones
g
×10^6
std(ρ)
3
2.5
2
1.5
1
0.5
1
5
10
20
Number of clones

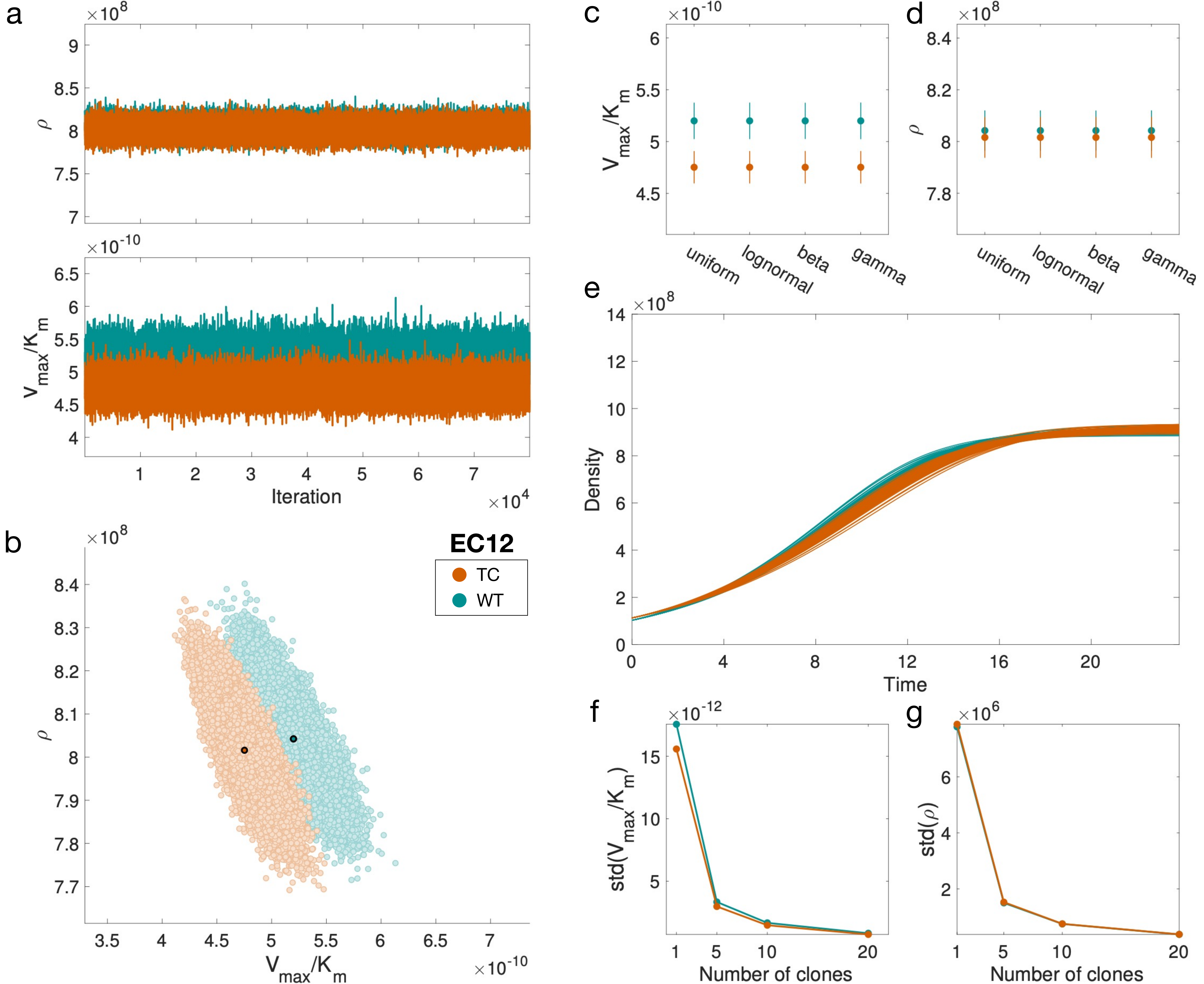

a
ρ
Iteration
$\times 10^4$
V_{max}/K_m
b
EC12
TC
WT
c
d
uniform
lognormal
beta
gamma
e
Density
Time
f
$std(V_{max}/K_m)$
Number of clones
g
std(ρ)
Number of clones

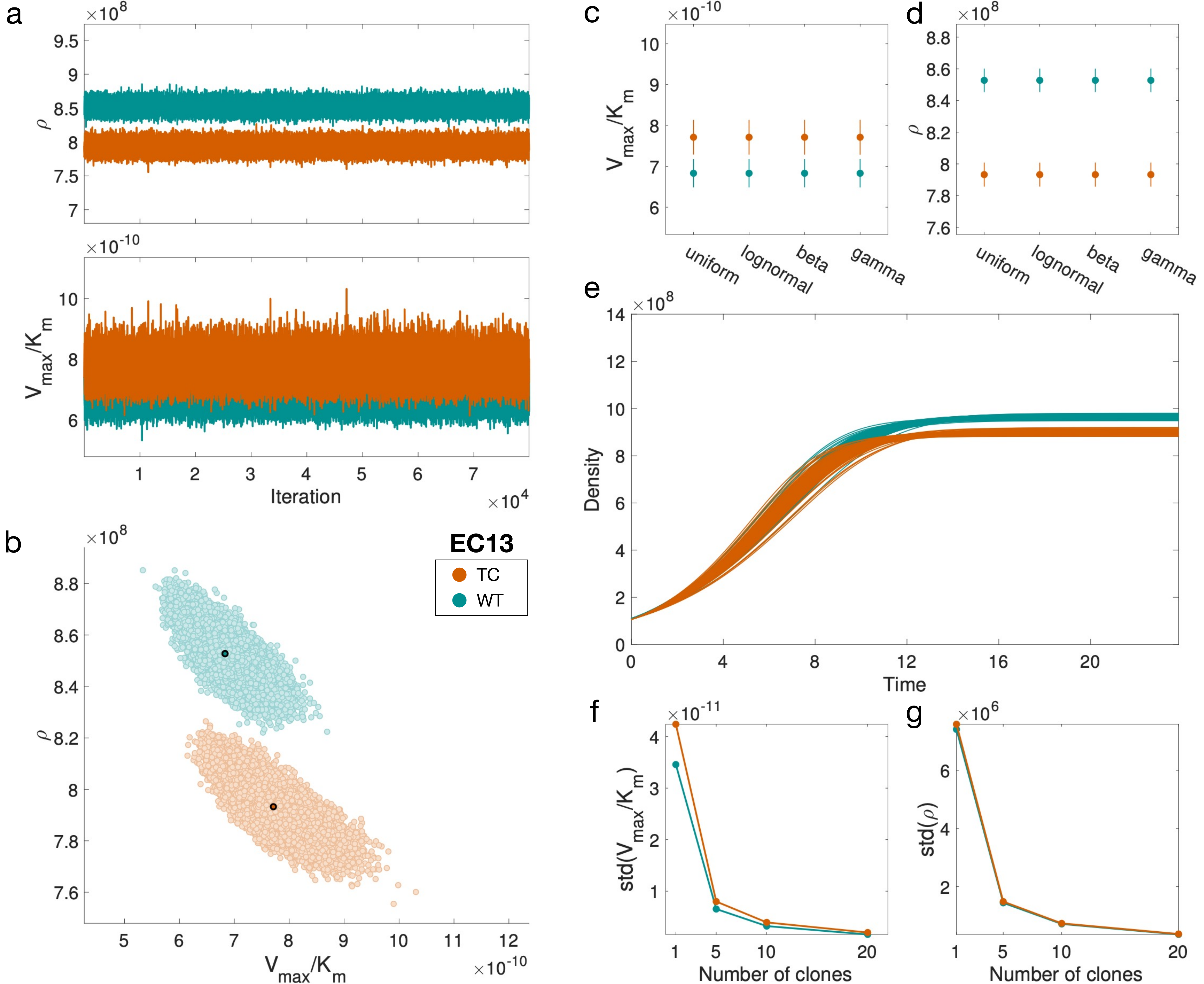

a
b
c
d
e
f
g
EC13
TC
WT
ρ
V_{max}/K_m
Iteration
$\times10^4$
$\times10^8$
$\times10^{-10}$
uniform
lognormal
beta
gamma
Density
Time
$\times10^{-11}$
$\times10^6$
std(V_{max}/K_m)
std(ρ)
Number of clones

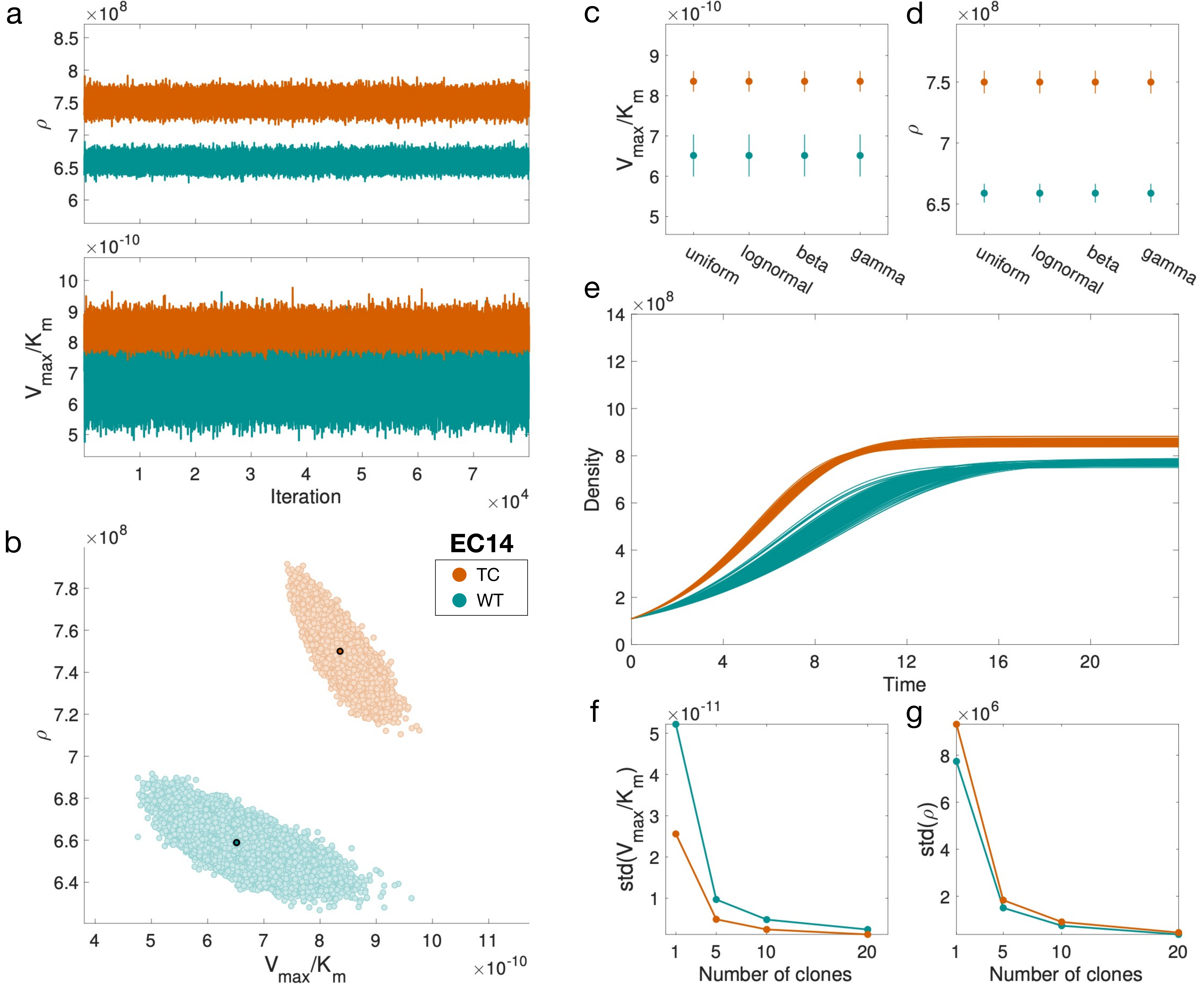

a
×10^8
ρ
×10^-10
V_max/K_m
Iteration
×10^4
b
×10^8
EC14
TC
WT
ρ
V_max/K_m
×10^-10
c
×10^-10
V_max/K_m
uniform
lognormal
beta
gamma
d
×10^8
ρ
e
×10^8
Density
Time
f
×10^-11
std(V_max/K_m)
Number of clones
g
×10^6
std(ρ)
Number of clones

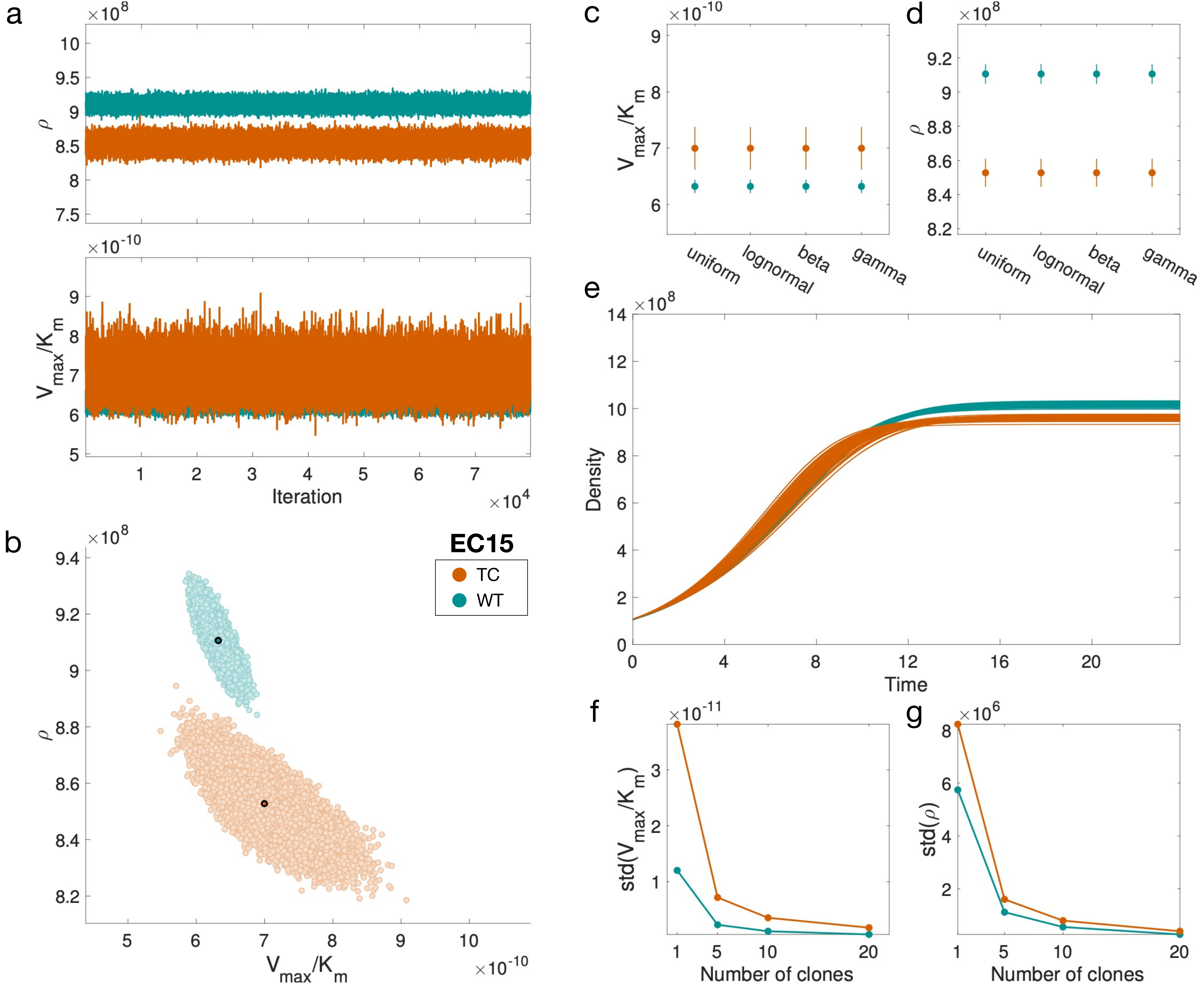

a
×10^8
10
9.5
9
8.5
8
7.5
ρ
×10^-10
9
8
7
6
5
V_max/K_m
1
2
3
4
5
6
7
Iteration
×10^4
b
×10^8
9.4
9.2
9
8.8
8.6
8.4
8.2
ρ
EC15
TC
WT
5
6
7
8
9
10
V_max/K_m
×10^-10
c
×10^-10
9
8
7
6
V_max/K_m
uniform
lognormal
beta
gamma
d
×10^8
9.2
9
8.8
8.6
8.4
8.2
ρ
uniform
lognormal
beta
gamma
e
×10^8
14
12
10
8
6
4
2
0
Density
0
4
8
12
16
20
Time
f
×10^-11
3
2
1
std(V_max/K_m)
1
5
10
20
Number of clones
g
×10^6
8
6
4
2
std(ρ)
1
5
10
20
Number of clones

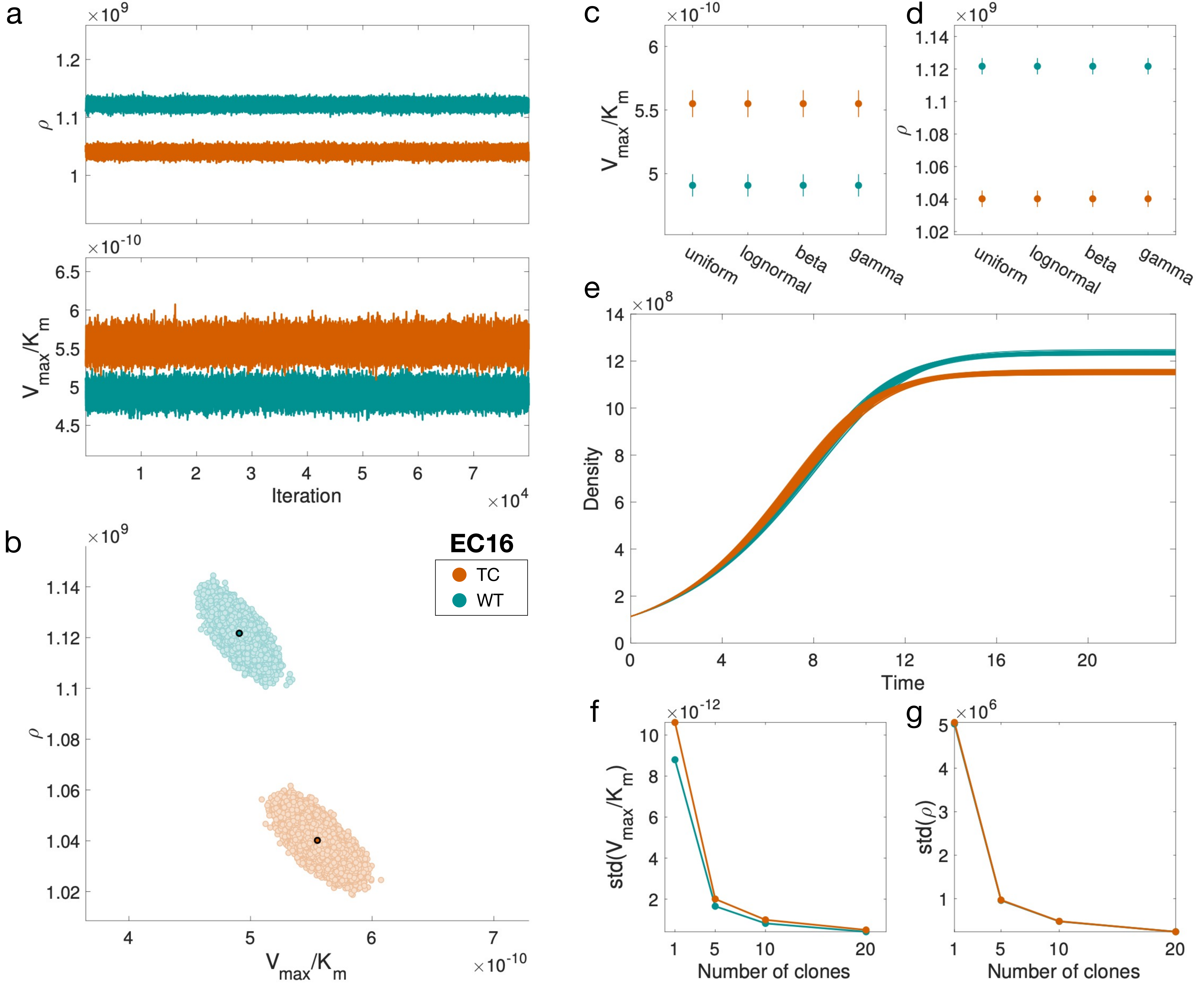

a
b
c
d
e
f
g
EC16
TC
WT
Iteration
$\times 10^4$
$\times 10^9$
$\times 10^{-10}$
ρ
V_{max}/K_m
uniform
lognormal
beta
gamma
$\times 10^8$
Density
Time
$\times 10^{-12}$
$\times 10^6$
std(V_{max}/K_m)
std(ρ)
Number of clones

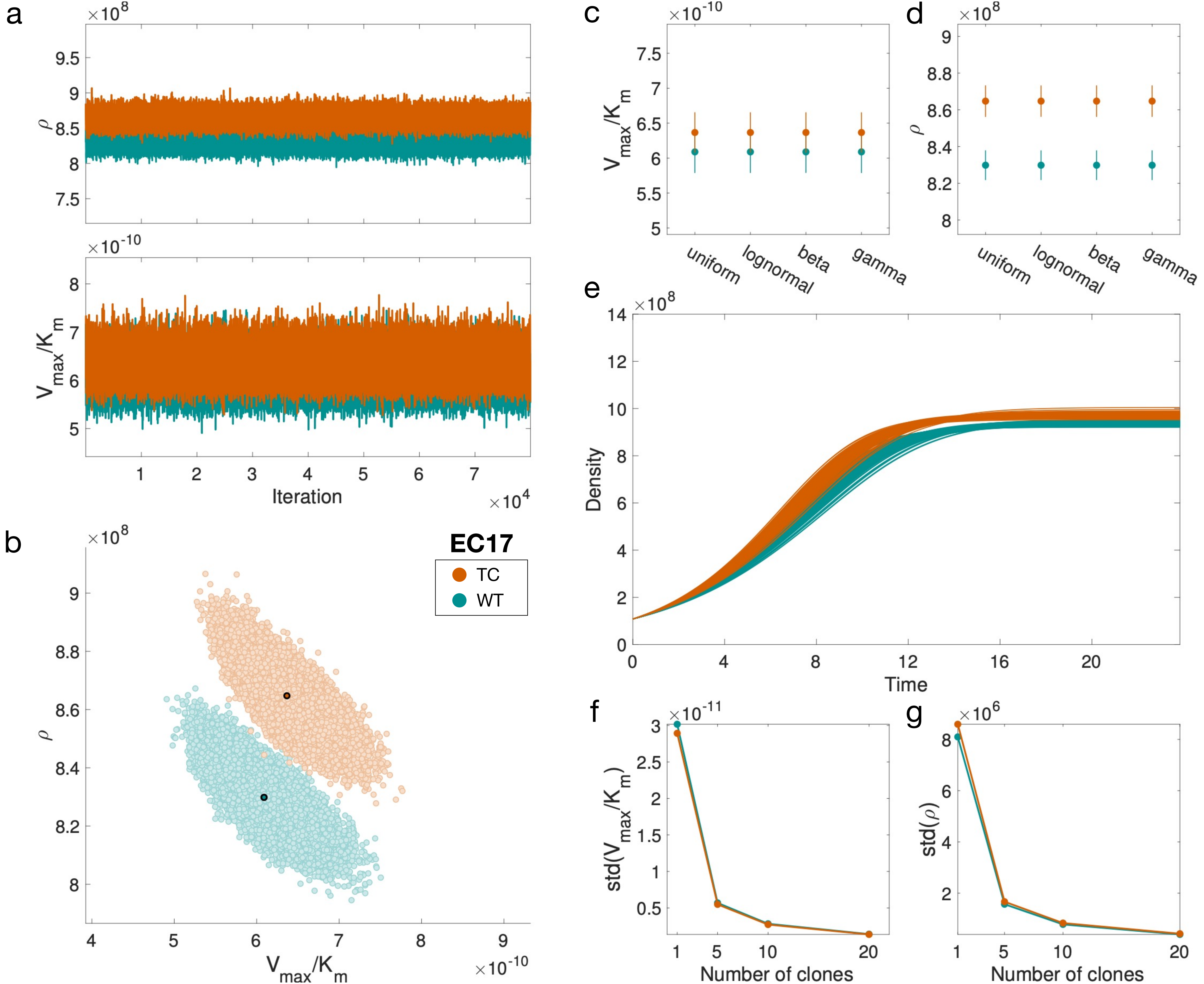

a
b
c
d
e
f
g
EC17
TC
WT
ρ
V_{max}/K_m
Iteration
uniform
lognormal
beta
gamma
Density
Time
$std(V_{max}/K_m)$
std(ρ)
Number of clones

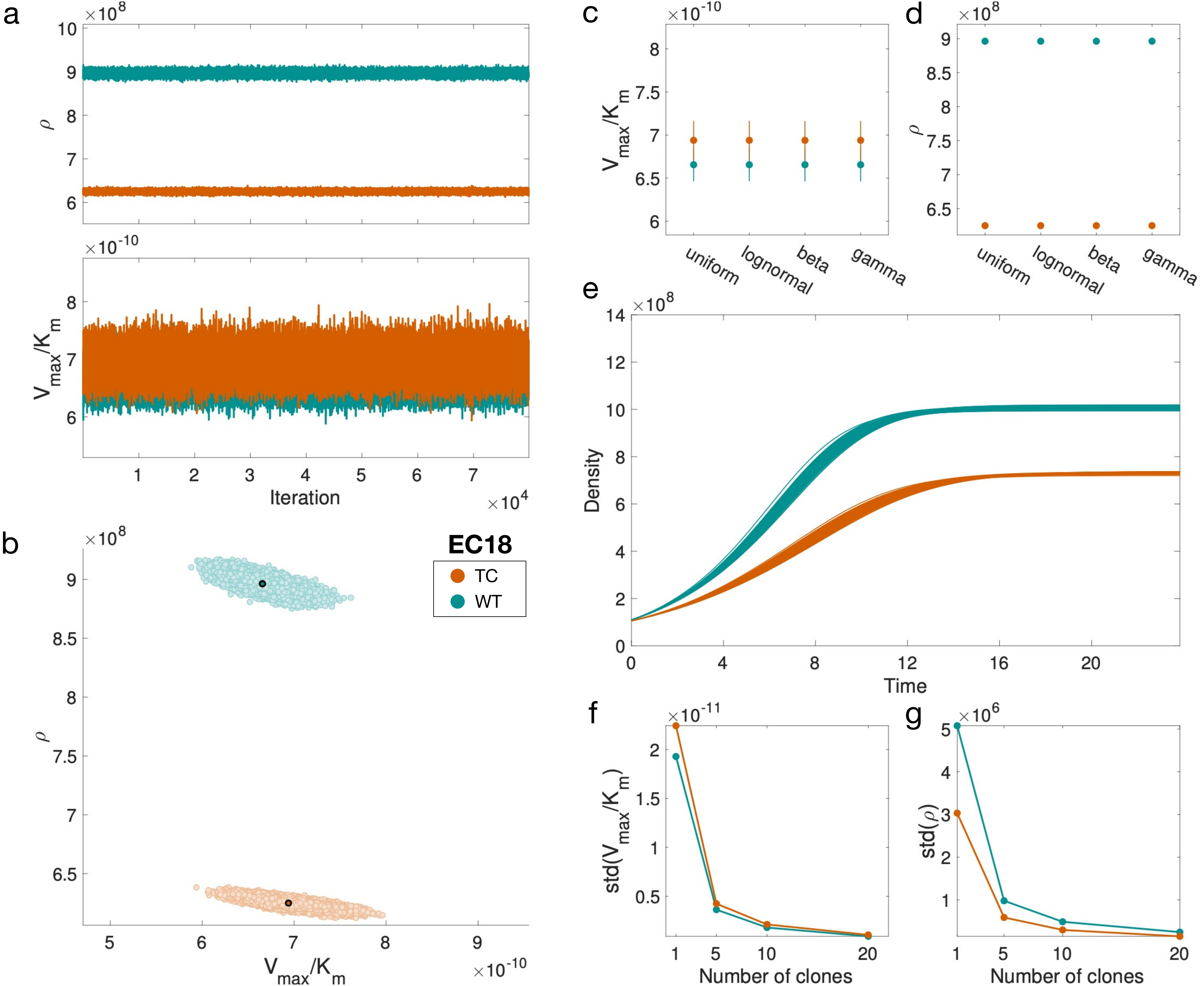

a
b
c
d
e
f
g
EC18
TC
WT
ρ
V_{max}/K_m
Iteration
uniform
lognormal
beta
gamma
Density
Time
$std(V_{max}/K_m)$
$std(\rho)$
Number of clones

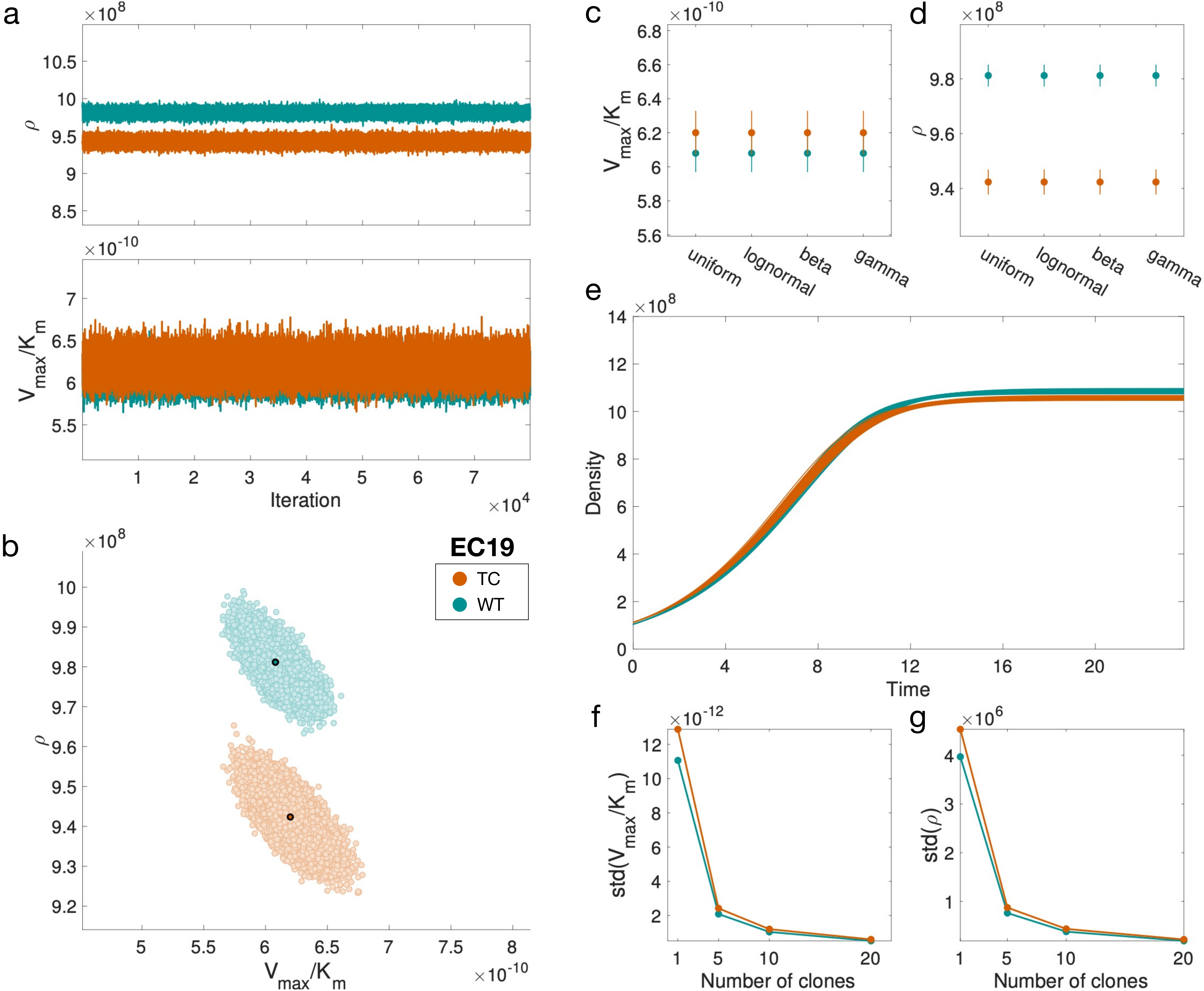

a
ρ
$\times 10^8$
V_{max}/K_m
$\times 10^{-10}$
Iteration
$\times 10^4$
b
EC19
TC
WT
ρ
$\times 10^8$
V_{max}/K_m
$\times 10^{-10}$
c
V_{max}/K_m
$\times 10^{-10}$
uniform
lognormal
beta
gamma
d
ρ
$\times 10^8$
uniform
lognormal
beta
gamma
e
Density
$\times 10^8$
Time
f
$std(V_{max}/K_m)$
$\times 10^{-12}$
Number of clones
g
std(ρ)
$\times 10^6$
Number of clones

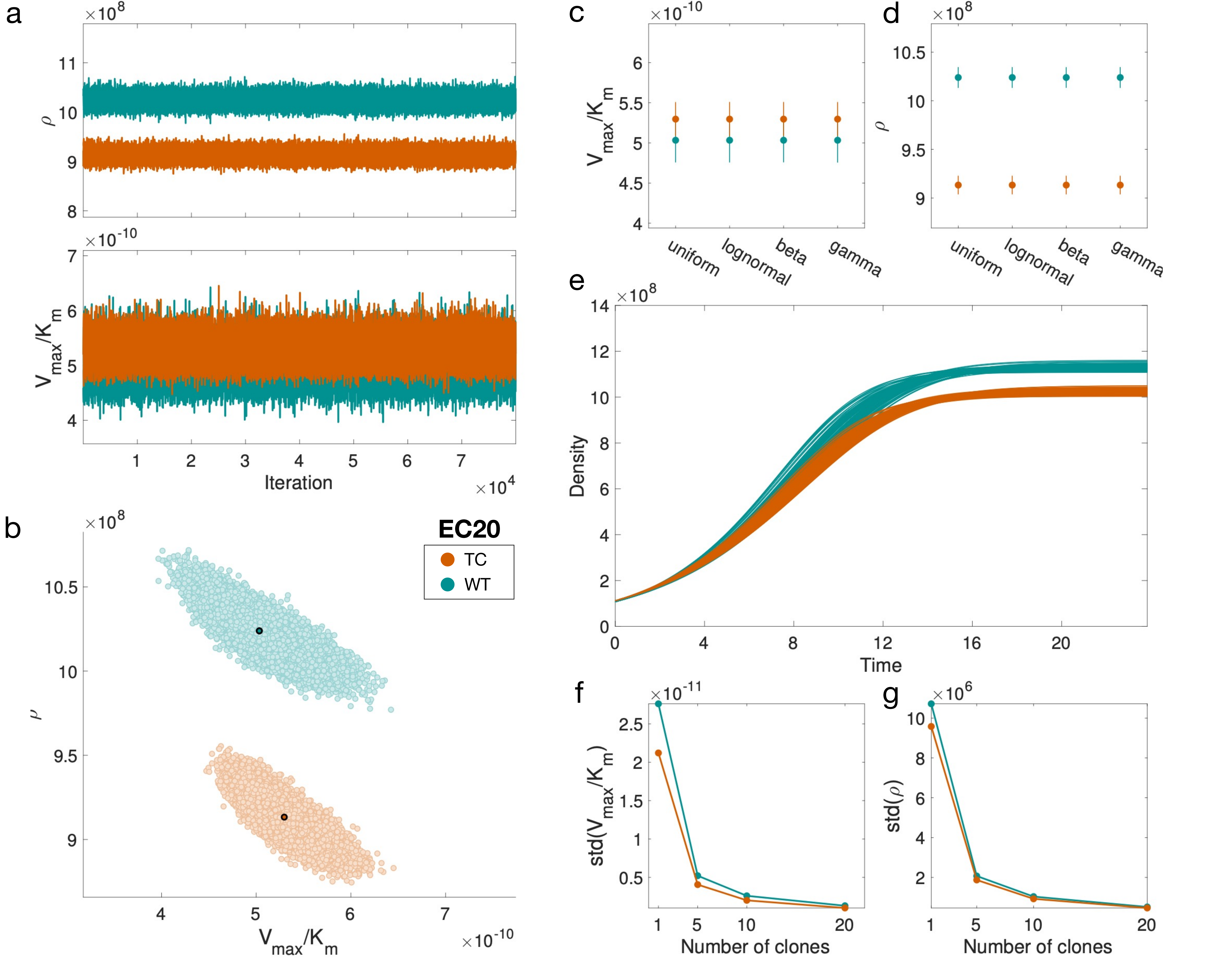

a
b
c
d
e
f
g
EC20
TC
WT
ρ
Vmax/Km
Iteration
×10^4
×10^8
×10^-10
uniform
lognormal
beta
gamma
Density
Time
std(Vmax/Km)
std(ρ)
Number of clones
×10^-11
×10^6

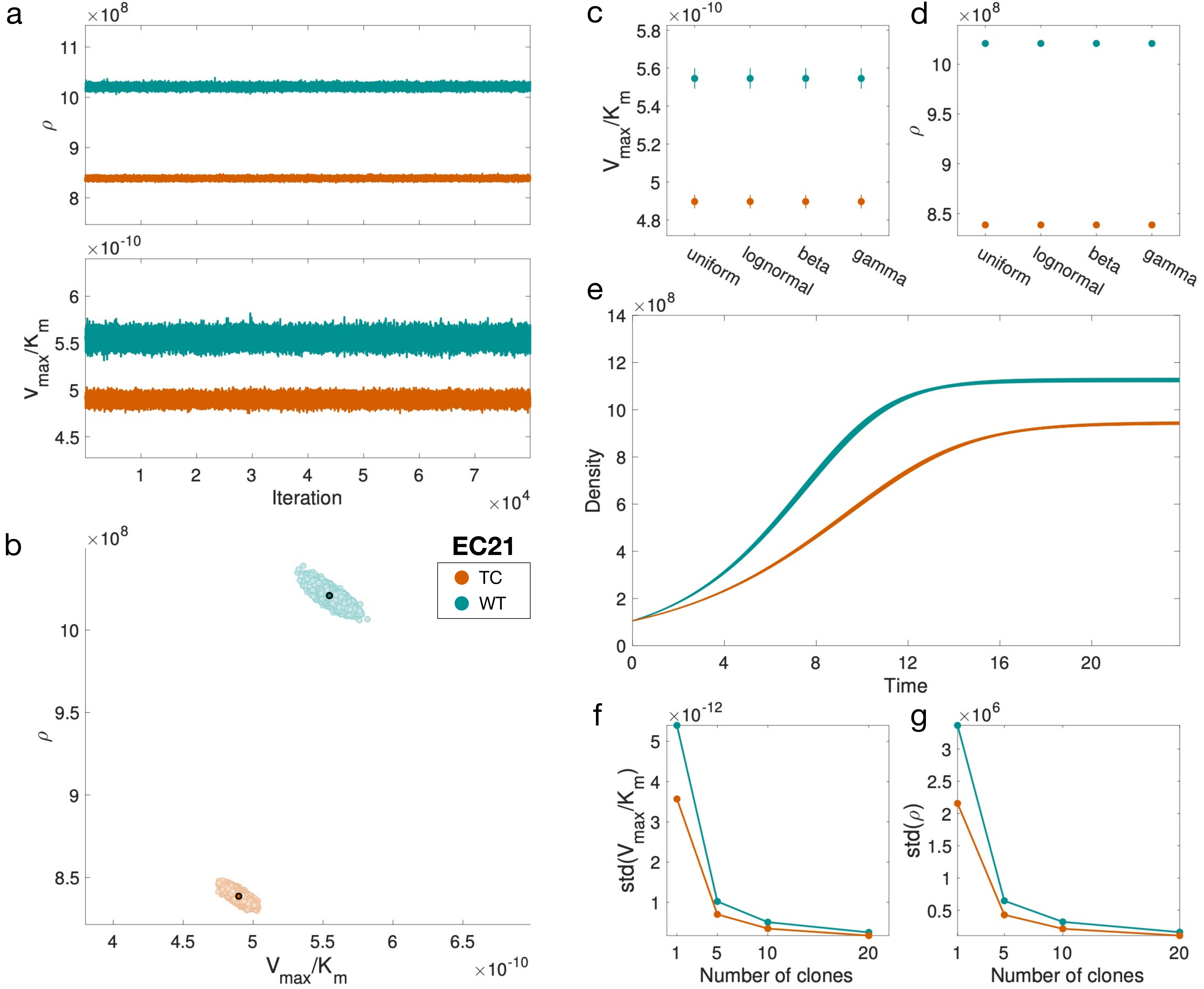

a
b
c
d
e
f
g
EC21
TC
WT
ρ
V_{max}/K_m
Iteration
Density
Time
uniform
lognormal
beta
gamma
std(V_{max}/K_m)
std(ρ)
Number of clones

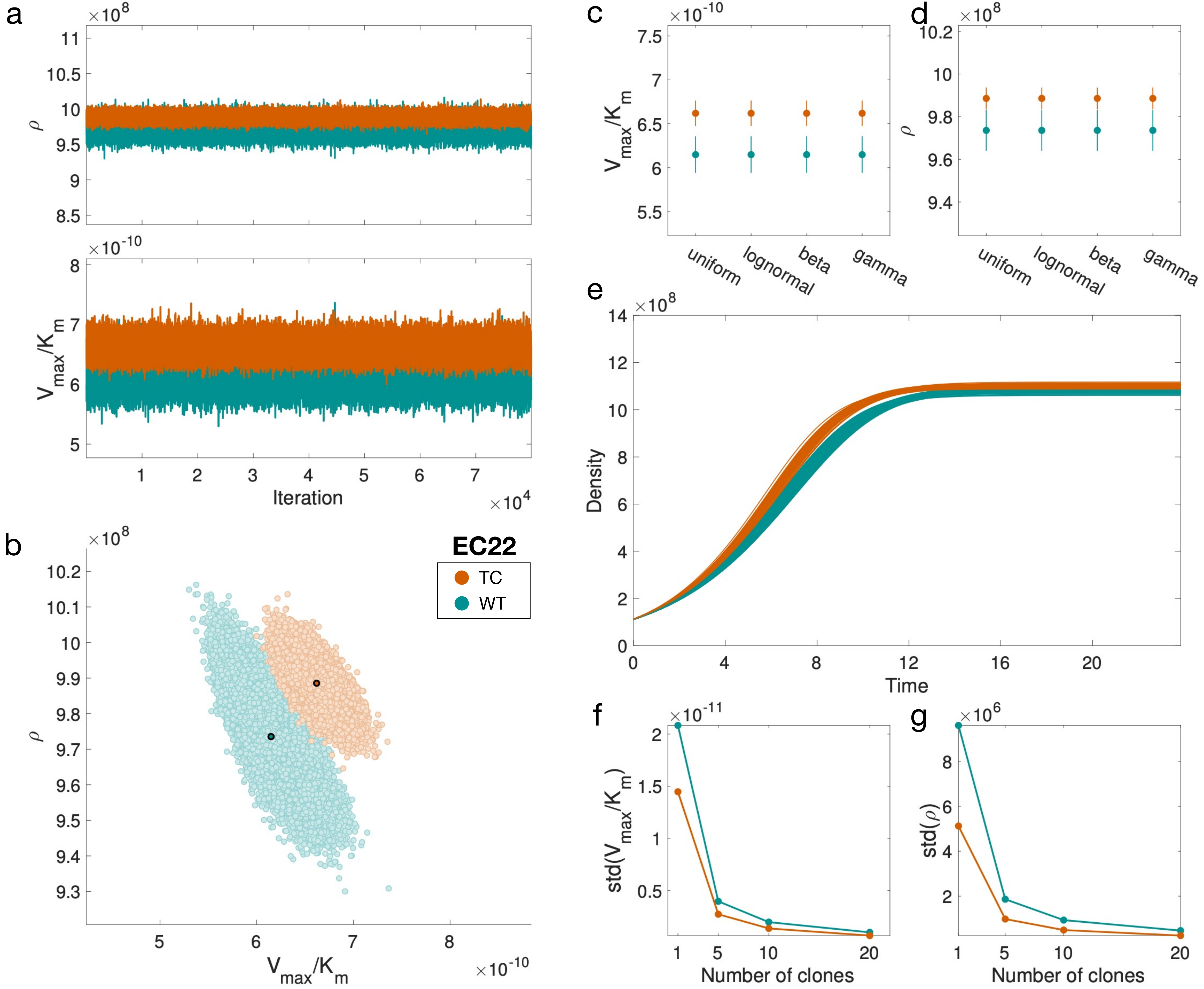
a
b
c
d
e
f
g
ρ
V_{max}/K_m
Iteration
EC22
TC
WT
uniform
lognormal
beta
gamma
Density
Time
$std(V_{max}/K_m)$
std(ρ)
Number of clones

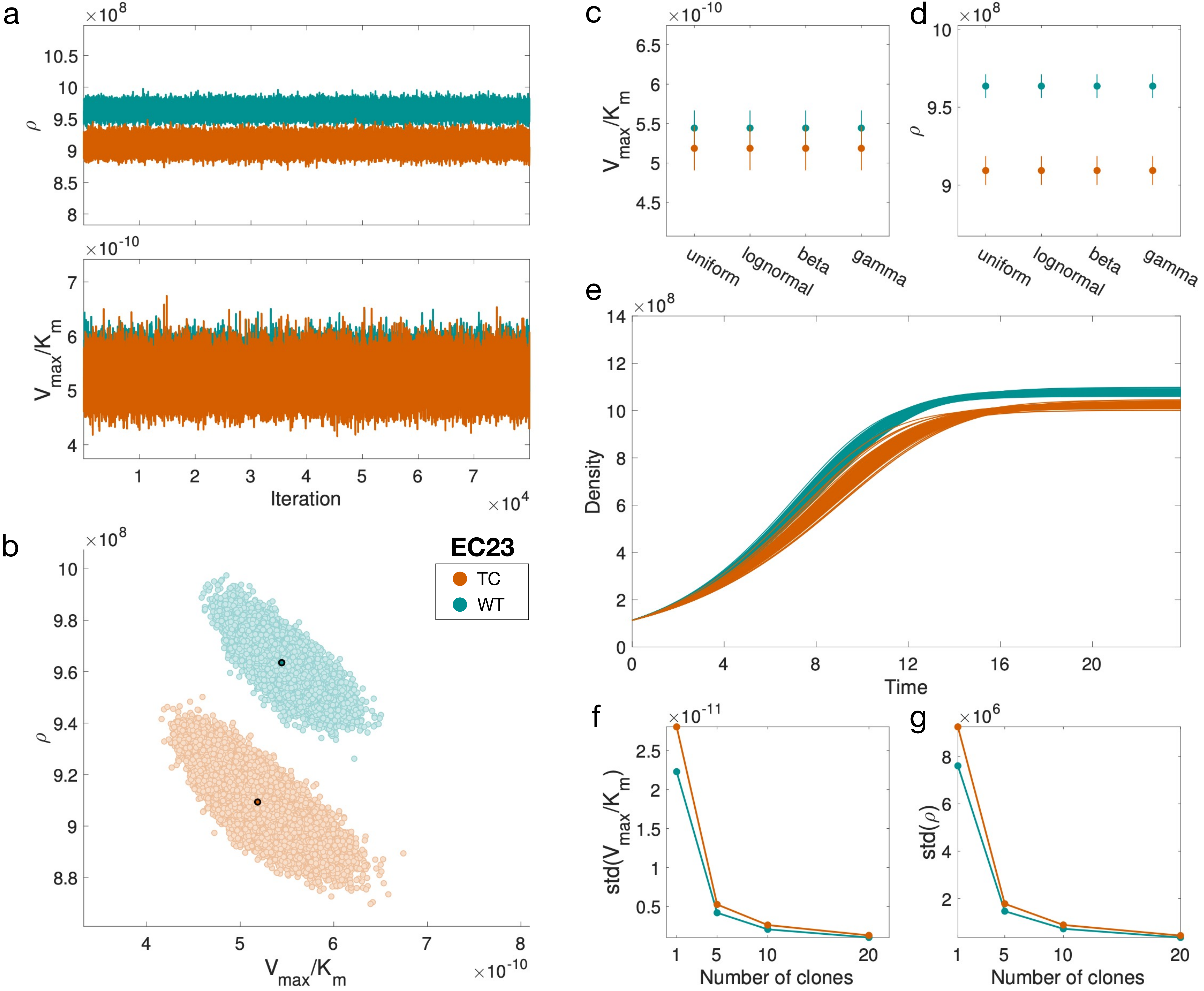
a
b
c
d
e
f
g
EC23
TC
WT
Iteration
Time
Density
Number of clones
uniform
lognormal
beta
gamma

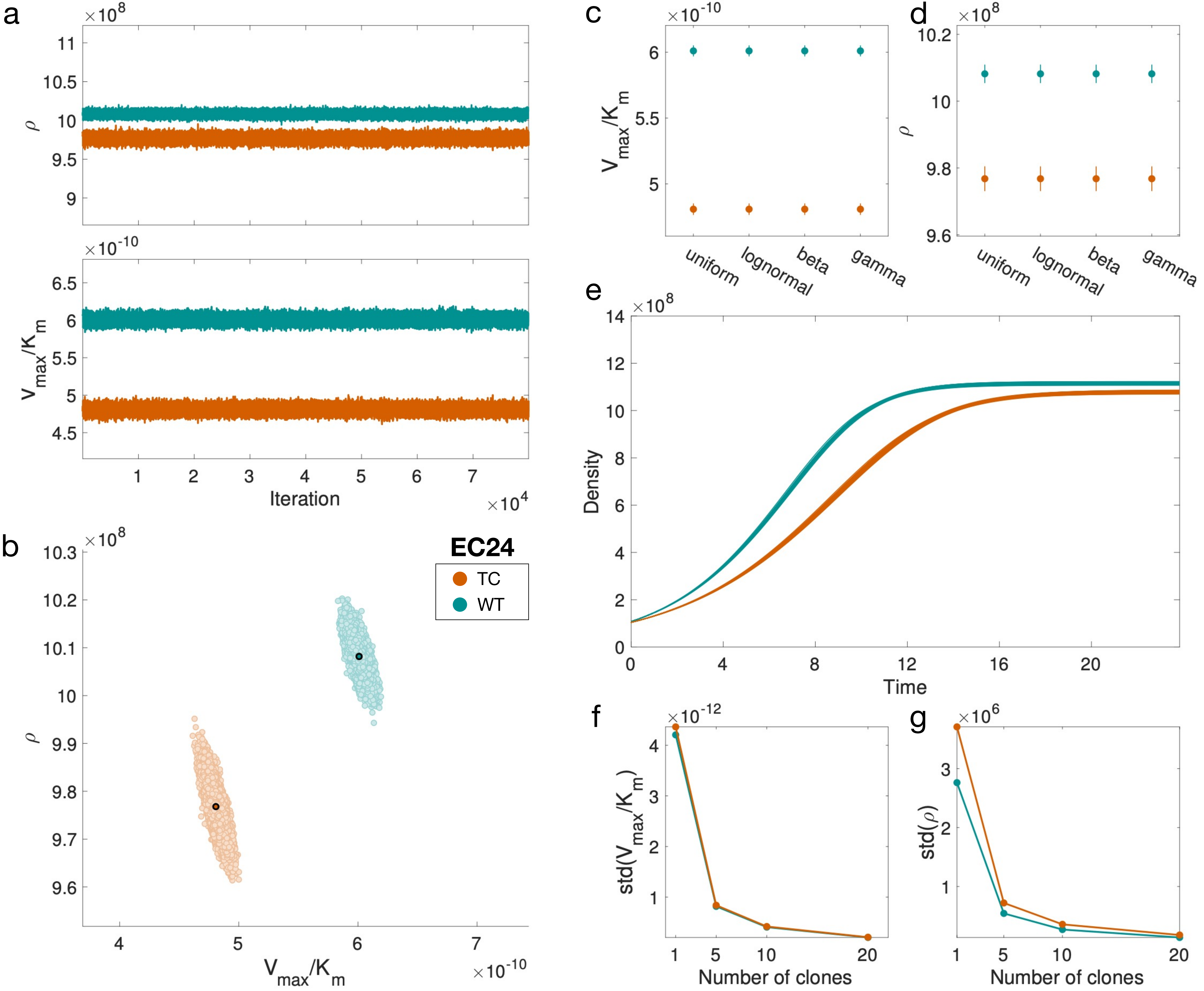
a
ρ
×10^8
V_{max}/K_m
×10^-10
Iteration
×10^4
b
EC24
TC
WT
ρ
V_{max}/K_m
c
V_{max}/K_m
uniform
lognormal
beta
gamma
d
ρ
e
Density
Time
f
std(V_{max}/K_m)
×10^-12
Number of clones
g
std(ρ)
×10^6
Number of clones

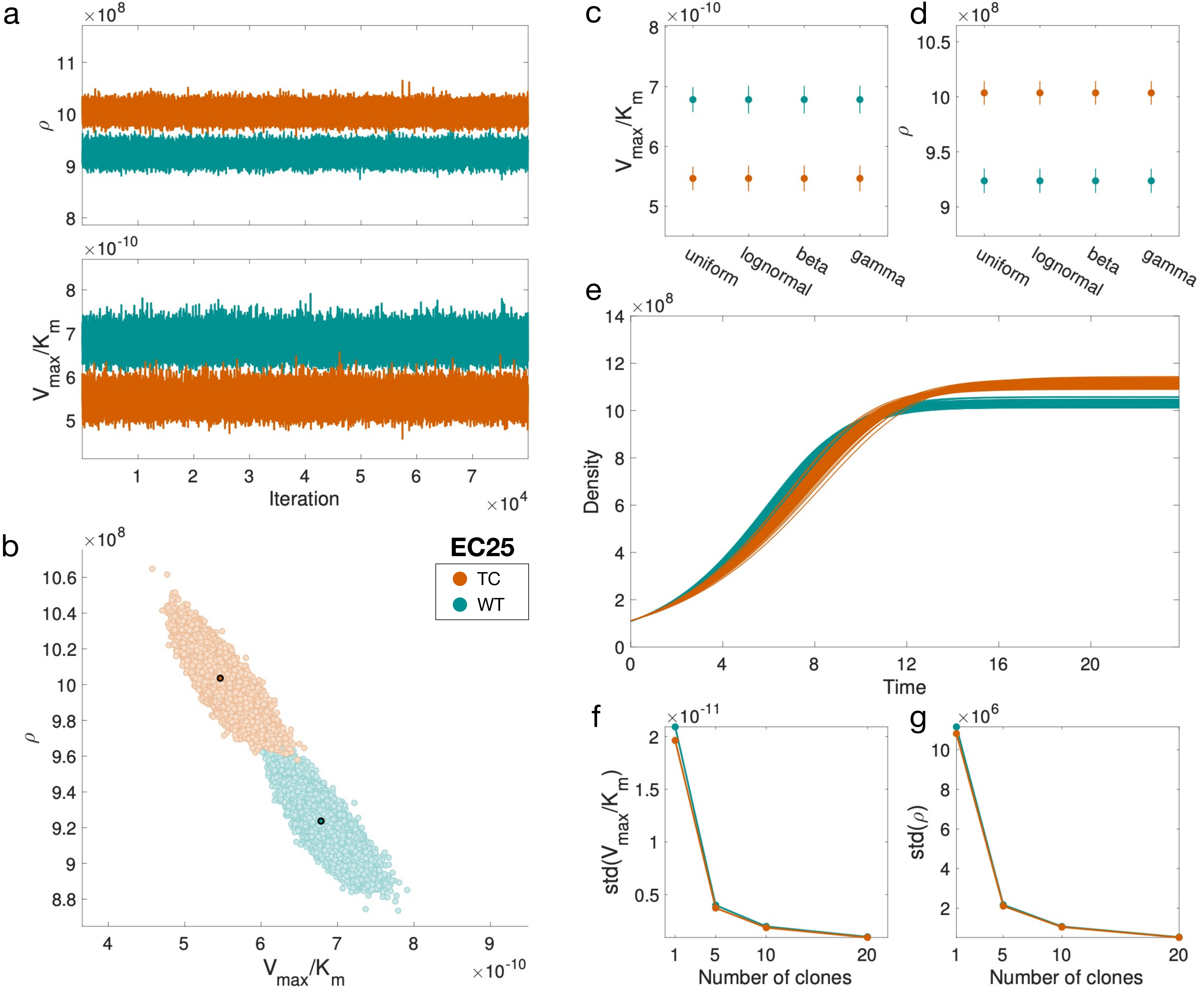

a
b
c
d
e
f
g
Iteration
ρ
V_{max}/K_m
EC25
TC
WT
uniform
lognormal
beta
gamma
Density
Time
$std(V_{max}/K_m)$
std(ρ)
Number of clones

Klebsiella spp.

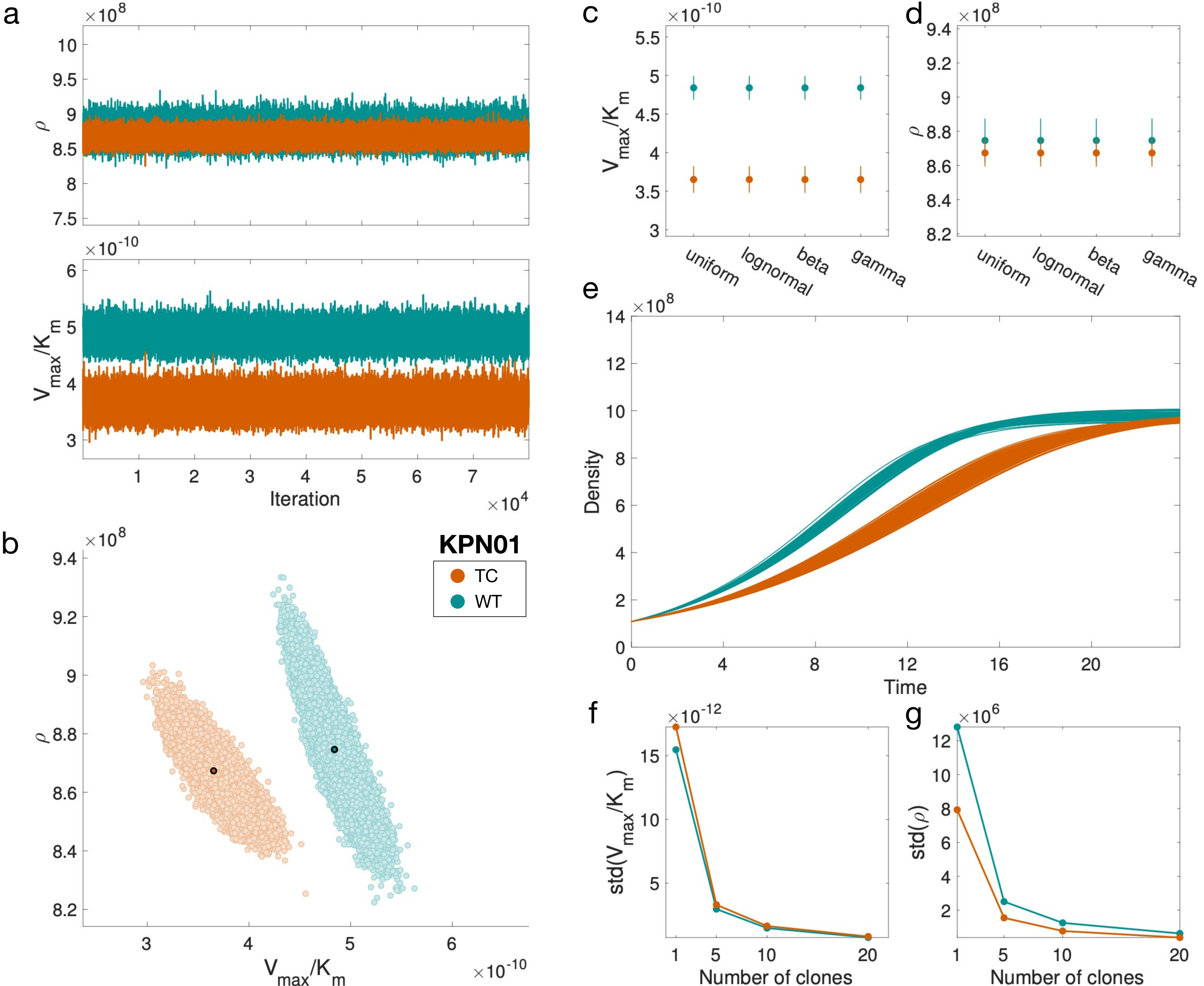

a
ρ
×10^8
Vmax/Km
×10^-10
Iteration
×10^4
b
KPN01
TC
WT
ρ
×10^8
Vmax/Km
×10^-10
c
Vmax/Km
×10^-10
uniform
lognormal
beta
gamma
d
ρ
×10^8
uniform
lognormal
beta
gamma
e
Density
×10^8
Time
f
std(Vmax/Km)
×10^-12
Number of clones
g
std(ρ)
×10^6
Number of clones

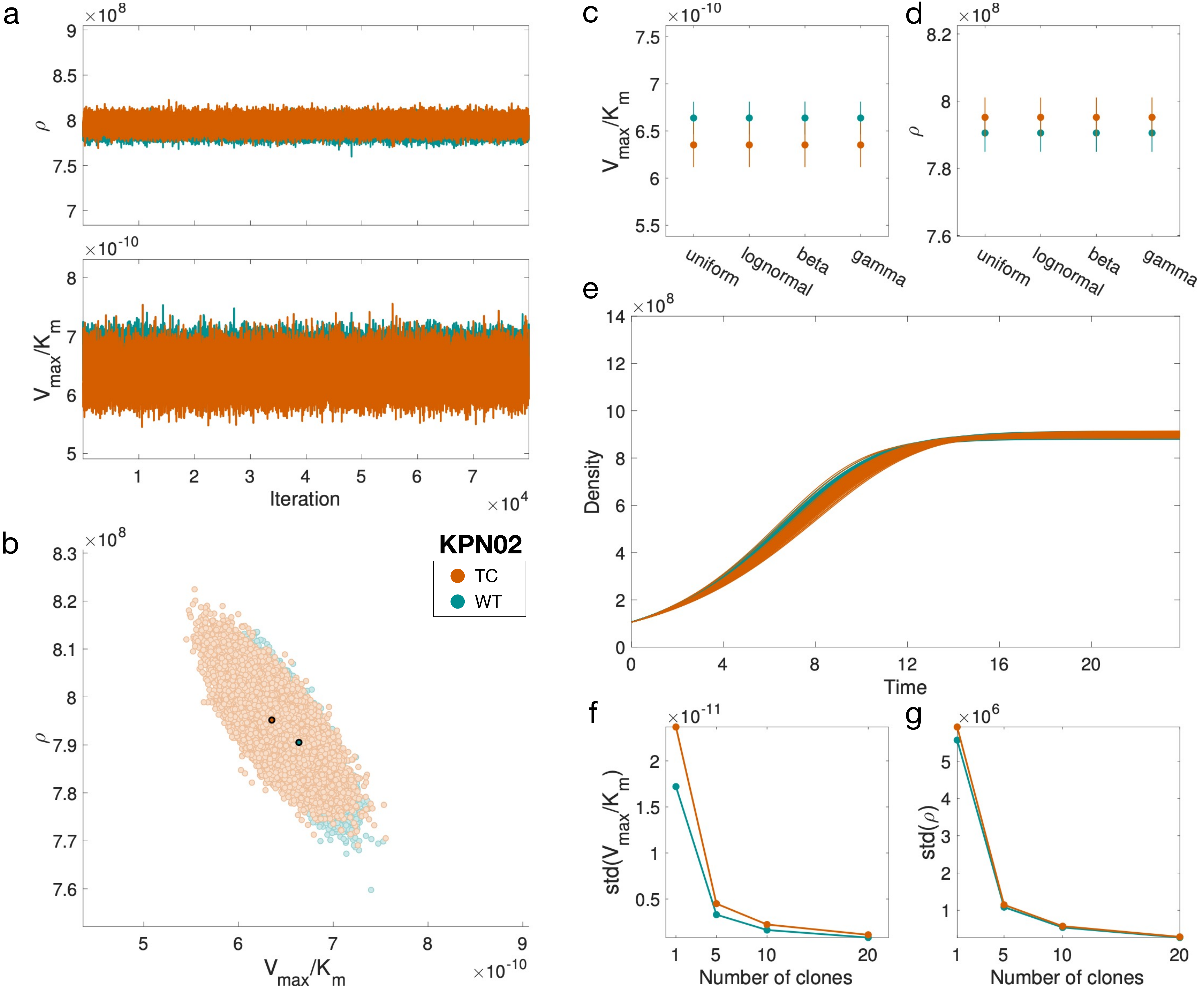

a
c
d
e
b
f
g
KPN02
TC
WT
Iteration
Time
Density
Number of clones
uniform
lognormal
beta
gamma

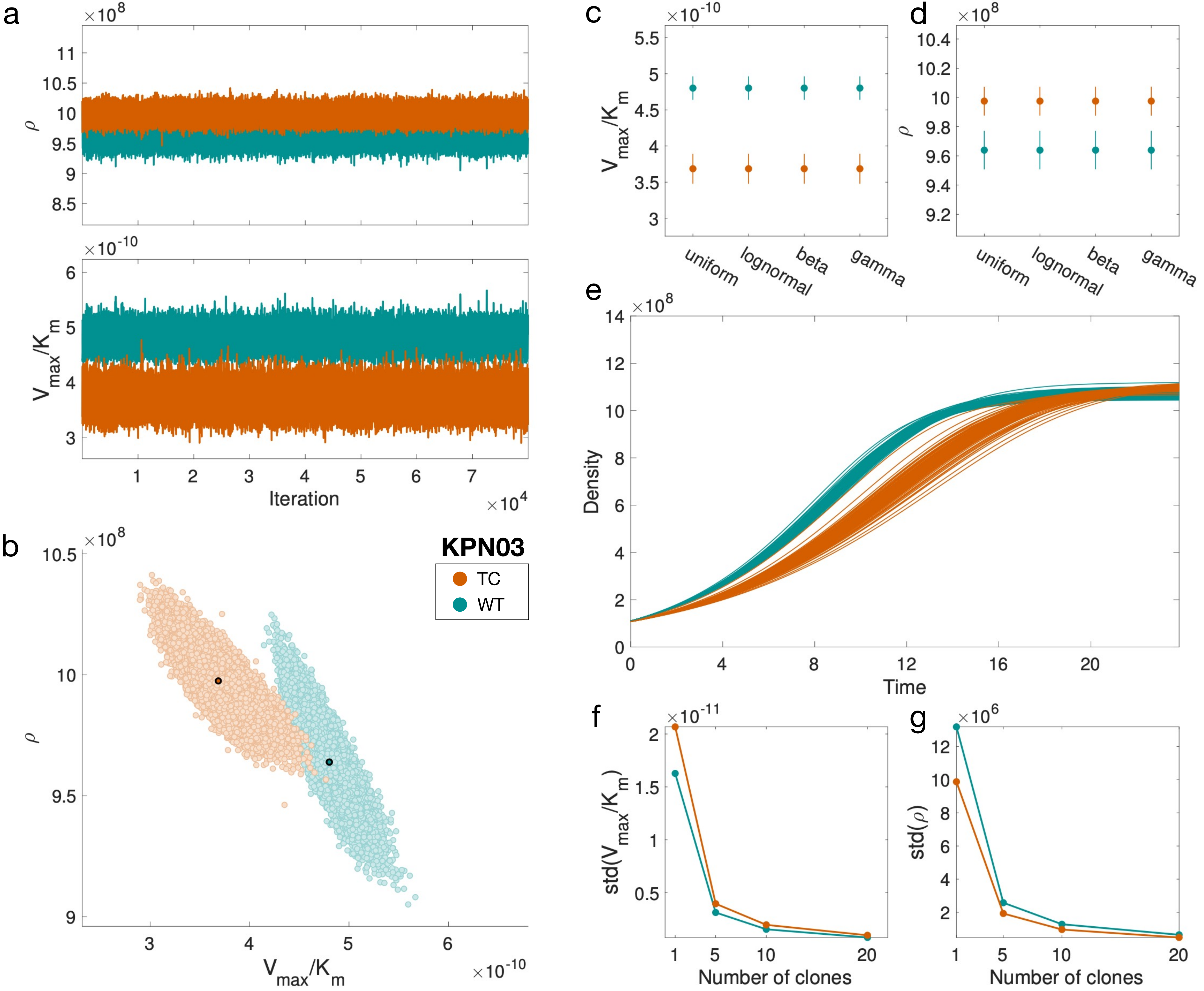
a
b
c
d
e
f
g
KPN03
TC
WT
Iteration
Density
Time
Number of clones
uniform
lognormal
beta
gamma

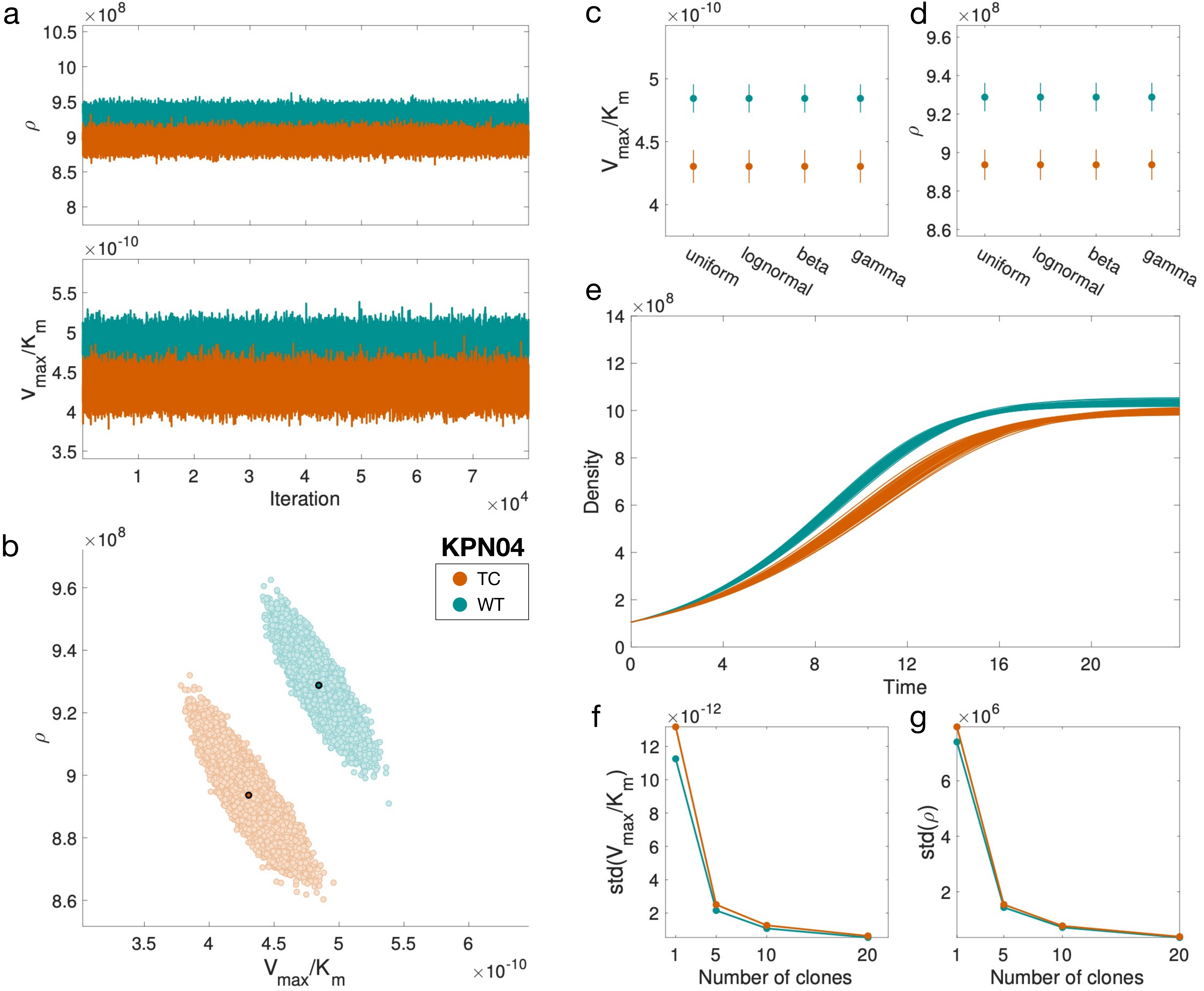

a
b
KPN04
TC
WT
c
d
e
f
g
Iteration
Density
Time
Number of clones
uniform
lognormal
beta
gamma

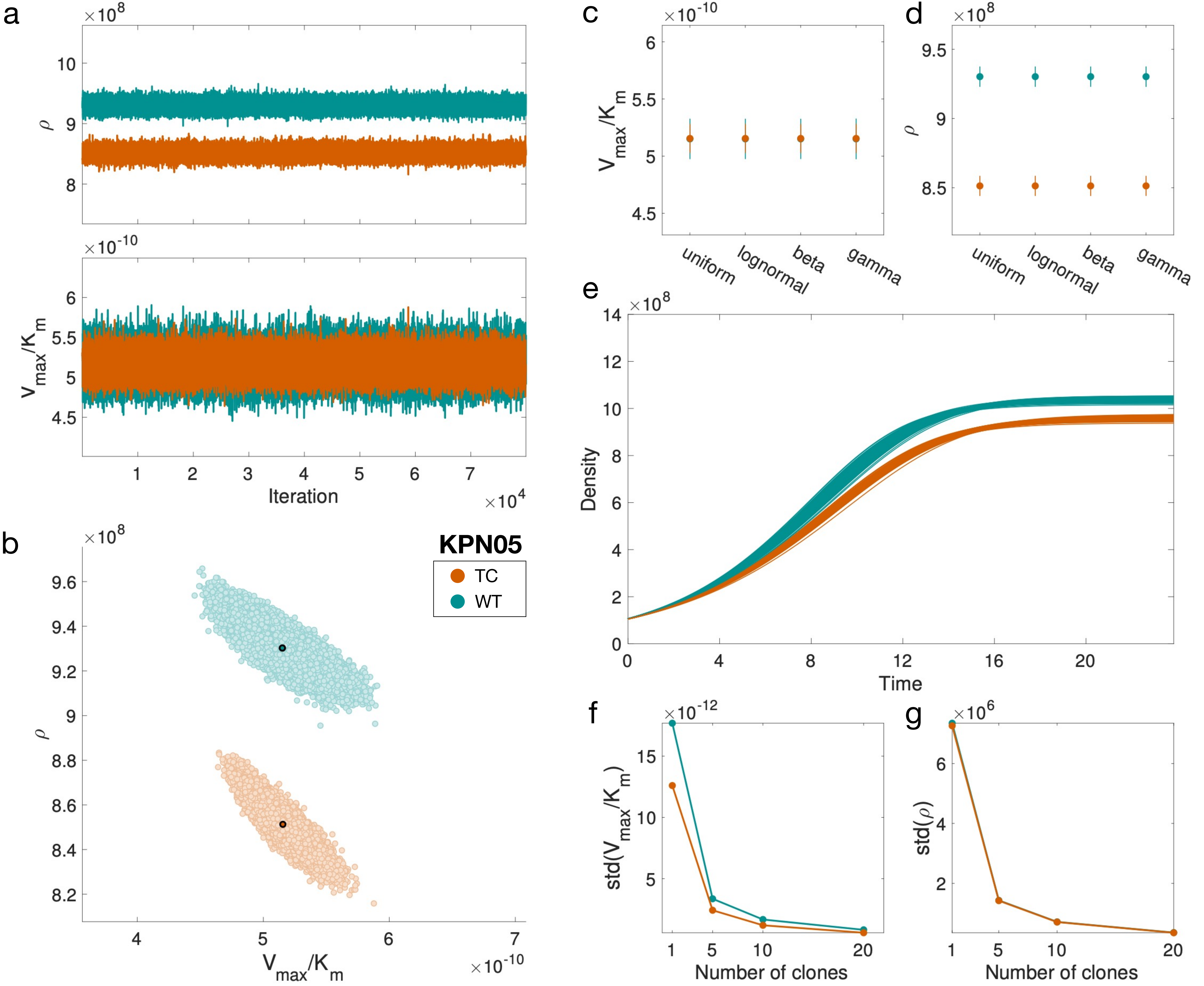

a
b
c
d
e
f
g
KPN05
TC
WT
Iteration
Density
Time
Number of clones
uniform
lognormal
beta
gamma

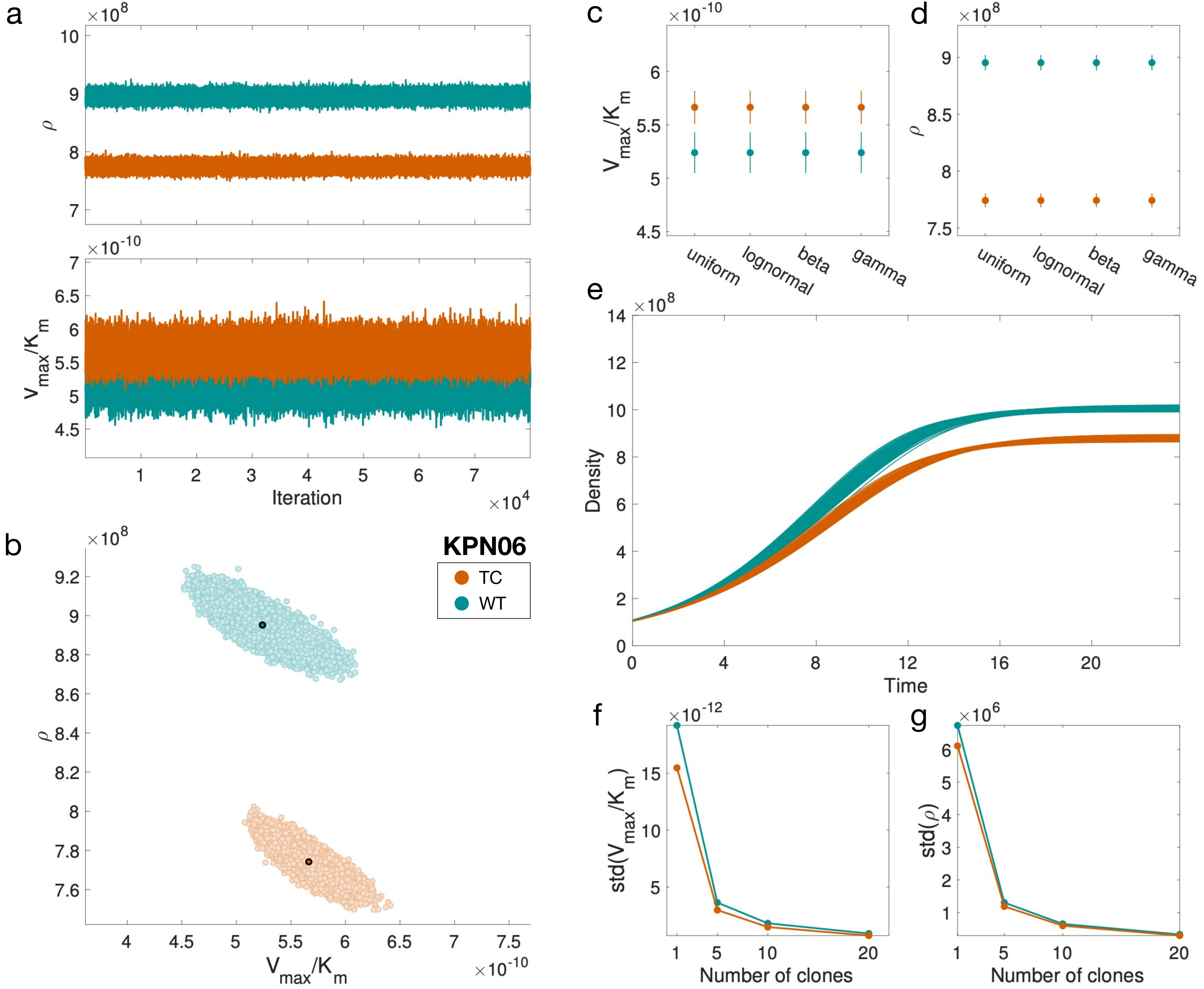
a
b
c
d
e
f
g
KPN06
TC
WT
ρ
V_{max}/K_m
Iteration
uniform
lognormal
beta
gamma
Density
Time
std(V_{max}/K_m)
std(ρ)
Number of clones

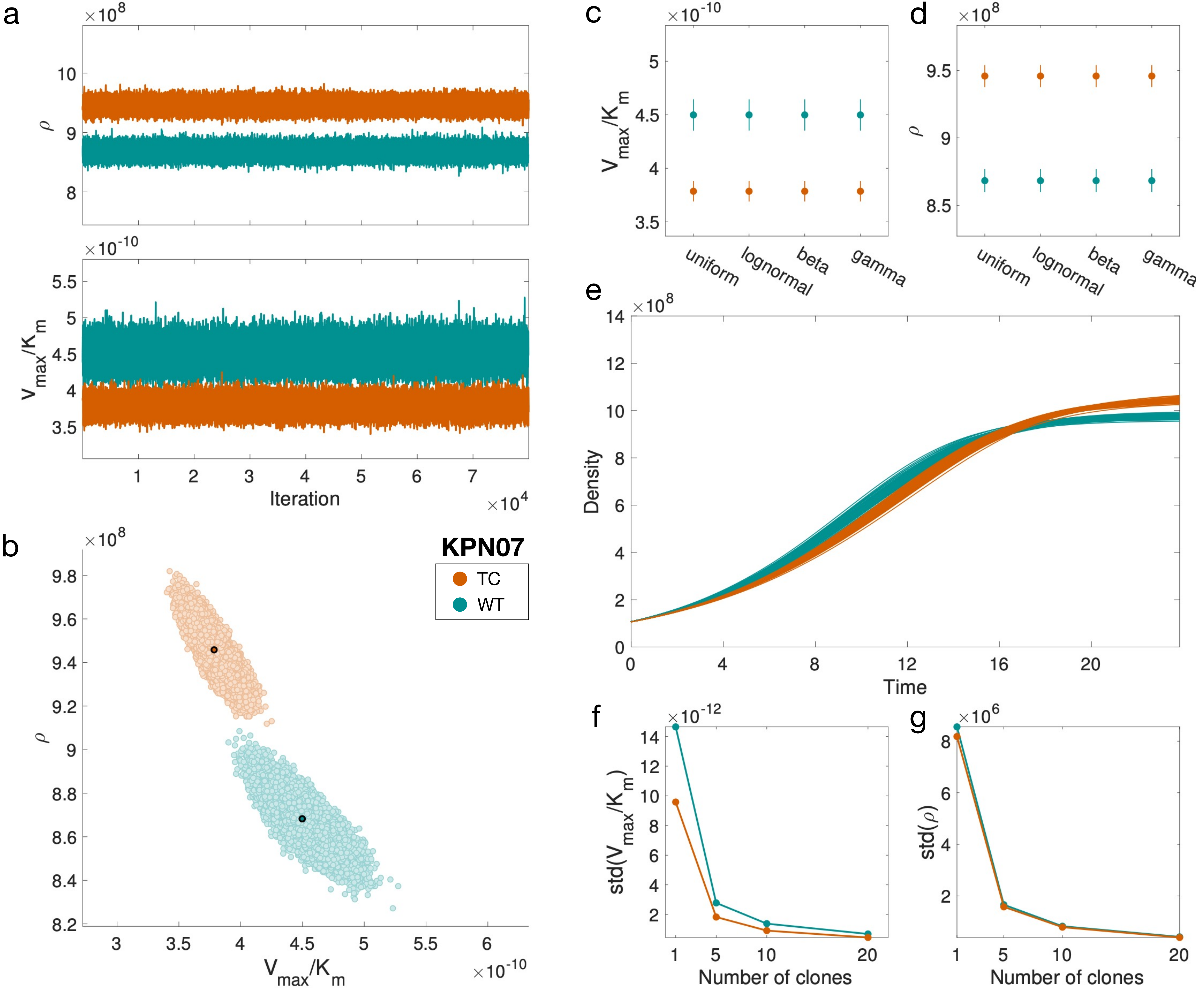

a
c
d
b
e
f
g
KPN07
TC
WT
Iteration
Time
Density
Number of clones
uniform
lognormal
beta
gamma
ρ
V_{max}/K_m
std(V_{max}/K_m)
std(ρ)

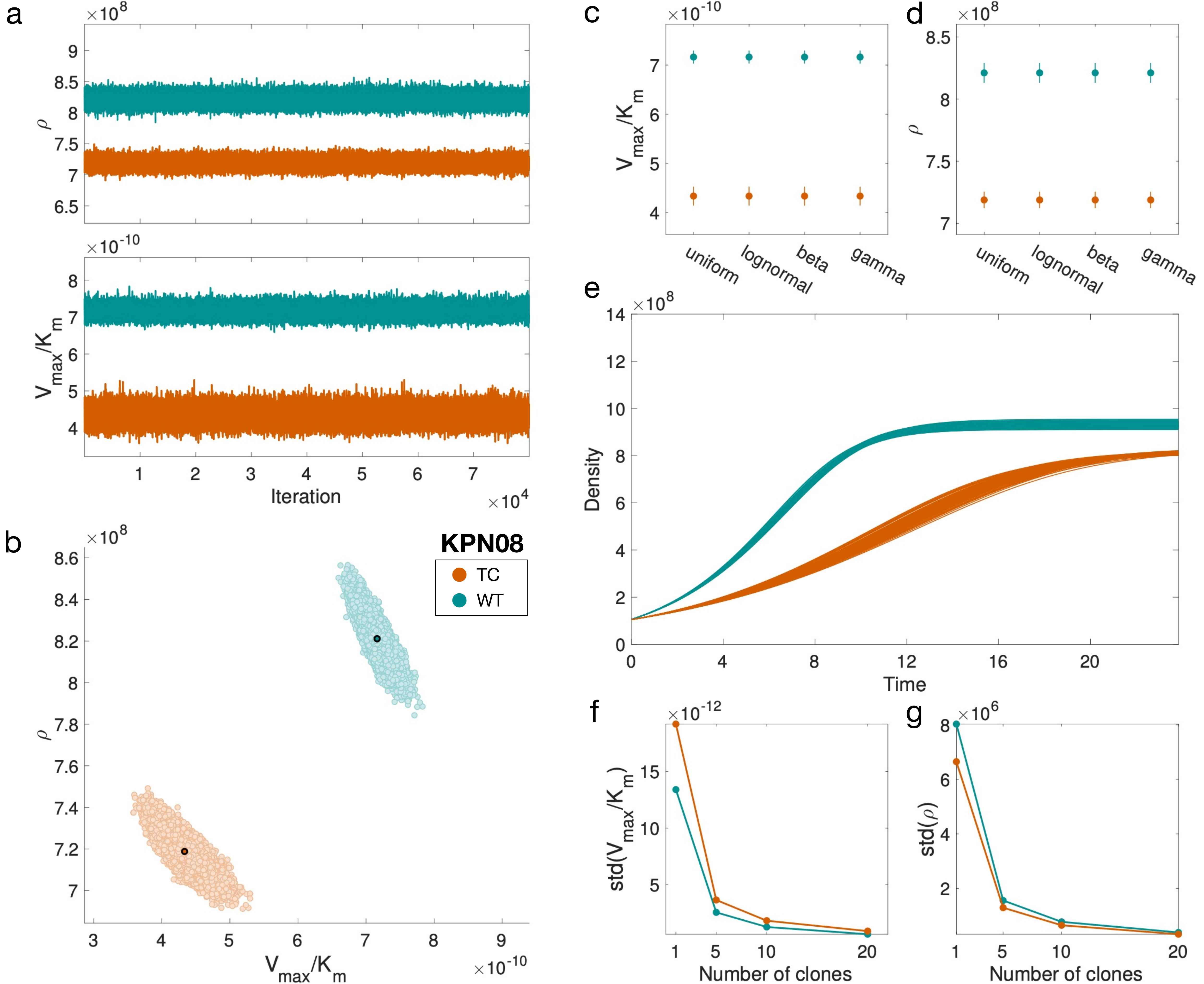

a
$\times 10^{8}$
ρ
9
8.5
8
7.5
7
6.5
$\times 10^{-10}$
V_{max}/K_m
8
7
6
5
4
1
2
3
4
5
6
7
Iteration
$\times 10^{4}$
b
$\times 10^{8}$
KPN08
TC
WT
ρ
8.6
8.4
8.2
8
7.8
7.6
7.4
7.2
7
3
4
5
6
7
8
9
V_{max}/K_m
$\times 10^{-10}$
c
$\times 10^{-10}$
V_{max}/K_m
7
6
5
4
uniform
lognormal
beta
gamma
d
$\times 10^{8}$
ρ
8.5
8
7.5
7
uniform
lognormal
beta
gamma
e
$\times 10^{8}$
Density
14
12
10
8
6
4
2
0
0
4
8
12
16
20
Time
f
$\times 10^{-12}$
std(V_{max}/K_m)
15
10
5
1
5
10
20
Number of clones
g
$\times 10^{6}$
std(ρ)
8
6
4
2
1
5
10
20
Number of clones

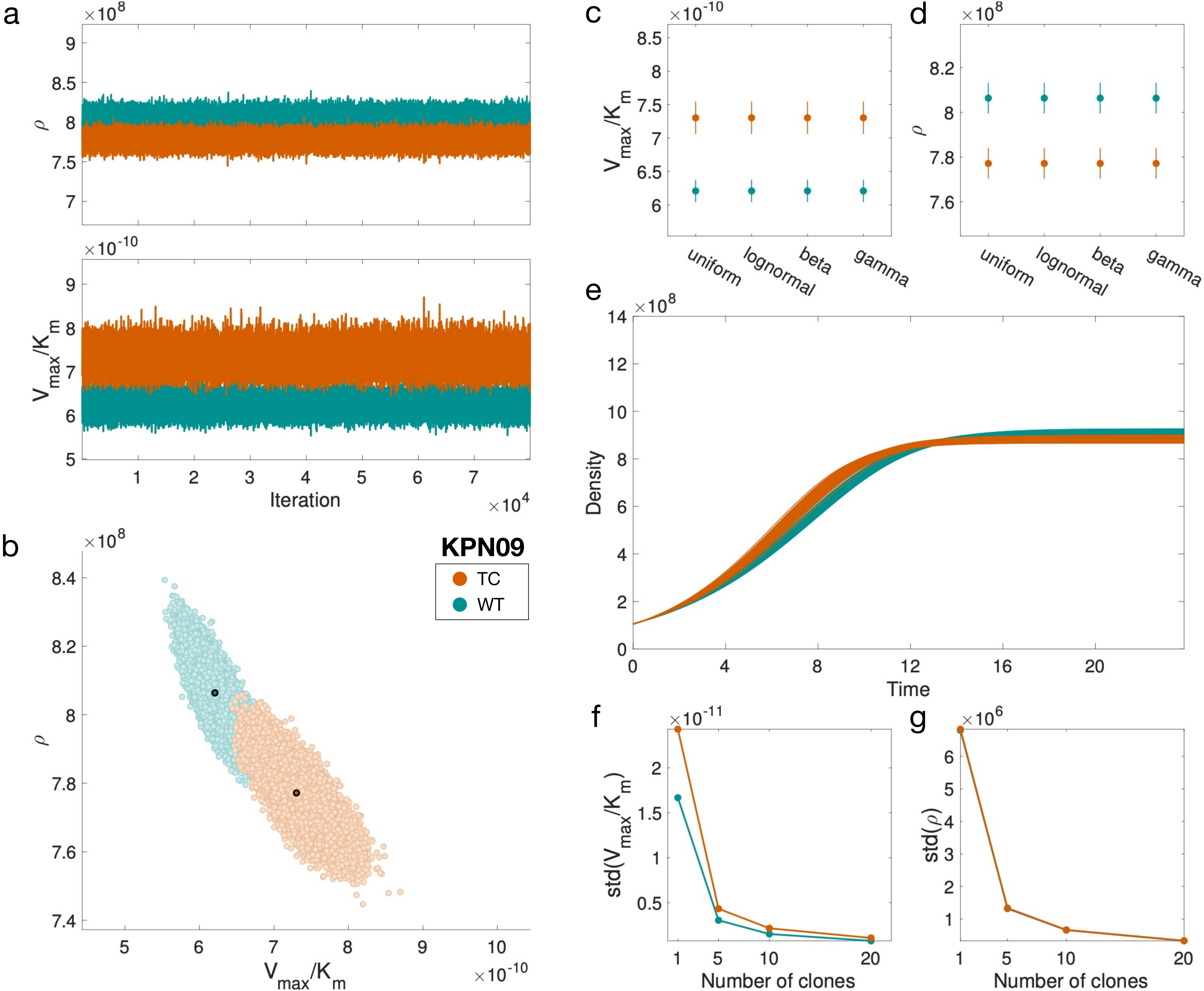

a
b
c
d
e
f
g
KPN09
TC
WT
Iteration
Time
Density
Number of clones
uniform
lognormal
beta
gamma

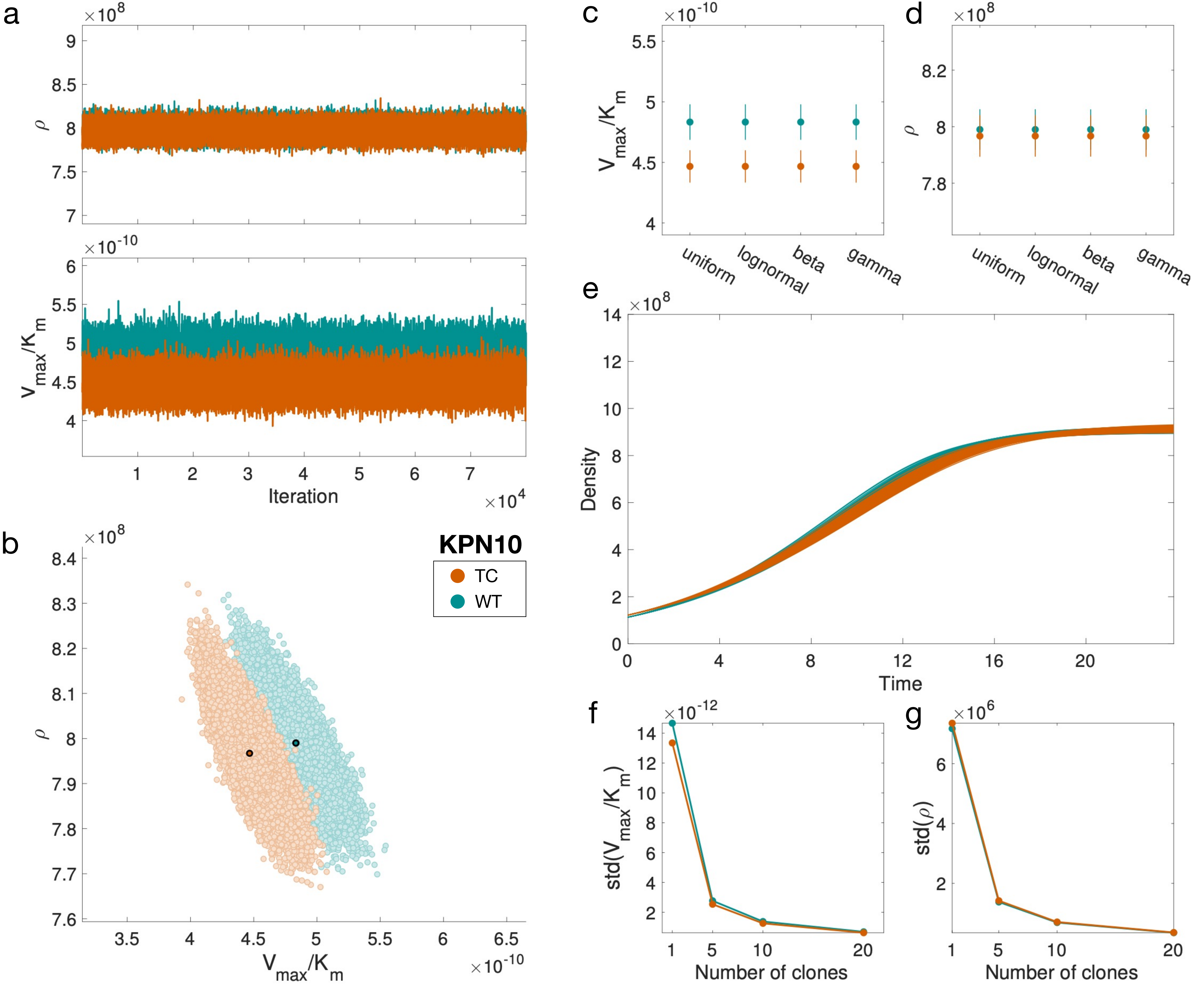

a
ρ
$\times 10^8$
V_{max}/K_m
$\times 10^{-10}$
Iteration
$\times 10^4$
b
KPN10
TC
WT
ρ
V_{max}/K_m
c
d
uniform
lognormal
beta
gamma
e
Density
Time
f
$std(V_{max}/K_m)$
$\times 10^{-12}$
Number of clones
g
$std(\rho)$
$\times 10^6$
Number of clones

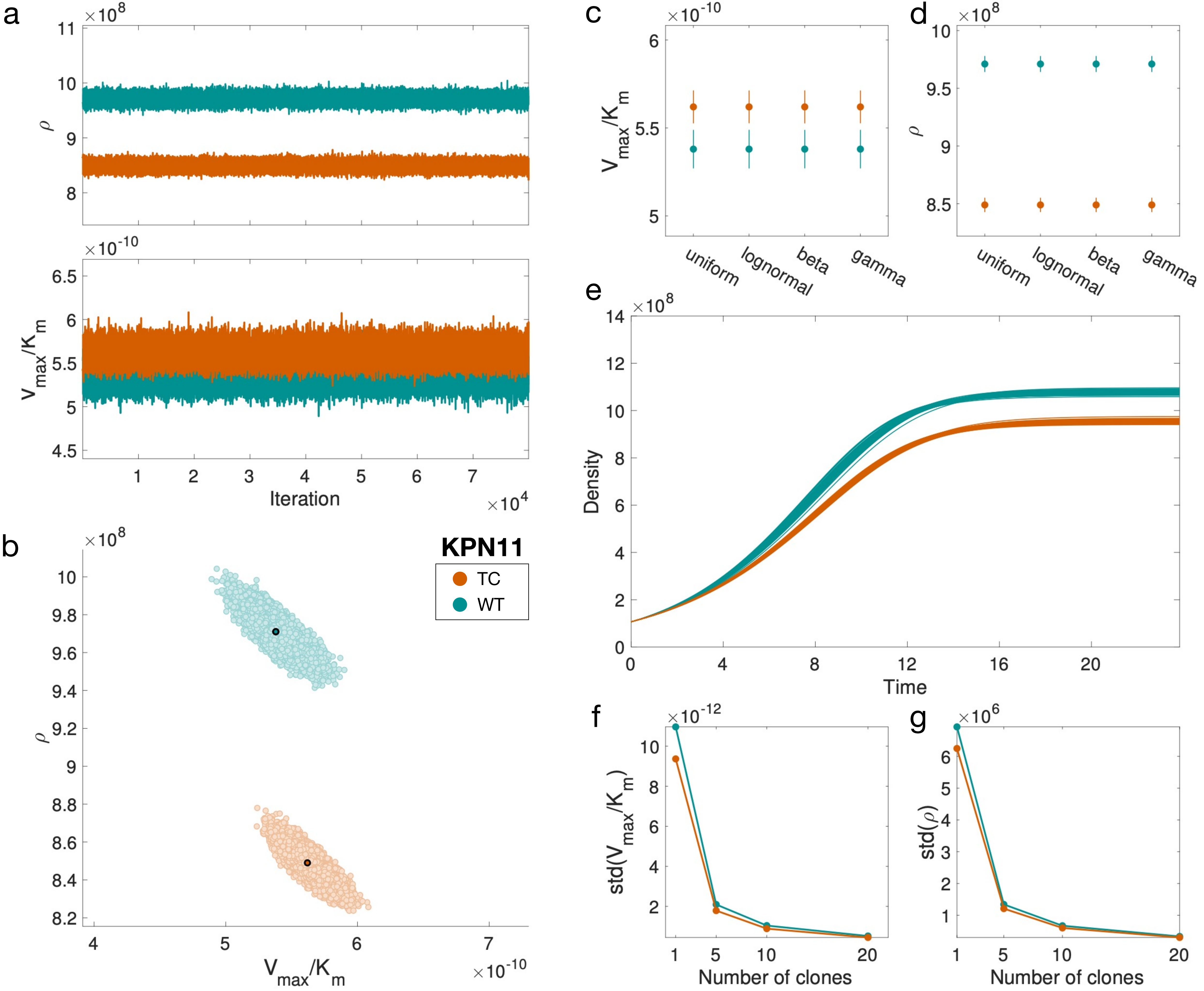

a
ρ
Iteration
V_{max}/K_m
b
KPN11
TC
WT
ρ
V_{max}/K_m
c
V_{max}/K_m
uniform
lognormal
beta
gamma
d
ρ
uniform
lognormal
beta
gamma
e
Density
Time
f
std(V_{max}/K_m)
Number of clones
g
std(ρ)
Number of clones

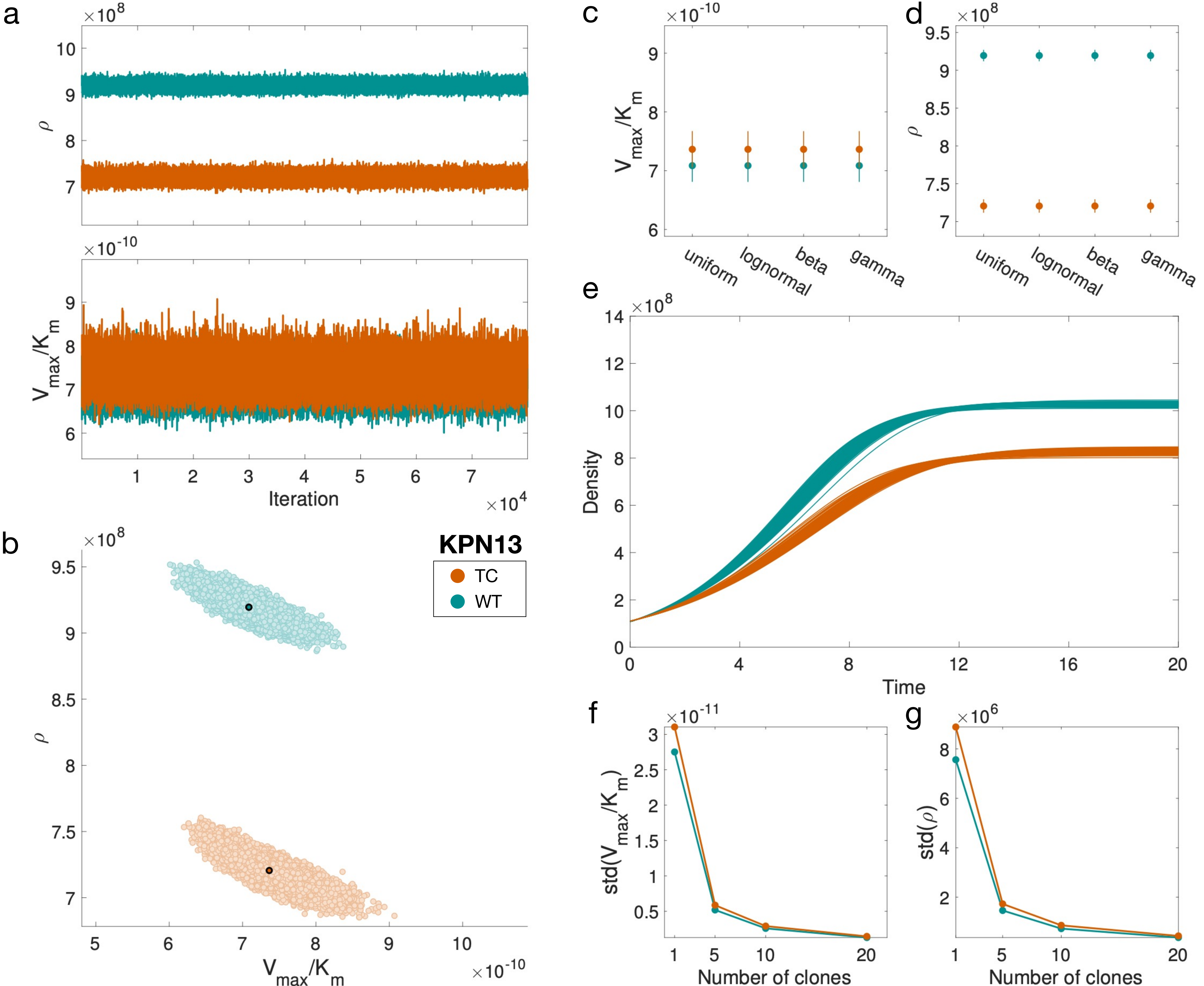

a
b
c
d
e
f
g
KPN13
TC
WT
ρ
V_{max}/K_m
Iteration
$\times 10^4$
$\times 10^8$
$\times 10^{-10}$
uniform
lognormal
beta
gamma
Density
Time
$std(V_{max}/K_m)$
$std(\rho)$
Number of clones
$\times 10^{-11}$
$\times 10^6$

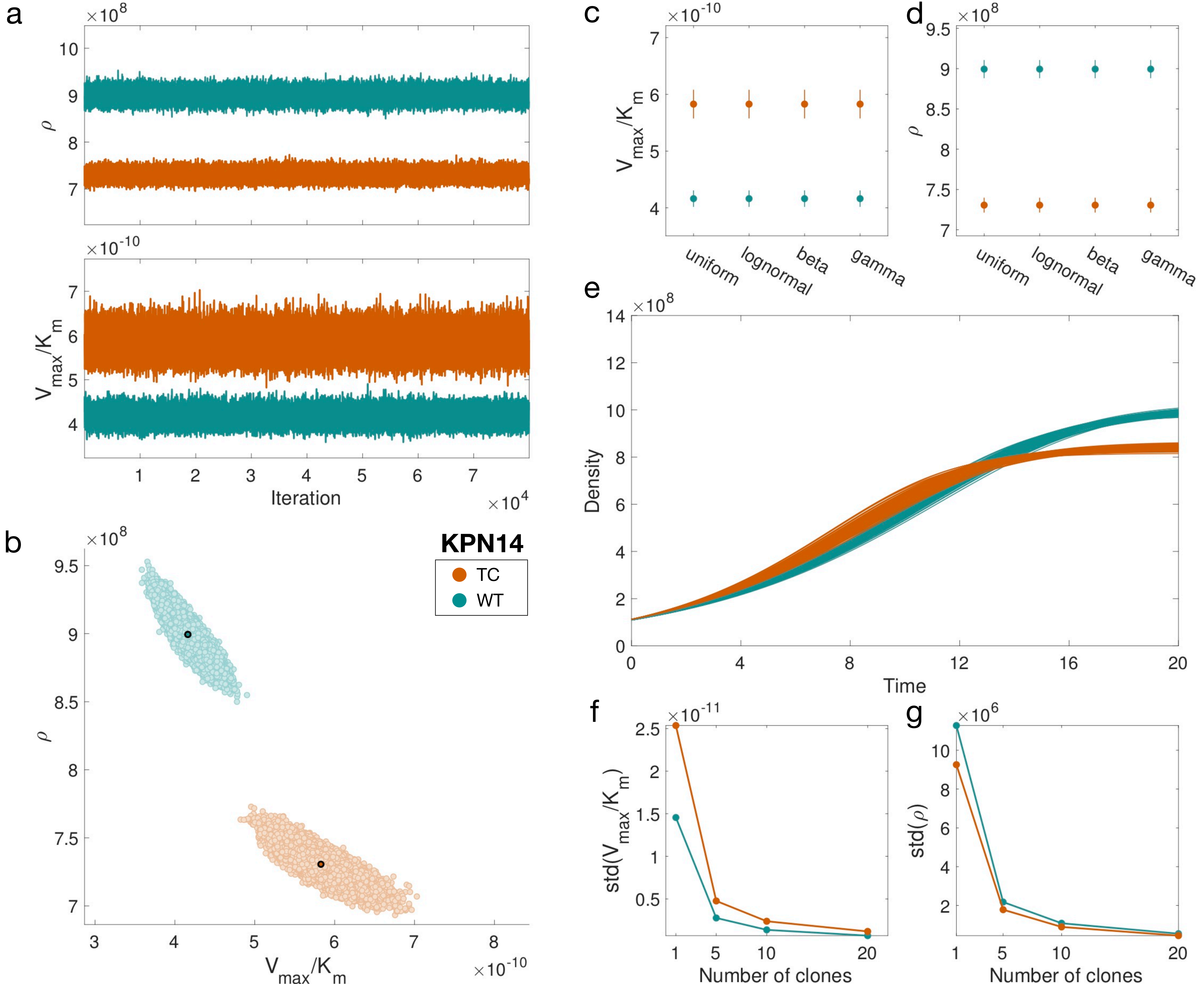

a
b
c
d
e
f
g
KPN14
TC
WT
Iteration
Density
Time
Number of clones
uniform
lognormal
beta
gamma

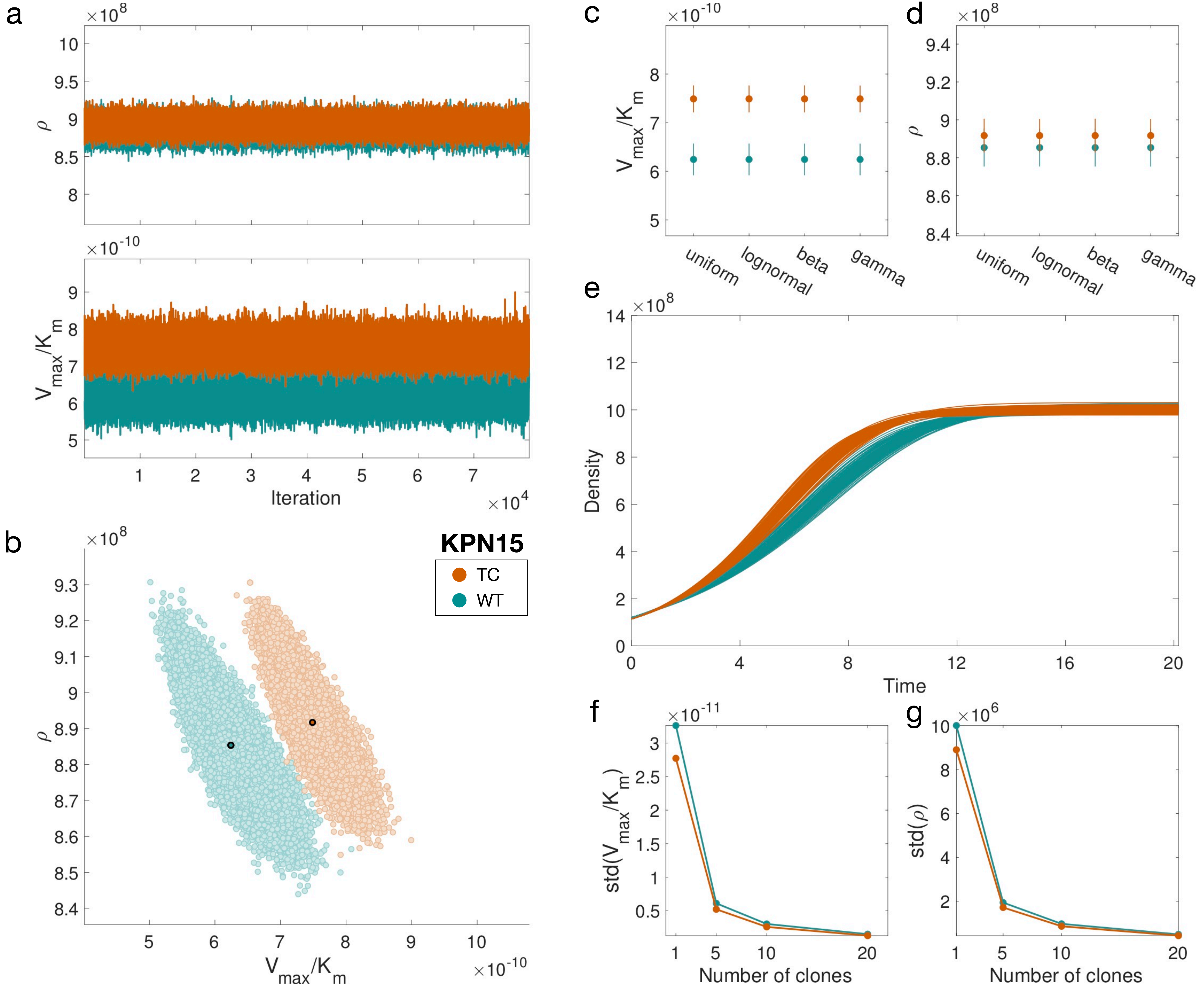

a
$\times 10^8$
ρ
$\times 10^{-10}$
V_{max}/K_m
Iteration
$\times 10^4$
b
KPN15
TC
WT
ρ
V_{max}/K_m
$\times 10^{-10}$
c
$\times 10^{-10}$
V_{max}/K_m
uniform
lognormal
beta
gamma
d
$\times 10^8$
ρ
uniform
lognormal
beta
gamma
e
$\times 10^8$
Density
Time
f
$\times 10^{-11}$
$std(V_{max}/K_m)$
Number of clones
g
$\times 10^6$
$std(\rho)$
Number of clones

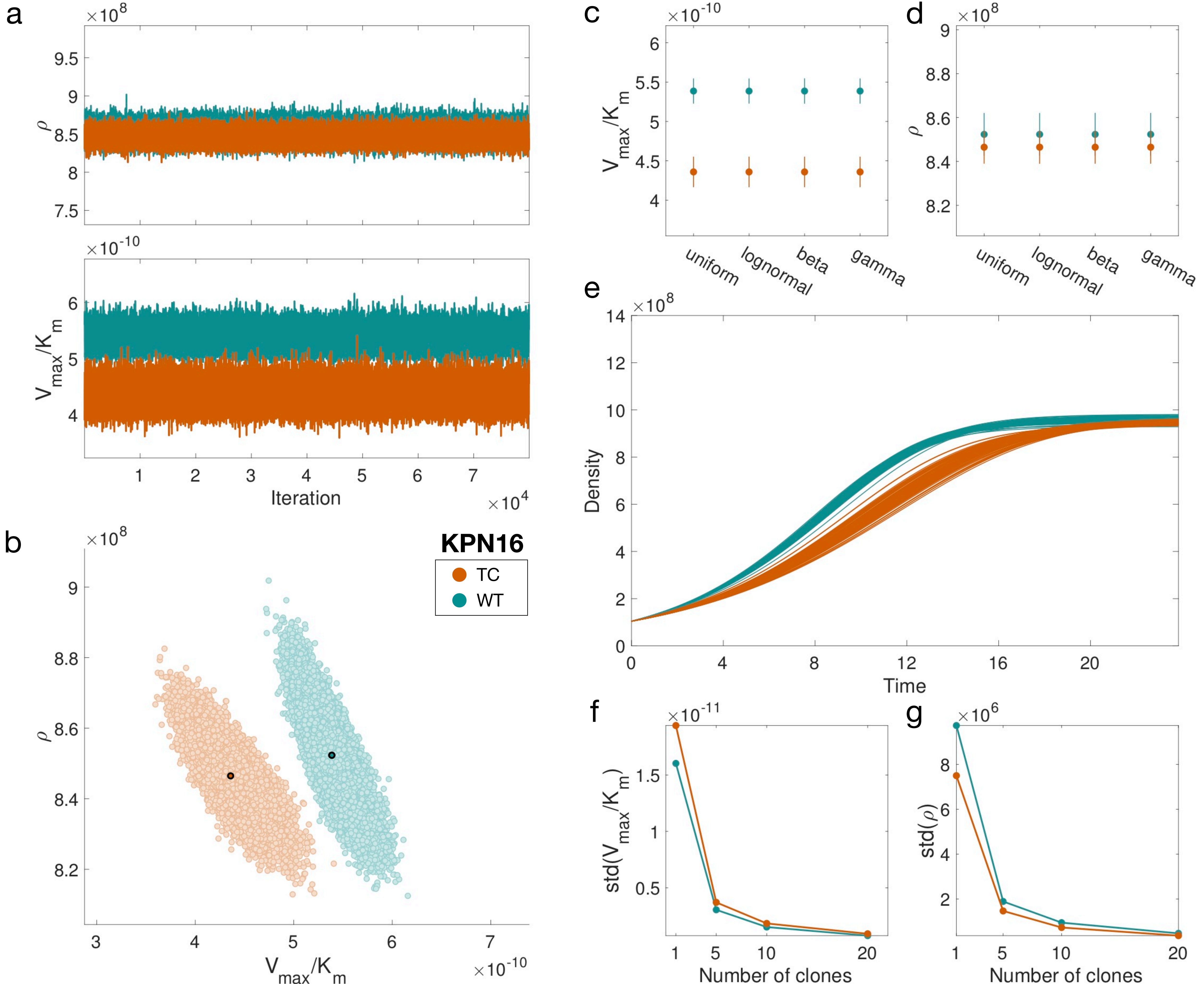
a
ρ
$\times 10^8$
V_{max}/K_m
$\times 10^{-10}$
Iteration
$\times 10^4$
b
KPN16
TC
WT
c
d
uniform
lognormal
beta
gamma
e
Density
Time
f
std(V_{max}/K_m)
$\times 10^{-11}$
g
std(ρ)
$\times 10^6$
Number of clones

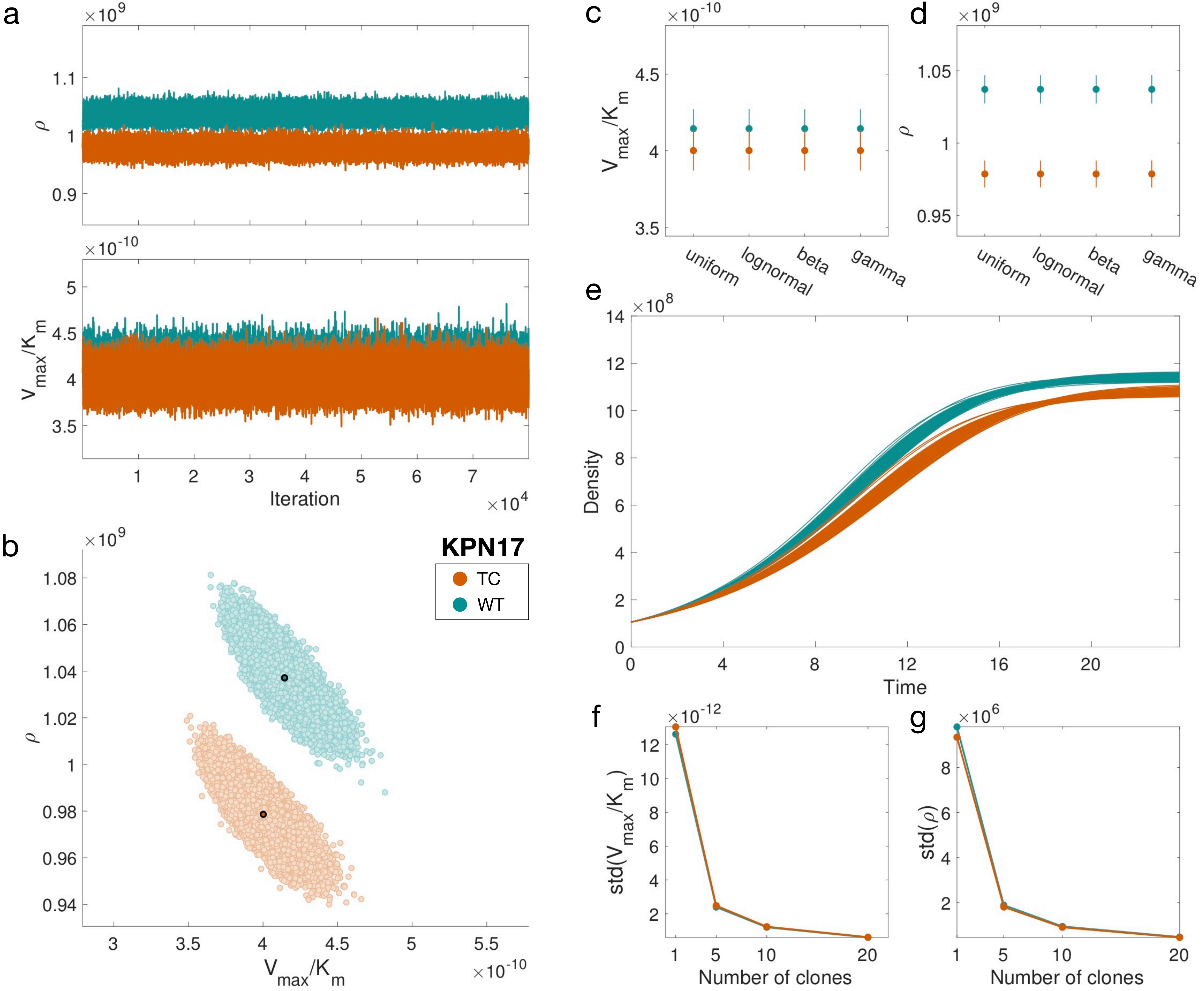

a
b
c
d
e
f
g
KPN17
TC
WT
ρ
V_{max}/K_m
Iteration
Density
Time
uniform
lognormal
beta
gamma
$std(V_{max}/K_m)$
std(ρ)
Number of clones

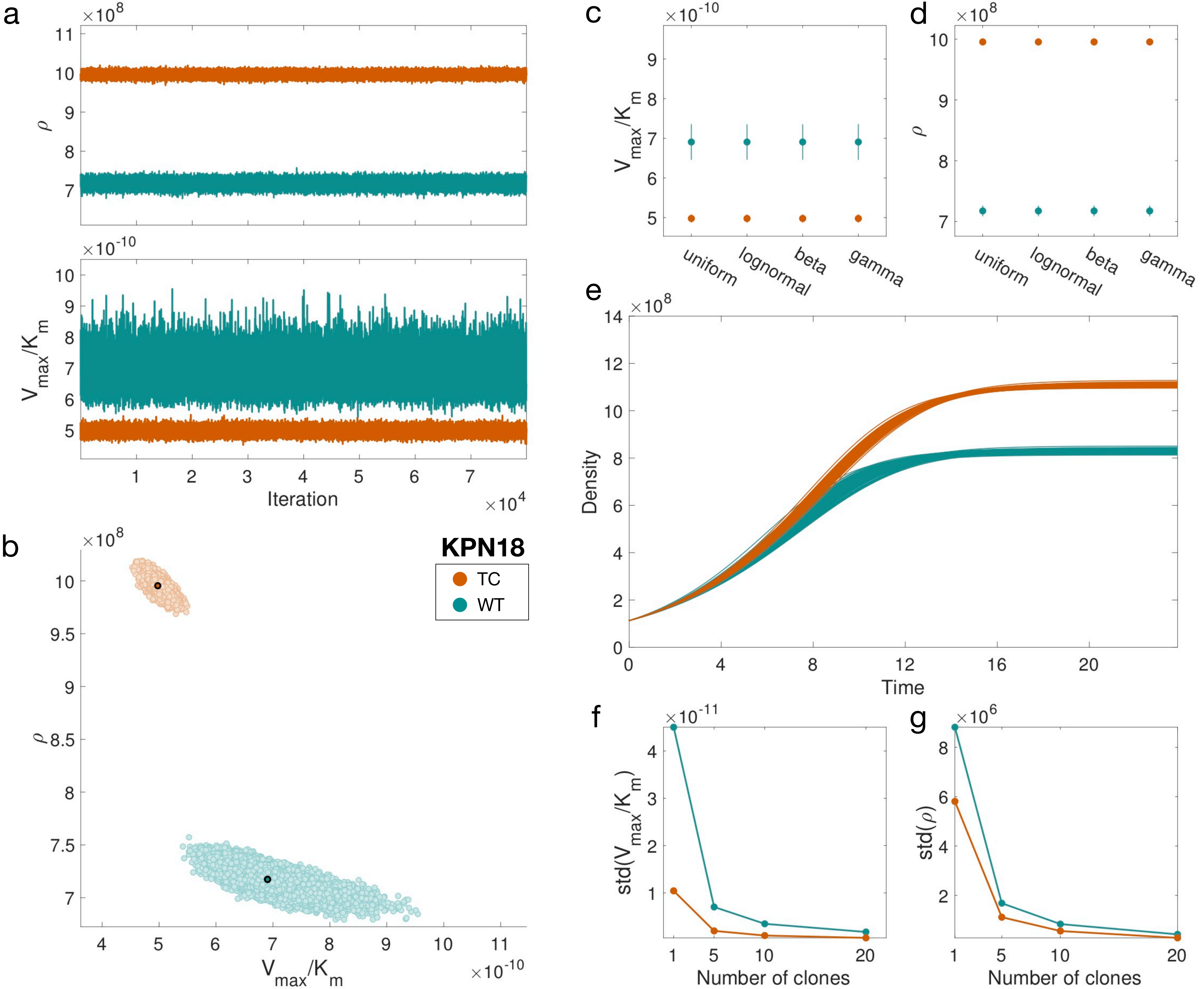

a
b
c
d
e
f
g
KPN18
TC
WT
Iteration
Density
Time
Number of clones
uniform
lognormal
beta
gamma

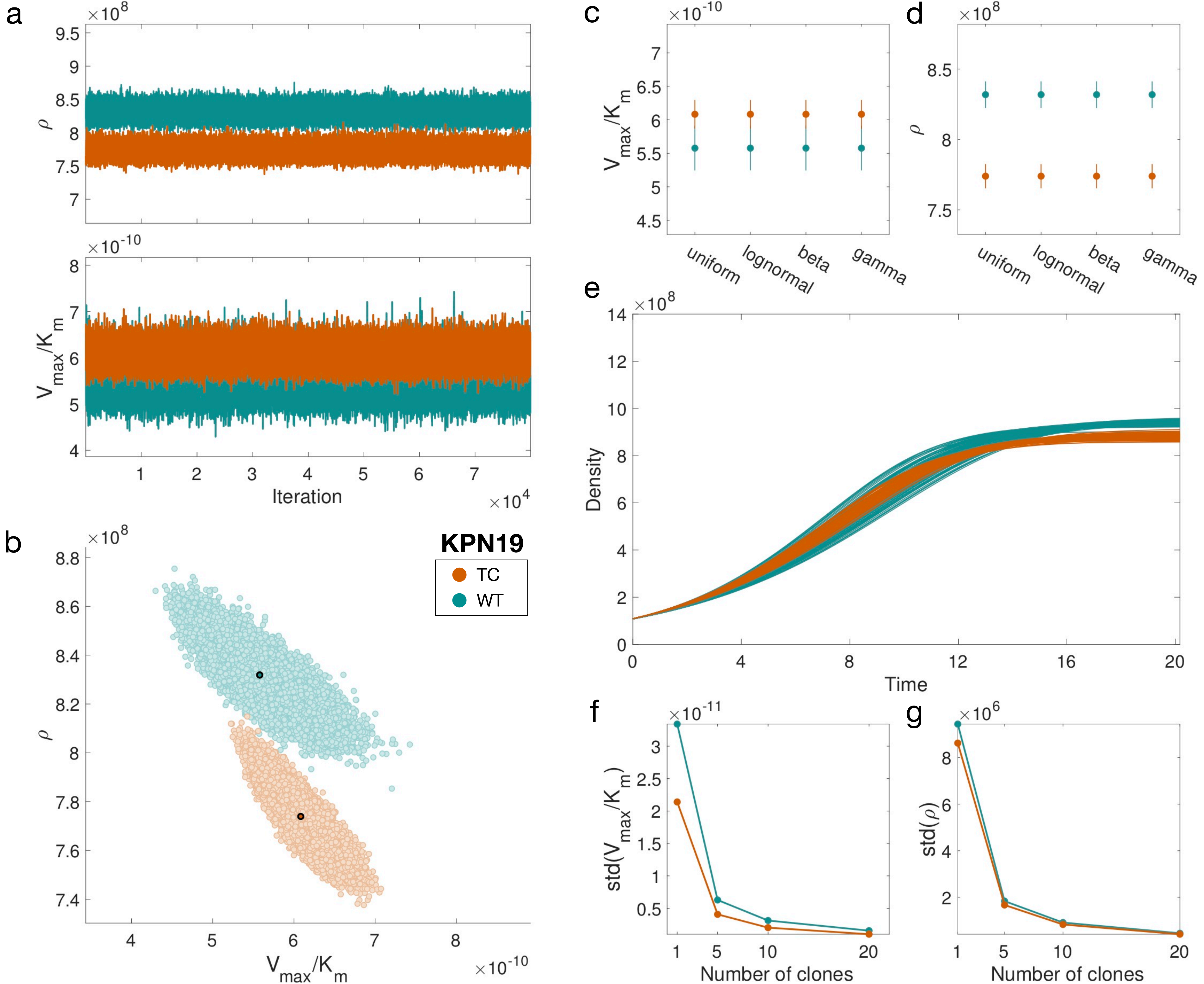

a
b
c
d
e
f
g
KPN19
TC
WT
Iteration
Density
Time
Number of clones
uniform
lognormal
beta
gamma

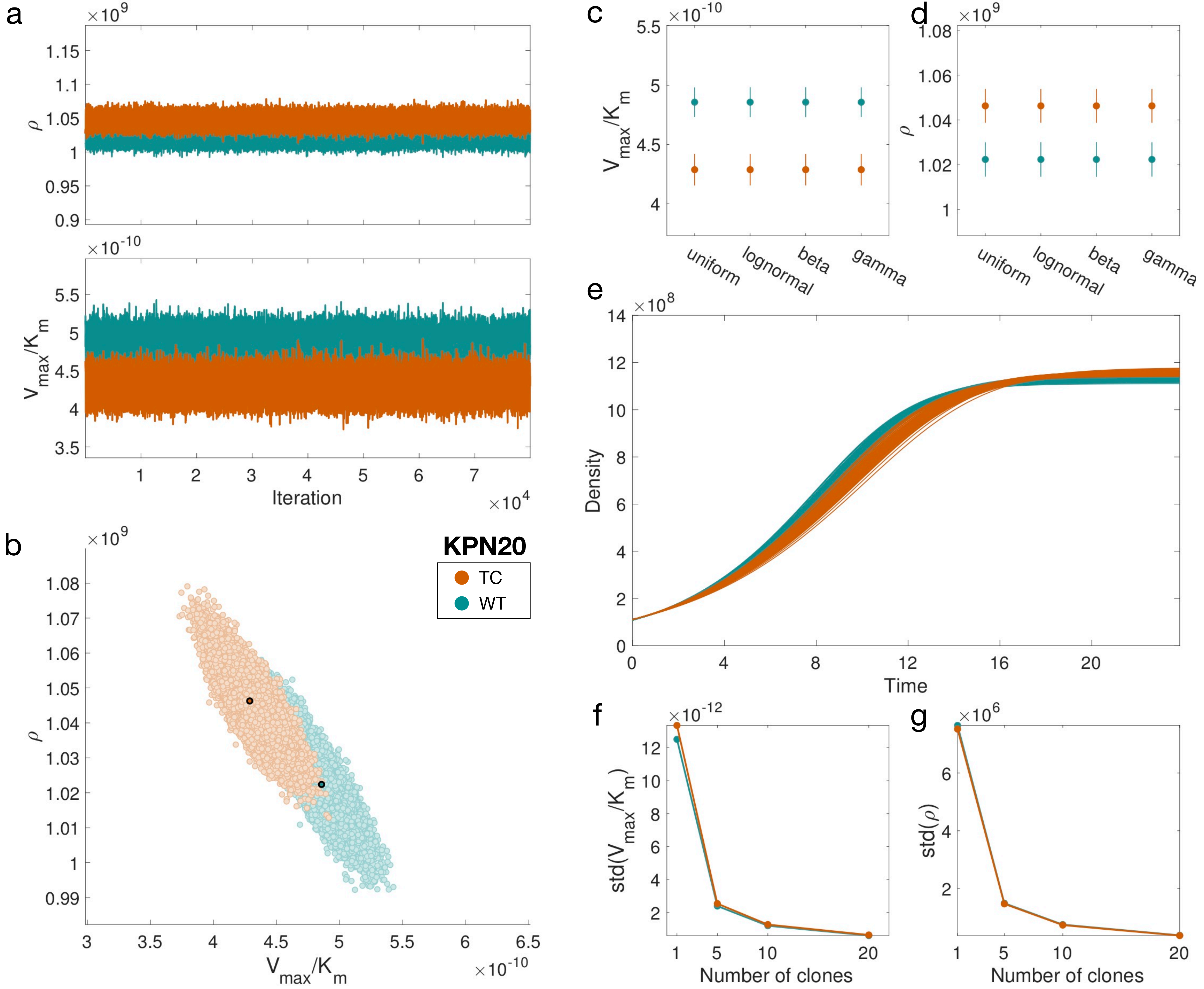

a
b
c
d
e
f
g
KPN20
TC
WT
ρ
Vmax/Km
Iteration
×10^4
uniform
lognormal
beta
gamma
Density
Time
std(Vmax/Km)
std(ρ)
Number of clones

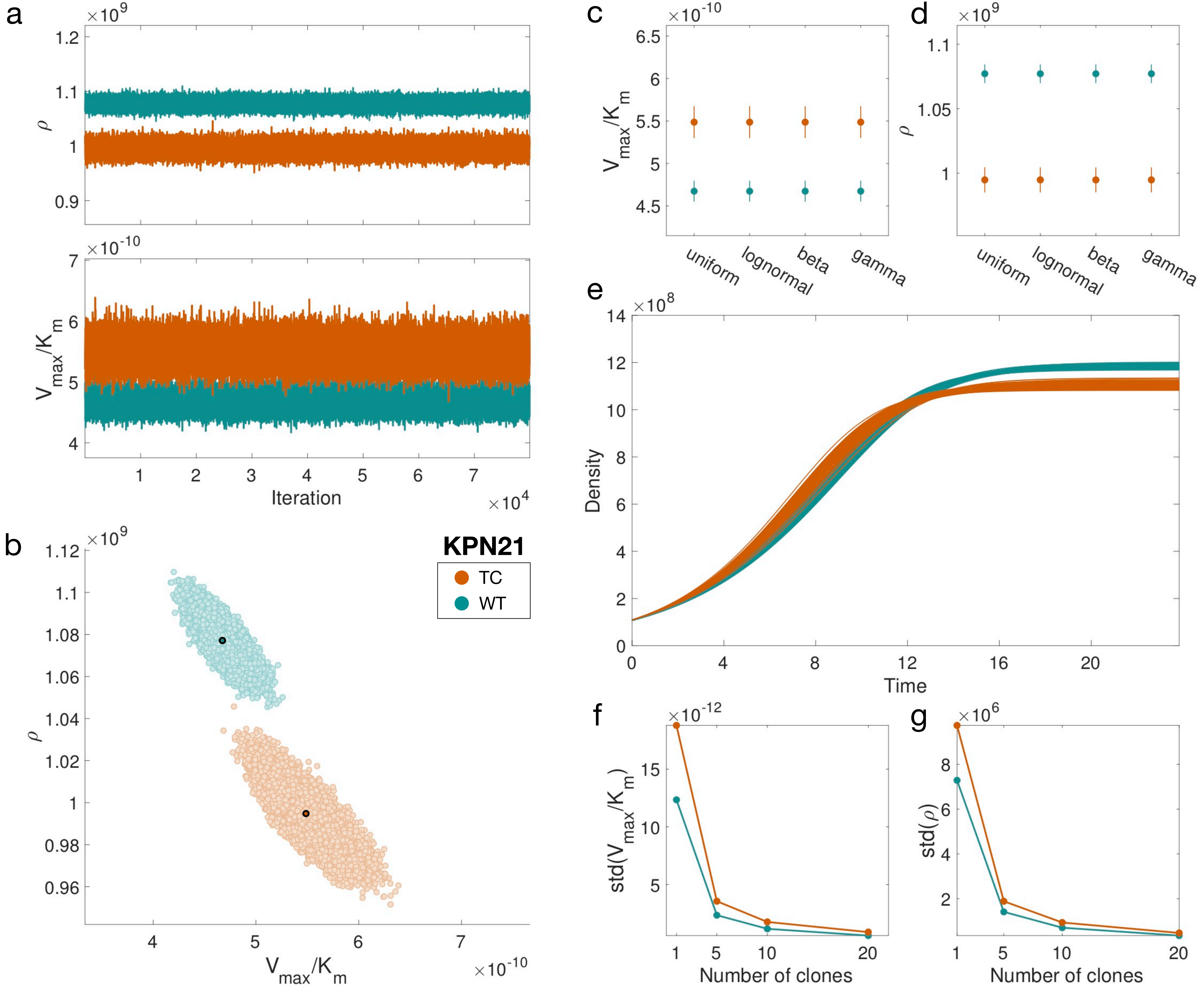
a
b
c
d
e
f
g
KPN21
TC
WT
Iteration
Density
Time
Number of clones
uniform
lognormal
beta
gamma

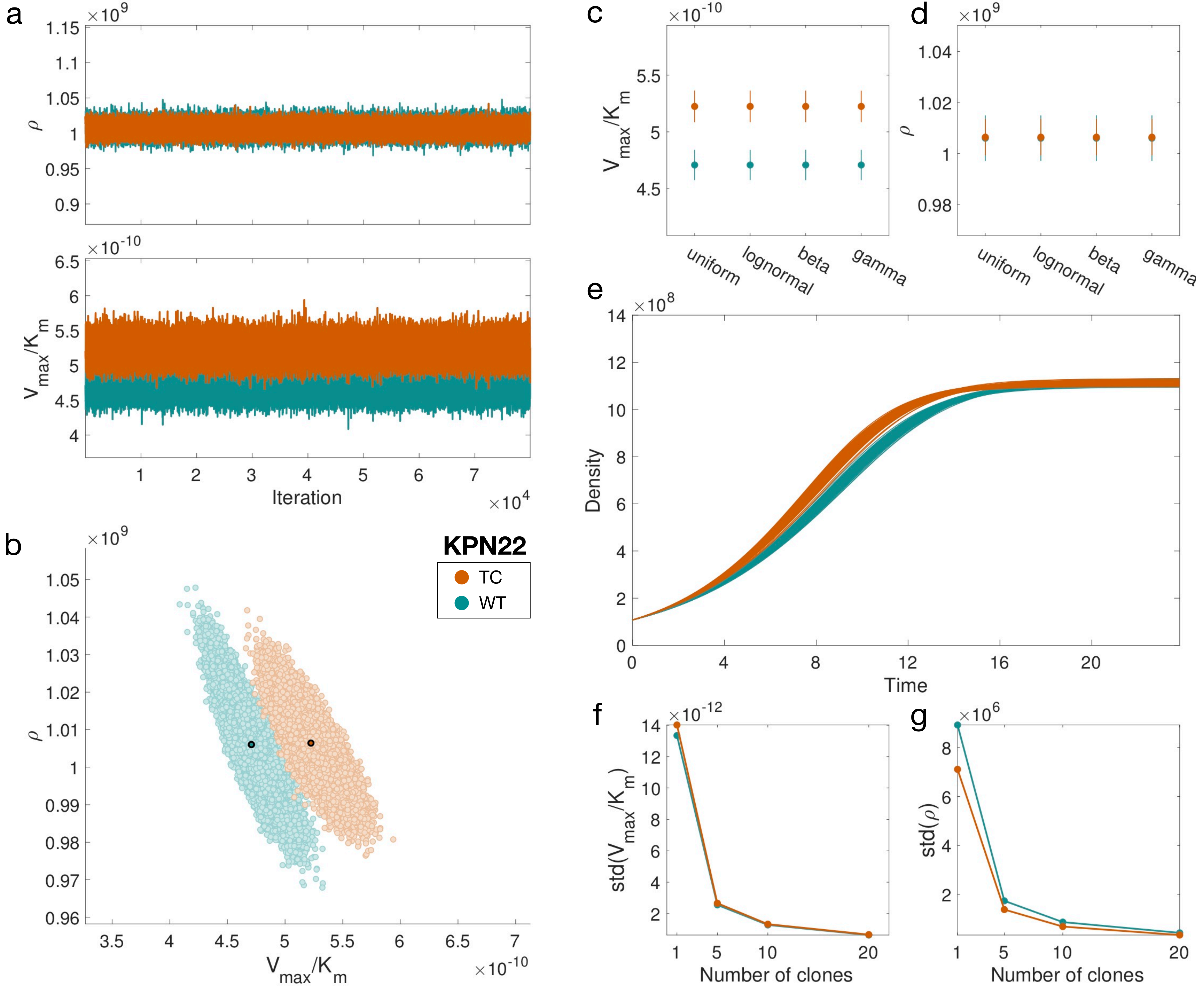
a
b
c
d
e
f
g
KPN22
TC
WT
Iteration
Density
Time
Number of clones
uniform
lognormal
beta
gamma

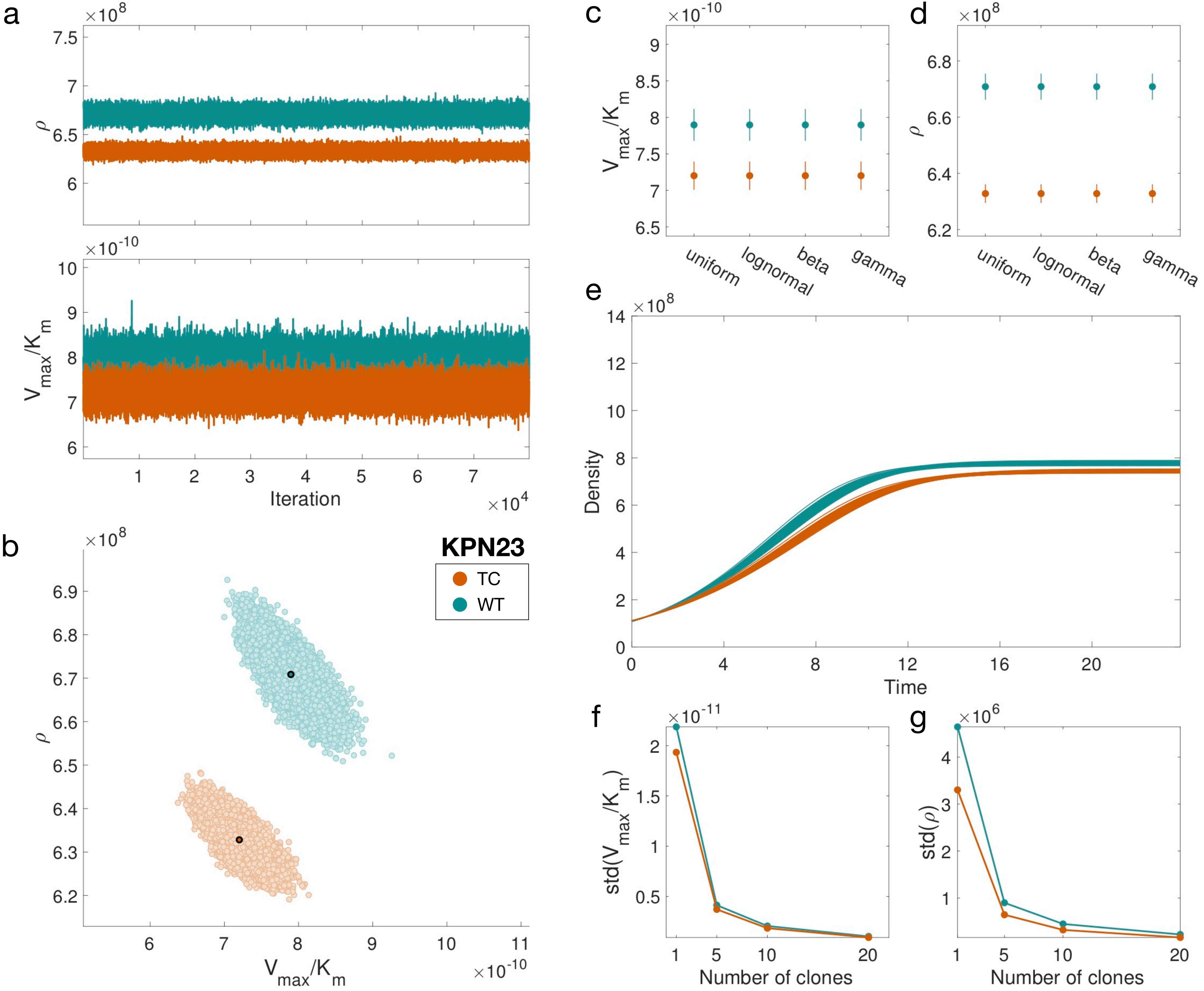
a
b
c
d
e
f
g
KPN23
TC
WT
ρ
V_{max}/K_m
Iteration
Density
Time
uniform
lognormal
beta
gamma
$std(V_{max}/K_m)$
std(ρ)
Number of clones
$\times10^8$
$\times10^{-10}$
$\times10^4$
$\times10^{-11}$
$\times10^6$

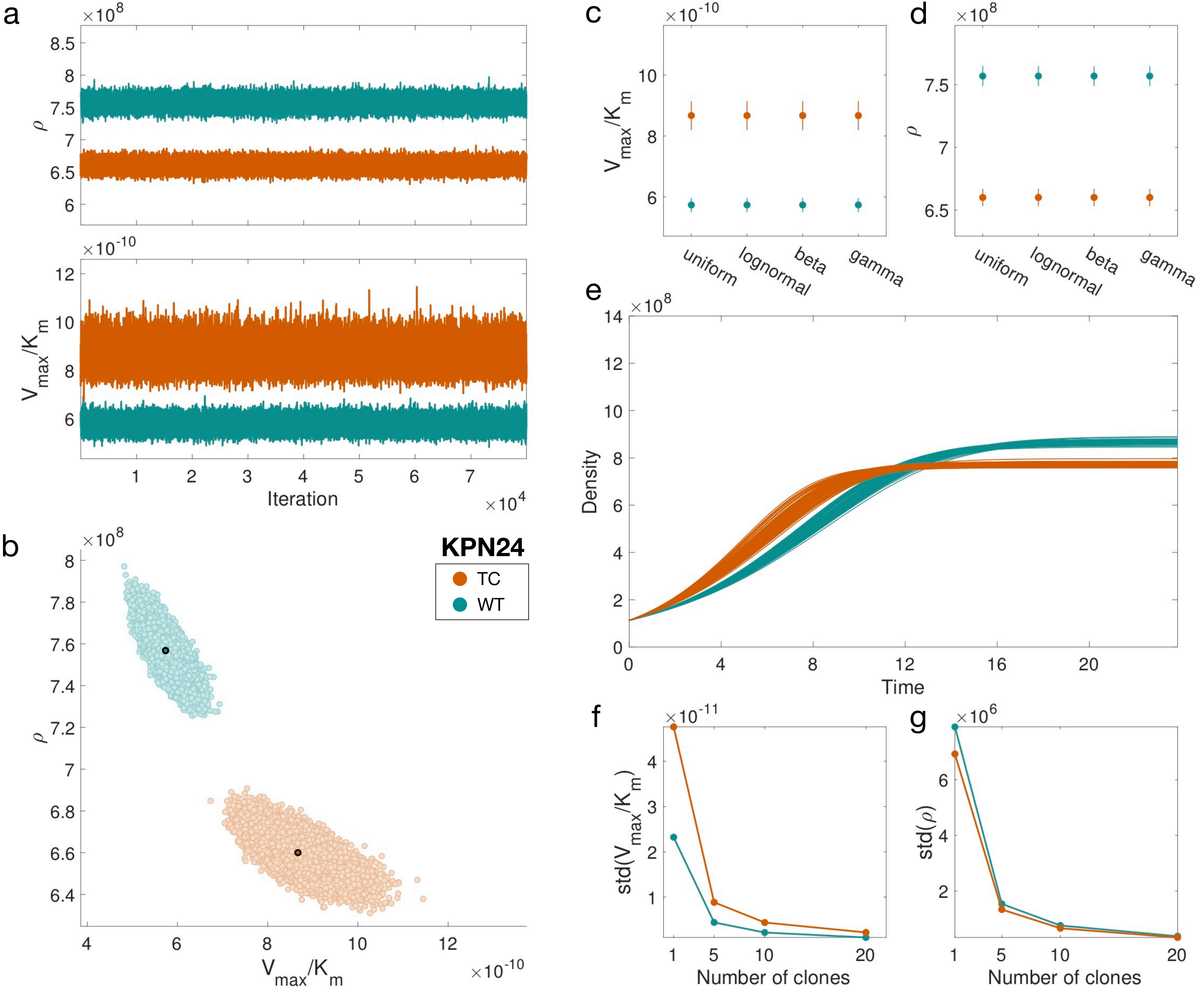

a
b
c
d
e
f
g
KPN24
TC
WT
ρ
V_{max}/K_m
Iteration
Time
Density
std(V_{max}/K_m)
std(ρ)
Number of clones
uniform
lognormal
beta
gamma

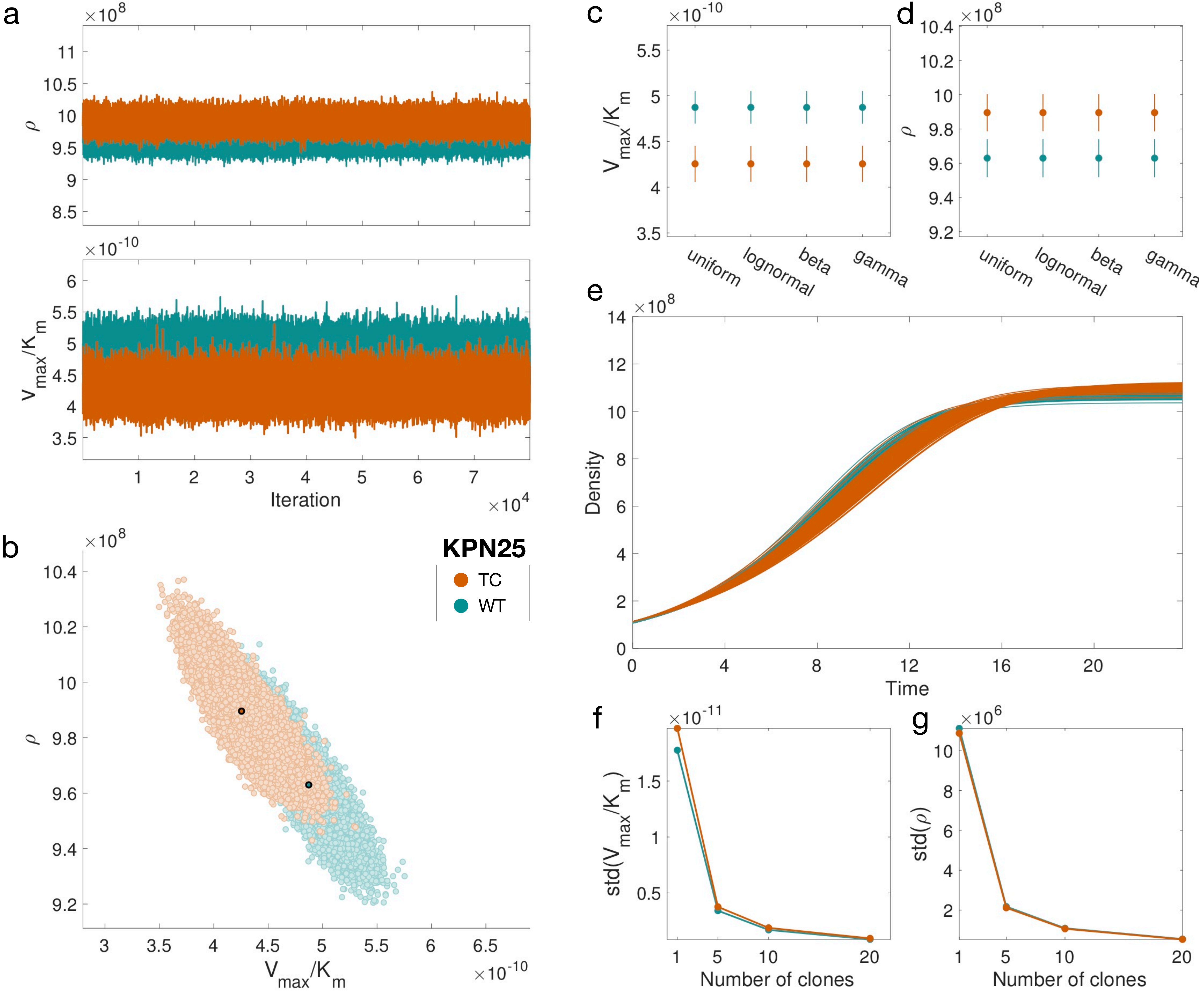

a
b
c
d
e
f
g
KPN25
TC
WT
ρ
Iteration
Vmax/Km
uniform
lognormal
beta
gamma
Density
Time
std(Vmax/Km)
std(ρ)
Number of clones
