# Supplementary material for: Variability of plasmid fitness effects contributes to plasmid persistence in bacterial communities
Source: Nat Commun. 2021 May 11;12:2653. doi: 10.1038/s41467-021-22849-y (PMC8113577; doi:10.1038/s41467-021-22849-y)
Supplement: Supplementary file 3 — Reporting Summary [file 41467_2021_22849_MOESM3_ESM.pdf]

Corresponding author(s): Alvaro San Millan, Rafael Peña-Miller

Last updated by author(s): Mar 16, 2021

Reporting Summary

Nature Research wishes to improve the reproducibility of the work that we publish. This form provides structure for consistency and transparency in reporting. For further information on Nature Research policies, see our Editorial Policies and the Editorial Policy Checklist.

Statistics

For all statistical analyses, confirm that the following items are present in the figure legend, table legend, main text, or Methods section.

n/a	Confirmed	
☐	☒	The exact sample size (*n*) for each experimental group/condition, given as a discrete number and unit of measurement
☐	☒	A statement on whether measurements were taken from distinct samples or whether the same sample was measured repeatedly
☐	☒	The statistical test(s) used AND whether they are one- or two-sided<br>*Only common tests should be described solely by name; describe more complex techniques in the Methods section.*
☒	☐	A description of all covariates tested
☐	☒	A description of any assumptions or corrections, such as tests of normality and adjustment for multiple comparisons
☐	☒	A full description of the statistical parameters including central tendency (e.g. means) or other basic estimates (e.g. regression coefficient) AND variation (e.g. standard deviation) or associated estimates of uncertainty (e.g. confidence intervals)
☐	☒	For null hypothesis testing, the test statistic (e.g. *F*, *t*, *r*) with confidence intervals, effect sizes, degrees of freedom and *P* value noted<br>*Give P values as exact values whenever suitable.*
☐	☒	For Bayesian analysis, information on the choice of priors and Markov chain Monte Carlo settings
☐	☒	For hierarchical and complex designs, identification of the appropriate level for tests and full reporting of outcomes
☐	☒	Estimates of effect sizes (e.g. Cohen's *d*, Pearson's *r*), indicating how they were calculated

Our web collection on statistics for biologists contains articles on many of the points above.

Software and code

Policy information about availability of computer code

Data collection	Statistical analyses were performed using R (v. 3.5.0).<br>Software used:<br>Trimmomatic v0.33 tool<br>SPAdes v3.9.0<br>QUAST v4.6.0<br>the multilocus sequence-typing (MLST) tool (https://github.com/tseemann/mlst).<br>PlasmidFinder 2.1<br>ResFinder 3.2<br>Snippy v3.1 (https://github.com/tseemann/snippy)<br>Prokka 1.14.6<br>National Center for Biotechnology Information (NCBI) Prokaryotic Genome Annotation Pipeline<br>Mash v2.0<br>mashtree v0.33 (https://github.com/tseemann/snippy).<br>IQ-TREE<br>phylotools package in R (https://github.com/helixcn/phylotools)<br>iTOL tool<br>AccNET
Data analysis	Code used:<br>https://github.com/tseemann/mlst<br>https://github.com/tseemann/snippy<br>https://github.com/helixcn/phylotools

https://github.com/tseemann/mlst

For manuscripts utilizing custom algorithms or software that are central to the research but not yet described in published literature, software must be made available to editors and reviewers. We strongly encourage code deposition in a community repository (e.g. GitHub). See the Nature Research guidelines for submitting code & software for further information.

Data

Policy information about availability of data

All manuscripts must include a data availability statement. This statement should provide the following information, where applicable:

- Accession codes, unique identifiers, or web links for publicly available datasets
- A list of figures that have associated raw data
- A description of any restrictions on data availability

The sequences generated and analysed during the current study and the annotated genomes of the isolates under study are available in the Sequence Read Archive (SRA), BioProject ID: PRJNA641166, https://www.ncbi.nlm.nih.gov/sra/PRJNA641166.
The authors declare that the data supporting the findings of this study are available within the paper and its supplementary information files. The code generated during the current study is available in GitHub:
San Millan, A., Peña-Miller, R., Toll-Riera, M., Halbert, Z., McLean, A., Cooper, B.S., MacLean, R. C. pNUK73: A Metropolis-Hastings MCMC implementation used to fit a bacterial growth curve. figshare. http://dx.doi.org/10.6084/m9.figshare.1127995 Retrieved Aug 05 (2014).
Alonso-del Valle, A., León-Sampedro, R., Rodríguez-Beltrán, J., DelaFuente, J., Hernández-García, M., Ruiz-Garbajosa, P., Cantón, R., Peña-Miller, R. and San Millán, A. pOXA48: Variability of plasmid fitness effects contributes to plasmid persistence in bacterial communities. Zenodo. http://dx.doi.org/10.5281/zenodo.4605352. Retrieved Mar 15 (2021).

Field-specific reporting

Please select the one below that is the best fit for your research. If you are not sure, read the appropriate sections before making your selection.

☐ Life sciences ☐ Behavioural & social sciences ☒ Ecological, evolutionary & environmental sciences

For a reference copy of the document with all sections, see nature.com/documents/nr-reporting-summary-flat.pdf

Ecological, evolutionary & environmental sciences study design

All studies must disclose on these points even when the disclosure is negative.

Study description

We studied the distribution of fitness efects of plasmid pOXA-48 in 50 clinical enterobacteria strains using different experimental methods and a mathematical model. We selected 50 representative ESBL-producing clones form the R-GNOSIS collection (Supplementary Data 1). This collection was constructed in our hospital as part of an active surveillance-screening program for detecting patients colonised by ESBL/carbapenemase-producing enterobacteria, from March 4th, 2014, to March 31st, 2016 (R-GNOSIS-FP7-HEALTH-F3-2011-282512, www.r-gnosis.eu/, approved by the Ramón y Cajal University Hospital Ethics Committee, Reference 251/13). The screening included a total of 28,089 samples from 9,275 patients admitted at 4 different wards (gastroenterology, neurosurgery, pneumology and urology) in the Ramon y Cajal University Hospital (Madrid, Spain). The characterisation of samples was performed during the R-GNOSIS study period; rectal swabs were plated on Chromo ID-ESBL and Chrom-CARB/OXA-48 selective agar media (BioMérieux, France) and bacterial colonies able to grow on these media were identified by MALDI-TOF MS (Bruker Daltonics, Germany) and further characterized by pulsed-field gel electrophoresis (PFGE). For the present study, we selected 25 E. coli and 25 K. pneumoniae ESBL-producing isolates from the R-GNOSIS collection. The strains were representative of E. coli and K. pneumoniae diversity in the R-GNOSIS collection (randomly chosen form the most common pulsed-field gel electrophoresis profiles), they did not carry any carbapenemase gene and they were recovered from patients not colonised by other pOXA-48-carrying clones.

Research sample

For the present study, we selected 25 E. coli and 25 K. pneumoniae ESBL-producing isolates from the R-GNOSIS collection. The strains were representative of E. coli and K. pneumoniae diversity in the R-GNOSIS collection (randomly chosen form the most common pulsed-field gel electrophoresis profiles), they did not carry any carbapenemase gene and they were recovered from patients not colonised by other pOXA-48-carrying clones.

Sampling strategy

We selected 50 representative ESBL-producing clones form the R-GNOSIS collection (Supplementary Table 1). For the present study, we selected 25 E. coli and 25 K. pneumoniae ESBL-producing isolates from the R-GNOSIS collection. We used the following criteria: i) the strains were representative of the E. coli and K. pneumoniae in the R-GNOSIS collection (randomly chosen form the most common PFGE profiles) and ii) they did not carry any carbapenemase gene. To construct the transconjugants, we used the most common pOXA-48 plasmid variant from the R-GNOSIS collection in our hospital, according to plasmid genetic sequence (pOXA-48_K8, accession number MT441554). No sample size calculation was performed, as we didi not have a priori estimation of the expected distribution of fitness effects.

Data collection

The screening included a total of 28,089 samples from 9,275 patients admitted at 4 different wards (gastroenterology, neurosurgery, pneumology and urology) in the Ramon y Cajal University Hospital (Madrid, Spain), collected manually. The characterisation of samples was performed during the R-GNOSIS study period; rectal swabs were plated on Chromo ID-ESBL and Chrom-CARB/OXA-48 selective agar media (BioMérieux, France) and bacterial colonies able to grow on these media were identified by MALDI-TOF MS (Bruker Daltonics, Germany) and further characterized by pulsed-field gel electrophoresis (PFGE). The data was collected manually (samples), using flow cytometry (competitions, CytoFLEX Platform, Beckman Coulter Life Sciences, IN, US), plate readers (optical

	density, growth curves, Synergy HTX Multi-Mode Reader, BioTek Instruments, Inc, VT, USA) and sequencing platforms (Illumina HiSeq4000 platform).
Timing and spatial scale	R-GNOSIS collection was constructed in our hospital as part of an active surveillance-screening program for detecting patients colonised by ESBL/carbapenemase-producing enterobacteria, from March 4th, 2014, to March 31st, 2016 (R-GNOSIS-FP7-HEALTH-F3-2011-282512, www.r-gnosis.eu/). . The screening included a total of 28,089 samples from 9,275 patients admitted at 4 different wards (gastroenterology, neurosurgery, pneumology and urology) in the Ramon y Cajal University Hospital (Madrid, Spain). In this study we used these samples, so the time and saptial scale are dictated by the R-GNOSIS collection.
Data exclusions	Three genomes were dropped from the genomic analysis because of the poor quality of the sequences (2 E. coli - Ec09, Ec17- and 1 K. pneumoniae - Kpn05 -).
Reproducibility	We performed several biological replictates of each experiment (spcified in the manuscript)
Randomization	We randomly selected 25 Escherichia coli strains and 25 Klebsiella pneumoniae strains, chosen form the most common PFGE profiles in the R-GNOSIS collection. Organisms were allocated in the different groups according to their sepcies identification.
Blinding	The investigators performing the experiments (AAdV) was not blinded as this is not applicable for the methods used.

Did the study involve field work? ☐ Yes ☒ No

Reporting for specific materials, systems and methods

We require information from authors about some types of materials, experimental systems and methods used in many studies. Here, indicate whether each material, system or method listed is relevant to your study. If you are not sure if a list item applies to your research, read the appropriate section before selecting a response.

Materials & experimental systems

n/a	Involved in the study
☒	☐ Antibodies
☒	☐ Eukaryotic cell lines
☒	☐ Palaeontology and archaeology
☒	☐ Animals and other organisms
☒	☐ Human research participants
☒	☐ Clinical data
☒	☐ Dual use research of concern

Methods

n/a	Involved in the study
☒	☐ ChIP-seq
☐	☒ Flow cytometry
☒	☐ MRI-based neuroimaging

Flow Cytometry

Plots

Confirm that:

☒ The axis labels state the marker and fluorochrome used (e.g. CD4-FITC).

☒ The axis scales are clearly visible. Include numbers along axes only for bottom left plot of group (a 'group' is an analysis of identical markers).

☒ All plots are contour plots with outliers or pseudocolor plots.

☒ A numerical value for number of cells or percentage (with statistics) is provided.

Methodology

Sample preparation	We used flow cytometry for the competition assays .<br>Five biological replicates of each competition were performed. Pre-cultures were incubated overnight in LB in 96-well plates at 225 rpm an 37ºC, then mixed 1:1 and diluted 10,000-fold in 200 µl of fresh LB in in 96-well plates. Mixtures were competed for 24 h in LB at 37ºC and 250 rpm. To determine the initial proportions, initial mixes were diluted 2,000-fold in 200 µl of NaCl 0.9 % with L-arabinose 0.1 %, and incubated at 37 ºC at 250 rpm during 1.5 h to induce GFP expression. The measurements were performed via flow cytometry using a CytoFLEX Platform (Beckman Coulter Life Sciences, IN, US) with the following parameters: flow rate: 50 µl min−1, core size: 22 µm, events recorded per sample: 10,000. After 24 h of incubation, final proportions were determined as described above, after 2,000-fold dilution of the cultures.
Instrument	CytoFLEX (Beckman Coulter Life Sciences, IN, US)
Software	CytoFLEX Platform (Beckman Coulter Life Sciences, IN, US)
Cell population abundance	Parameters: flow rate: 50 µl min−1, core size: 22 µm, events recorded per sample: 10,000.

Gating strategy

FSC/SSC gating was performed using GFP-tagged strains (each wild-type strain carrying plasmid pBGC)

☒ Tick this box to confirm that a figure exemplifying the gating strategy is provided in the Supplementary Information.
