# Supplementary material for: Variability of plasmid fitness effects contributes to plasmid persistence in bacterial communities
Source: Nat Commun. 2021 May 11;12:2653. doi: 10.1038/s41467-021-22849-y (PMC8113577; doi:10.1038/s41467-021-22849-y)
Supplement: Supplementary file 4 — Description of Additional Supplementary Files [file 41467_2021_22849_MOESM4_ESM.pdf]

Description of Additional Supplementary Files

File Name: Supplementary Data 1
Description: Bacterial strains and plasmids used in this study.

File Name: Supplementary Data 2
Description: Disk diffusion antibiograms of the strains used in this study.

File Name: Supplementary Data 3
Description: Analysis of plasmid fitness effects across bacterial phylogeny.
First tab: Methods implemented in the *phyloSignal* function: Abouheif's C_{mean}, Moran's I index, Bloomberg's *K* and *K**, and Pagel's *λ*. These produce global measures of phylogenetic signal (*i.e.* across the whole phylogeny).
Second tab: we used the Local Indicator of Phylogenetic Association (LIPA) based on local Moran's I, which is meant to detect local hotspots of phylogenetic signal. LIPA, implemented in the *lipaMoran* function, computes local Moran's I indexes for each tip of the phylogeny and a non-parametric test to ascertain statistical significance (two-sided). We have included Source Data files for these figures.
